# Triterpenes and Pheophorbides from *Camellia ptilosperma* and Their Cytotoxicity, Photocytotoxicity, and Photodynamic Antibacterial Activity

**DOI:** 10.3390/molecules28207058

**Published:** 2023-10-12

**Authors:** Siyuan Ma, Mengling Weng, Ting Yang, Li Ge, Kedi Yang

**Affiliations:** 1 School of Chemistry & Chemical Engineering, Guangxi University, Nanning 530004, China; masiyuan@aliyun.com; 2 Key Laboratory of Sugarcane Biotechnology and Genetic Improvement (Guangxi), Ministry of Agriculture & Rural Affairs, Guangxi Key Laboratory of Sugarcane Genetic Improvement, Sugarcane Research Institute, Guangxi Academy of Agricultural Sciences, Nanning 530004, China; 3 Guangxi Fangcheng Golden Camellia National Nature Reserve Management Center, Fangchenggang 538021, China; 4 Medical College, Guangxi University, Nanning 530004, China

**Keywords:** *Camellia ptilosperma*, triterpene, pheophorbide, cytotoxicity, antibacterial activity, photodynamic therapy

## Abstract

Phytochemical investigation of the leaves of *Camellia ptilosperma* S. Y. Liang et Q. D. Chen led to the isolation of ten undescribed compounds, including six new triterpenes (**1**–**6**) and four new pheophorbide-related compounds (**7**–**10**). Meanwhile, the cytotoxic activity of the six triterpenes against six cancer cell lines was evaluated by MTT assay. Compound **2** showed potent cytotoxicity toward HepG2 cells with an IC_50_ value of 2.57 μM. Compounds **4** and **5** exhibited cytotoxicity against MDA-MB231 cells, with IC_50_ values of 11.31 and 5.52 μM, respectively. Additionally, the cytotoxicity of four new pheophorbides against these cancer cells was evaluated both in the presence and absence of light treatment. Compound **7** exhibited exceptional photocytotoxicity against Hela, MCF-7, and A549 cells, with IC_50_ values of 0.43 μM, 0.28 μM, and 0.92 μM, respectively. Compound **10** demonstrated significant photodynamic cytotoxic activity against BEL-7402 and HepG2 cells with IC_50_ values of 0.77 μM and 0.33 μM, respectively. The photodynamic antibacterial activity of **7**–**10** was also tested for *S. aureus*, *E. coli*, *K. pneumoniae*, and *P. aeruginosa* under direct illumination. Compounds **8** and **10** exhibited sensitivity to *E. coli* and demonstrated a photodynamic antibacterial effect, with a MIC value of 0.625 μM.

## 1. Introduction

Yellow camellia, a member of the Theaceae family, is an evergreen shrub or dungarunga that was first discovered in Fangchenggang, Guangxi, China in 1933. To date, more than 43 species, including 5 variants, of yellow camellias have been identified, with their primary distribution spanning southwest China and northern Vietnam [1]. Distinguished from common tea plants with their red, pink, and white flowers, yellow camellia has unique golden-yellow flowers due to which it is honored as ‘flora panda’ and ‘camellia queen’. The leaves and flowers of yellow camellias are commonly utilized in the preparation of a popular tea known as Jin-Hua-Cha among local communities. Furthermore, local communities have long employed yellow camellias as traditional remedies for conditions such as hypertension, sore throat, and cancer prevention. In 2010, the Chinese Ministry of Health included yellow camellia in the national list of new food resources, leading to the continued development of functional foods based on yellow camellia, and currently available products include oral solutions [2] and instant teas [3] with yellow camellia. *Camellia ptilosperma*, a renowned species of yellow camellia, was discovered in 1982 in Chongzuo, Guangxi. Unlike other yellow camellia, which bloom from September to November, *C. ptilosperma* blooms from July to the following April [4].

The biological activity research on yellow camellia has mainly focused on *C. tunghinensis* and *C. nitidissima*, and has documented the diverse pharmacological properties of phytochemicals or crude extracts derived from yellow camellia, encompassing antioxidant activity [5,6], anti-hypertensive [7] and hyperlipidemic effects [8,9], hypoglycemic effects [10,11], antibacterial effects [12], anticancer activity [13,14,15,16], and anti-depressive effects [17,18].

The chemical composition of *C. ptilosperma* is currently unknown. In view of its potential medicinal and economic value, there is a need for phytochemical research on it. According to the literature reports, the leaves of plants in *Theaceae* contain mainly polyphenols, terpenes, flavonoids, tannins, and chlorophylls [19,20,21,22]. Although polyphenols, flavonoids, and tannins have been extensively studied [23,24,25,26,27], there have been no relevant reports on pheophorbide in the last 5 years. Chlorophyll and pheophorbide belong to the porphyrin group and are potential photosensitizers [28,29,30]. In the presence of light, they can produce free radicals or reactive oxygen species, which have a powerful killing effect on bacteria, microorganisms, and viruses [31,32]. Many triterpenoids have shown anti-tumor activity [33,34,35]. Therefore, this study focuses on the triterpenoids and pheophorbides in *C. ptilosperma*. In the biological activity experiment section, the cytotoxic activity of triterpenoids against cancer cells, photocytotoxicity against cancer cells, and the photodynamic antibacterial activity of pheophorbides were evaluated.

## 2. Results and Discussion

**Figure 1:**
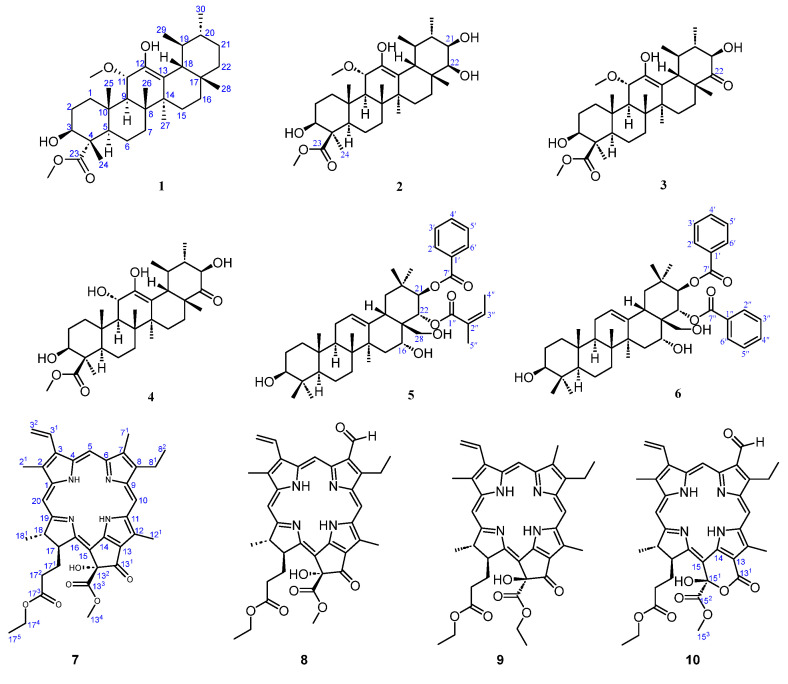
Structure of compounds **1**–**10** from the leaves of *C. ptilosperma*.

In this work, ten undescribed compounds, including six triterpenes (**1**–**6**, Figure 1) and four pheophorbides (**7**–**10**, Figure 1), were isolated from the leaves of *C. ptilosperma*. The cytotoxic activity of the six triterpenes against six cancer cell lines, namely Hela, MCF-7, BEL-7402, A549, HepG2, and MDA-MB-231, was evaluated by MTT assay. The photocytotoxic activity of four pheophorbides on the same human tumor cell lines was tested under both illuminated and non-illuminated conditions. Furthermore, the antimicrobial properties of pheophorbides were evaluated against a range of bacteria, including *S. aureus*, *E. coli*, *K. pneumoniae*, and *P. aeruginosa*.

**Figure 2:**
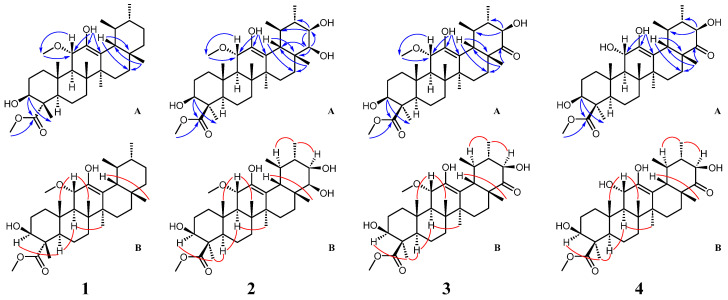
Key HMBC (A) and NOESY (B) correlations of compounds **1**–**4**.

Compound **1** was obtained as a colorless solid. Its molecular formula was C_32_H_52_O_5_ given by HR-ESI-MS (539.3718, [M+Na]^+^, calcd. for C_32_H_52_O_5_Na, 539.3712) with 7 degrees of unsaturation. Also, the Liebermann–Burchard reaction for **1** was positive, suggesting a triterpenoid structure. ^1^H NMR data (Table 1) demonstrated two distinctive methines at *δ*_H_ 0.92 (d, *J* = 6.6 Hz, H-29) and 0.93 (d, *J* = 6.4 Hz, H-30), which coincided with the ursane-type triterpene skeleton. The ^1^H and ^13^C NMR data (Table 1) revealed the presence of the other five methyl groups (*δ*_H_ 0.80, 1.09, 1.13, 1.18, and 1.21), one oxygenated methine group at *δ*_H_ 4.00 (dd, *J* = 11.5, 5.1 Hz) and two methoxyl groups at *δ*_H_ 3.18 (s) and 3.73 (s). A hydroxy group was located in C-3 (*δ*_C_ 75.3) based on the HMBC correlations (Figure 2) between H-3 (*δ*_H_ 4.00), C-23 (*δ*_C_ 178.1), and C-24 (*δ*_C_ 11.0). The relative configuration of the 3-OH was a β-orientation, which was established from the trans-diaxial coupling constant of H-3 (*J* = 11.5 Hz) and the NOESY correlations (Figure 2) of the proton signals at H-3 (*δ*_H_ 4.00), H-5 (*δ*_H_ 1.55), and H-9 (*δ*_H_ 1.94), respectively. The hydroxy group attached to C-12 was confirmed by the HMBC correlations of 12-OH (*δ*_H_ 4.53) with C-11 (*δ*_C_ 76.4), C-12 (*δ*_C_ 141.7), and C-13 (*δ*_C_ 118.3). The cross-peak from *δ*_H_ 3.18 (3H, s) to *δ*_C_ 76.4 (C-11) in the HMBC spectrum indicated a methoxyl group attached to C-11. In addition, a β-oriented H-11 could be deduced from the large ^3^*J* values of 10.4 Hz and NOESY correlations of H-11 (*δ*_H_ 4.26) with H-25 (*δ*_H_ 1.13) and H-26 (*δ*_H_ 1.09). The presence of a methyl ester group at C-4 was confirmed by the HMBC correlations between 23-OCH_3_ (*δ*_H_ 3.73) and C-23 (*δ*_C_ 178.1), as well as between H-3 (*δ*_H_ 4.00) and C-23 (*δ*_C_ 178.1). Thus, compound **1** was determined to be 11α-methoxy-3β,12-dihydroxyurs-12-en-23-oic acid methyl ester.

Compound 2 was isolated as a white powder and showed a molecular formula of C_32_H_52_O_7_ by HR-ESI-MS (m/z 571.3605, [M+Na]^+^, calcd. 571.3611). Its ^1^H and ^13^C NMR data (Table 1) closely resembled those of compound 1 except for the presence of two hydroxy groups at C-21 (δ_C_ 73.5) and C-22 (δ_C_ 78.7), respectively, as well as a relative difference in the configuration of the methyl and methyl ester attached to C-4. The positions of the two additional hydroxy groups were confirmed by the HMBC correlations (Figure 2) from H-21 (*δ*_H_ 3.49) to C-20 (*δ*_C_ 39.1), C-22 (*δ*_C_ 78.7), and C-29 (*δ*_C_ 16.8) and from H-22 (*δ*_H_ 3.40) and C-21 (*δ*_C_ 73.5), C-17 (*δ*_C_ 38.3), and C-21 (*δ*_C_ 73.5). Cross-peaks (Figure 2) between resonances at *δ*_H_ 3.49 (H-21), *δ*_H_ 1.06 (H_3_-30), and *δ*_H_ 1.51 (H-19) in the NOESY spectrum confirmed the *β*-orientation of the hydroxy group at C-21. A small coupling constant of 2.7 Hz between H-21 and H-22 suggested that two protons were in the *cis*-form and that the 22-OH should be *β*-oriented. Observation of NOESY correlations of H_3_-23 (*δ*_H_ 1.42) with H-3 (*δ*_H_ 3.11) and H-5 (*δ*_H_ 0.95) indicated the *α*-configuration of the methyl group at C-4. Therefore, compound **2** was identified as 11α-methoxy-3β,12,21β,22β-tetrahydroxyurs-12-en-24-oic acid methyl ester.

Compound **3** was obtained as a pale powder and displayed a molecular formula of C_32_H_50_O_7_ (*m*/*z* 569.3452, [M+Na]^+^, calcd. 569.3454) based on the HR-ESI-MS data. The ^1^H and ^13^C NMR data (Table 1) were very similar to compound **2** except for the absence of the hydroxy group at C-22, replaced by the carbonyl group, which was confirmed by the HMBC correlation (Figure 2) of H-28 (*δ*_H_ 1.09) to C-22 (*δ*_C_ 215.3). Therefore, compound **3** was established as 11α-methoxy-3β,12,21β-trihydroxy-22-oxours-12-en-24-oic acid methyl ester.

Compound **4** was obtained as a white powder. The molecular formula was identified as C_31_H_48_O_7_ by HR-ESI-MS (*m*/*z* 555.3298, [M+Na]^+^, calcd. 555.3298). The NMR spectroscopic data (Table 1) of **4** closely resembled those of **3** and differed only in the substitution at the C-11 position, where a hydroxyl group attached at C-11 in **4** instead of a methoxyl group in **3**. It was confirmed by the HMBC correlations (Figure 2) of H-11 (*δ*_H_ 4.16) with C-9 (*δ*_C_ 53.3) and C-12 (*δ*_C_ 145.9). The NOESY (Figure 2) cross-peaks of H-11 (*δ*_H_ 4.16) with H-25 (*δ*_H_ 0.98) and H-26 (*δ*_H_ 1.10) indicated that the hydroxy group at C-11 was an *α*-configuration. As a result, compound **4** was elucidated as 3β,11α,12,21β-tetrahydroxy-22-oxours-12-en-24-oic acid methyl ester.

**Figure 3:**
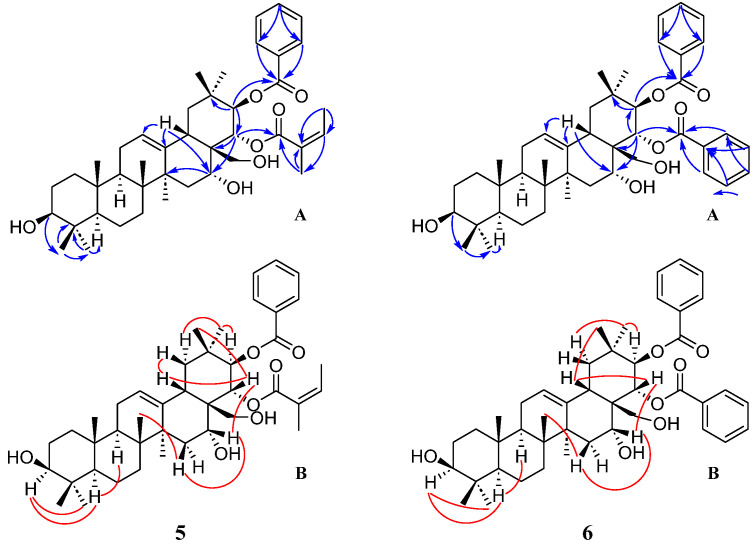
Key HMBC (A) and NOESY (B) correlations of compounds **5** and **6**.

Compound **5** was isolated as a white powder. Its molecular formula was determined as C_42_H_60_O_7_ based on HR-ESI-MS results (*m*/*z* 699.4236, [M+Na]^+^, calcd. 699.4237), indicating 13 degrees of unsaturation. Also, the Liebermann–Burchard reaction for **5** was positive, suggesting a triterpenoid. The NMR data (Table 2) displayed the presence of eight singlets of the methyl group (at *δ*_H_ 0.79, 0.91, 0.94, 0.96, 1.00, 1.18, 1.47, and 1.67), a distinctive proton at *δ*_H_ 2.77 (*dd*, *J* = 14.3, 4.2 Hz, H-18), and two characteristic unsaturated carbons at *δ*_C_ 124.0 (C-12) and 140.6 (C-13), revealing an oleanane-type skeleton. In addition, the HSQC spectrum showed two oxymethine protons connected to the carbons C-3 (*δ*_C_ 78.9) and C-16 (*δ*_C_ 69.9), respectively, and two methylene protons attached to the carbon C-28 (*δ*_C_ 63.6) appeared as a doublet of doublets at *δ*_H_ 2.93/3.30 (each d, *J* = 11.5 Hz). Three hydroxy groups attached to C-3, C-16, and C-28, respectively, were confirmed by the HMBC correlations (Figure 3) of H-3 (*δ*_H_ 3.24) with C-24 (*δ*_C_ 15.6) and C-23 (*δ*_C_ 28.1), of H-15 (*δ*_H_ 1.37), H-18 (*δ*_H_ 2.77), and H-22 (*δ*_H_ 5.55) with C-16 (*δ*_C_ 69.9), and of H-22 (*δ*_H_ 5.55) with C-28 (*δ*_C_ 63.6) successively. A typical coupling constant of H-3 (*J* = 11.5 Hz) and the NOESY correlations (Figure 3) of H-3 with H-23 (*δ*_H_ 1.00) and H-5 (*δ*_H_ 0.76) indicated the axial orientation of H-3 and *β*-configuration of OH-3. The NEOSY correlations of H-15β (*δ*_H_ 1.37) with H-26 (*δ*_H_ 0.91) and H-16 (*δ*_H_ 3.97) indicated that the hydroxy group at C-16 was *α*-configuration. Moreover, the ^1^H and ^13^C NMR spectra showed five aromatic protons at *δ*_H_ 7.98 (H-2′ and H-6′, d, *J* = 7.7 Hz), 7.53 (H-4′, t, *J* = 7.3 Hz), and 7.41 (H-3′ and H-5′, dd, *J* = 7.7, 7.3 Hz), six aromatic carbons at *δ*_C_ 128.3 (C-3′ and C-5′), 129.5 (C-2′ and C-6′), 130.3 (C-1′), and 132.3 (C-4′), and an ester carbonyl at *δ*_C_ 166.3 (C-7′). These data and HMBC correlations, together with the NOESY correlations of H-21 (*δ*_H_ 6.01) with H_3_-29 (*δ*_H_ 0.96) and H-19*α* (*δ*_H_ 2.62), confirmed the presence of a β-oriented benzoyloxy at C-21. Meanwhile, the NMR spectra also showed another ester carbonyl at *δ*_C_ 169.3 (C-1″), two olefinic carbons at 126.9 (C-2″) and 139.7 (C-3″), and two methyl groups at 15.6 (C-4″) and 20.3 (C-5″). These NMR data and NOESY correlations of H-22 (*δ*_H_ 5.55) with H-18 (*δ*_H_ 2.77) and H_3_-30 (*δ*_H_ 1.18), together with HMBC correlation results demonstrated an *α*-configuration of the angeloyloxy group at C-22 [36]. Hence, compound **5** was identified as 21β-benzoyloxy-22α-angeloylolean-12-ene-3β,16α,28-triol.

Compound **6** was obtained as a white amorphous powder and possessed a molecular formula of C_44_H_58_O_7_ based on HR-ESI-MS analysis (*m*/*z* 721.4092 [M+Na]^+^, calcd. 721.4080). The NMR data of **6** (Table 2) were similar to those of **5**. The inspection of the NMR data showed a benzoyloxy substituent at C-22 in compound **6** instead of an angeloyloxy group at C-22 in **5**. The cross-peaks (Figure 3) of H-22 (*δ*_H_ 5.66) with H_3_-30 (*δ*_H_ 1.18) and H-18 (*δ*_H_ 2.82) in the NOESY spectrum confirmed the *β*-orientation of the benzoyloxy group. Thus, compound **6** was ascertained as 21*β*,22*α*-dibenzoyloxyolean-12-ene-3*β*,16*α*,28-triol. This structure was previously reported but no NMR spectroscopic data were available before [37]. This work reports its NMR data for the first time.

**Figure 4:**
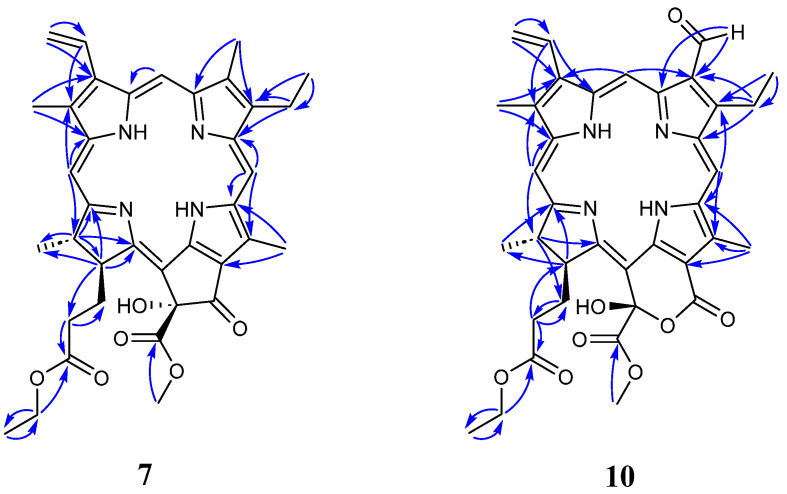
Key HMBC correlations of compounds **7** and **10**.

Compound **7** was obtained as a dark green amorphous solid with a molecular formula C_37_H_40_N_4_O_6_ measured by HR-ESI-MS (*m*/*z* 637.3029, [M+H]^+^, calcd. for C_37_H_41_N_4_O_6_, 637.3026). The ^1^H NMR spectrum (Table 3) of **7** indicated the presence of seven methyl groups at *δ*_H_ 1.12 (CH_3_-17^5^, t, *J* = 7.1 Hz), 1.69 (CH_3_-8^2^, t, *J* = 7.7 Hz), 1.69 (CH_3_-18^1^, d, *J* = 7.3 Hz), 3.23 (CH_3_-7^1^,s), 3.42 (CH_3_-2^1^, s), 3.65 (CH_3_-13^4^, s), and 3.71 (CH_3_-12^1^, s), and three olefinic protons at *δ*_H_ 9.42 (H-5, s), 9.57 (H-10, s), and 8.61 (H-20, s), respectively. A monosubstituted vinyl proton signal appeared at *δ*_H_ 6.18 (dd, *J* = 11.5, 1.2 Hz), 6.29 (dd, *J* = 17.8, 1.2 Hz), and 7.99 (dd, *J* = 17.8, 11.5 Hz). The peaks of two interchangeable hydrogens showed at *δ*_H_ 0.42 and −1.73 (both br s), which disappeared with the addition of D_2_O to the sample. The ^13^C NMR (Table 4) and DEPT spectra displayed two methylene carbons at *δ*_C_ 30.2 (C-17^1^) and 31.2 (C-17^2^), an oxymethylene carbon at *δ*_C_ 60.4 (C-17^4^), a methyl carbon at *δ*_C_ 14.1 (C-17^5^), and an ester carbonyl carbon at *δ*_C_ 173.0 (C-17^3^). An ethyl propanoate moiety attached to C-17 (*δ*_C_ 50.2) was confirmed by the analysis of the ^1^H-^1^H COSY spectrum and HMBC correlation (Figure 4). In addition, a carboxylic acid methyl ester unit and a hydroxyl group connected to C-13^2^ (*δ*_C_ 89.1) were also confirmed based on the NMR data analyses. A *J*_H-17-H-18_ value of 8.5 Hz suggested the *trans*-orientation of the CH_3_-18^1^ and ethyl propanoate units in the D ring. The *R*-configuration of the carboxylic acid methyl ester unit was assigned to C-13^2^ based on the downfield signal at *δ*_H_ 4.69 (H-17), in contrast with *δ*_H_ 4.16 ppm for the *S*-configuration, as concluded by Nakatani [38], which proposed an *α*-oriented hydroxyl group at C-13^2^. Consequently, the more downfield shift of H-17 caused by the deshielding effect of the hydroxyl group also demonstrated that H-17 and HO-13^2^ lay on the same side of the ring, so the relative configuration of CH_3_-18 was established as α-orientation and that of the ethyl propanoate moiety at C-17 as β-orientation. Thus, compound **7** was identified as 13^2^(*R*)-hydroxypheophorbide-a ethyl ester.

Compound **8** was isolated as a dark green amorphous solid and showed a molecular formula of C_37_H_38_N_4_O_7_ based on HR-ESI-MS analysis (*m*/*z* 673.2635, [M+Na]^+^, calcd. 673.2638). ^1^H and ^13^C NMR data (Table 3 and Table 4) for **8** were very similar to those of compound **7**. Compared with **7**, compound **8** had an aldehyde group at position C-7 instead of a methyl group in **7**, confirmed by the HMBC correlations of H-7^1^ (*δ*_H_ 11.11) with C-7 (*δ*_C_ 133.0) and C-6 (*δ*_C_ 151.2). Therefore, compound **8** was established as 7-formyl-13^2^-hydroxypheophorbide-a ethyl ester.

Compound **9** was obtained as a dark green powder with the molecular formula of (C_38_H_42_N_4_O_6_) deduced by HR-ESI-MS analysis (*m*/*z* 651.3179, [M+H]^+^, calcd. 651.3183). Its ^1^H and ^13^C NMR data (Table 3 and Table 4) were similar to those of compound **7**, the main distinction being the substituent groups and their configuration at position C-13^2^. The relative up-field shift of H-17 at *δ*_H_ 4.17, as mentioned in the structural elucidation of **7**, indicated the OH-13^2^ as β-configuration. Also, an ethyl formate unit with α-orientation was assigned to C-13^2^ based on the NMR data analyses. Thus, compound **9** was designated to be 13^3^-ethoxypheophorbide-a ethyl ester.

Compound **10** was obtained as a dark green powder and had a molecular formula of C_37_H_38_N_4_O_8_ as determined by its HR-ESI-MS results (*m*/*z* 667.2759, [M+H]^+^, calcd. 667.2768). Compounds **10** and **8** showed similar ^1^H and ^13^C NMR data (Table 3 and Table 4) and 2D NMR data. The main differences between **10** and **8** were observed from the chemical shift changes at C-13 (*δ*_C_ 112.3 for **10** vs. *δ*_C_ 127.1 for **8**) and C-13^1^ (*δ*_C_ 160.5 for **10** vs. *δ*_C_ 192.0 for **8**), indicating that compound **10** should have a six-membered lactone ring in its skeleton structure. Furthermore, the downfield shift of H-17 (*δ*_H_ 4.67), which arose from the inductive effect of OH-15^1^, suggested that OH-15^1^ was *β*-oriented. Thus, compound **10** was designated as 7-formyl-15^1^-hydroxypurpurin-7-lactone ethyl methyl diester.

The compounds **1**–**6** isolated from the leaves of *C. ptilosperma* in the present study were tested in terms of their cytotoxicity against six human cancer lines, namely Hela, MCF-7, BEL-7402, A549, HepG2, and MB-231, by MTT assay (Table 5). Compound **2** showed potent cytotoxicity toward HepG2 cells with an IC_50_ value of 2.57 ± 0.29 μM. In particular, the inhibitory effect of compound **2** was comparable to that of the positive control drug. Compounds **4** and **5** exhibited moderate cytotoxicity against MDA-MB-231 cells, with IC_50_ values of 11.31 ± 3.05 and 5.52 ± 0.13 μM, respectively. All the compounds were found to exhibit lower or no inhibitory activity against Hela, MCF-7, BEL-7402, and A549 cancer cells. The results indicated that triterpenoids were highly selective in inhibiting tumor cells.

Preliminary SAR (structure–activity relationship) analysis indicated that ursane-type triterpenes were more cytotoxic than oleanane-type triterpenes, and the presence of benzoyloxy and angeloyloxy groups reduced cytotoxicity against these cancer cells. Overall, among the isolated ursane-type compounds, the presence or absence of methoxy or hydroxyl groups at the position of C-11 and hydroxyl or carbonyl groups at the position of C-22 has no significant effect on cytotoxicity. Compared to the reported triterpenoids isolated from *camellias* [39,40,41,42], the triterpenoids isolated from *C. ptilosperma* in this study showed more potent cytotoxicity against hepatocellular carcinoma and lung cancer cells.

The cytotoxicity for compounds **7**–**10** in the absence of direct illumination was assayed against six tumor cell lines, Hela, MCF-7, BEL-7402, A549, HepG2, and MB-231, by the MTT method (Table 6). Compounds **8** and **9** exhibited limited or negligible cytotoxic activity against all tested cell lines. In contrast, compound **7** exhibited moderate inhibitory activity against MCF-7 cells, yielding an IC_50_ value of 5.26 ± 0.71 μM, while compound **10** demonstrated moderate cytotoxicity against BEL-7402 and HepG2 cells, with IC_50_ values of 7.68 ± 1.87 and 3.77 ± 0.49 μM, respectively. The findings for compounds **7** and **10**, observed in the absence of direct illumination, indicate that the cytotoxicity of pheophorbide-related compounds may involve mechanisms distinct from the previously reported photodynamic action.

**Figure 5:**
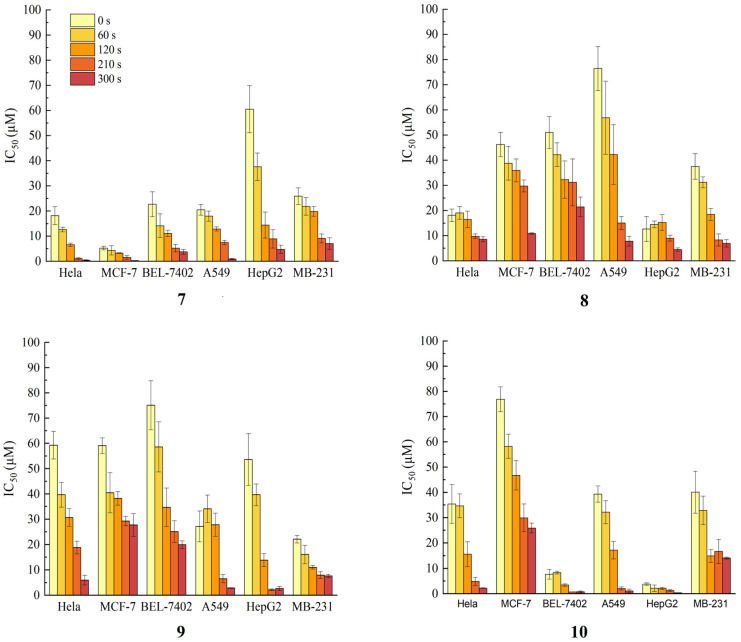
Cytotoxicity for Hela, MCF-7, BEL-7402, A549, HepG2, and MDA-MB-231 cells exposed to compounds **7**–**10** under photo-irradiation.

Furthermore, the photocytotoxicity of compounds **7**–**10** was assessed against the above tumor cell lines when exposed to light radiation (Figure 5). Four compounds demonstrated heightened inhibition of proliferation in all tested cell lines when subjected to illumination, and this effect intensified with longer light exposure times. Notably, compound **7** exhibited exceptional photocytotoxicity against Hela, MCF-7, and A549 cells, with IC_50_ values of 0.43 ± 0.15 μM, 0.28 ± 0.05 μM, and 0.92 ± 0.21 μM, respectively. Compound **10** demonstrated significant photodynamic cytotoxic activity against BEL-7402 and HepG2 cells with IC_50_ values of 0.77 ± 0.34 μM and 0.33 ± 0.04 μM, respectively. Conversely, compounds **8** and **9** exhibited limited photodynamic activity across all tested cell lines, despite a noticeable improvement in inhibition effects under light radiation. These results suggest that certain pheophorbide-related compounds derived from *C. ptilosperma* have the potential to serve as potent photosensitizers for photodynamic therapy (PDT).

Pheophorbide is one of the classes of porphyrins. Porphyrins are a family of heteromeric macrocyclic organic compounds containing four pyrrole rings linked by naturally occurring methine bridges, capable of generating monoclinic oxygen in the presence of light and oxygen, effectively killing tumor cells. [43,44,45]. Both natural and synthetic porphyrins exhibit photocytotoxicity. Thomas et al. [46] designed and prepared an N-fused porphyrin (NCP), displaying an IC_50_ value of 6 µM. Hynek et al. [47] synthesized porphyrin derivatives containing methyl, isopropyl, and phenyl groups, and strong photocytotoxicity against Hela cells with an IC_50_ value of 0.45 µM was shown. However, the porphyrins isolated in this study were less effective in inhibiting tumor cells than other types of photocytotoxic compounds, such as metal complexes [48,49,50]. This also suggested that compounds **7**–**10** can be further structurally modified to increase their photocytotoxicity. Preliminary SAR (structure–activity relationship) analysis suggested that the length of side chain substituents, differences in functional groups, and chiral carbon configurations did not reflect a significant degree of photocytotoxicity.

Two Gram-positive bacteria (*S. aureus* and *E. coli*) and two Gram-negative bacteria (*K. pneumoniae* and *P. aeruginosa*) were selected to evaluate the antibacterial activity of compounds **7**–**10** using MIC values. In the absence of light exposure, compounds **7**–**10** exhibited no activity against the four bacteria at a concentration of 100 μM. However, when the bacteria were exposed to compounds **7**–**10** with 30 min of photo-irradiation, four of the pheophorbides displayed limited antibacterial activity against *S. aureus* and *E. coli* (Table 7). Notably, compounds **8** and **10** exhibited sensitivity to *E. coli* and demonstrated a photodynamic antibacterial effect, with a MIC value of 0.625 μM. Conversely, none of the tested compounds displayed any activity for the two Gram-negative bacteria, whether subjected to photo-irradiation or not.

## 3. Experimental

### 3.1. General Experimental Procedures

HR-ESI-MS was measured on a Waters G2-XS Q-TOF mass spectrometer. All mass spectrometric data were obtained in positive ion mode using an ESI ion source, with a scan range from 100 to 1000 (*m*/*z*). NMR spectra were recorded on a Bruker AVANCE III HD 600 MHz spectrometer with TMS as the internal standard. Analytical HPLC was carried out on an SSI 1500 HPLC system equipped with a Model 201 UV detector and a Welch XB-C18 column (5 μm, 4.6 × 250 mm, 1.0 mL/min). Semipreparative HPLC was performed on a Laballiance HPLC system with a Welch XB-C18 column (5 μm, 10 × 250 mm, 4 mL/min) and a Model 500 UV detector. Silica gel (100–200 and 300–400 mesh, Qingdao Marine Chemical Inc., Qingdao, China), neutral alumina (100–200 mesh, Sinopharm Chemical Reagent Co., Ltd., Shanghai, China), and Sephadex LH-20 (20–150 μm, GE Healthcare, Boston, TX, USA) were used for column chromatography. Thin-layer chromatography (TLC) was performed on precoated silica gel G plates (Qingdao Marine Chemical Inc., Qingdao, China) and detected by heating after spraying a solution of 5% H_2_SO_4_ in EtOH. Photodynamic cytotoxicity and antibacterial activity evaluation used a 10 W halogen tungsten lamp (Philips, Amsterdam, The Netherlands) as the light source. All reagents used in the extraction and column chromatography process were analytically pure and in the HPLC analysis and preparation were of chromatographic purity.

Hela, MCF-7, BEL-7402, A549, HepG2, and MDA-MB-231 cancer cells were provided by the Chinese Cell Resource Center (National Infrastructure of Cell-Line Resources, Shanghai, China). Penicillin–streptomycin solution (100X, Beyotime Biotechnology, Beijing, China), fetal bovine serum (Gibco, Thornton, Australia), RPMI-1640, and DMEM cell culture media (Gibco, Beijing, China) were used for cell culture. The absorption values were recorded on a Synergy LX microplate reader. Tetrazolium bromide (MTT, Beyotime Biotechnology, Beijing, China) was used for cell colorimetry staining. *Staphylococcus aureus*, *Escherichia coli*, *Pseudomonas aeruginosa,* and *Klebsiella pneumoniae* were provided by the China Center for Type Culture Collection (CCTCC, Wuhan University, Wuhan, China).

### 3.2. Plant Material

The leaves of *C. ptilosperma* were picked in Daxin County, Chongzuo City, Guangxi Zhuang Region (China) in August 2019. A voucher specimen (No. 20190804-7014) representing this plant has been deposited at the Guangxi Institute of Botany, Chinese Academy of Sciences.

### 3.3. Extraction and Isolation

The dried leaves of *C. ptilosperma* (5.3 kg) were extracted three times with 95% EtOH (12 L each) at room temperature, and the combined solvent was evaporated in vacuo. The EtOH extract (1.4 kg) was suspended in water and successively partitioned with *n*-hexane and EtOAc three times (3 L each). Of these partitions, 412 g of EtOAc extract was subjected to a silica gel column and eluted with gradient mixtures of CH_2_Cl_2_-MeOH (from 200:1 to 5:1, *v*/*v*). Eluents were pooled based on TLC analysis to yield 12 combined fractions (Fr. 1-12). Further, Fr. 3 was chromatographed over a 100–200 mesh Al_2_O_3_ column and eluted with gradient mixtures of PE-EtOAc (20:1 to 8:1, *v*/*v*), and the eluents were pooled after TLC analysis to obtain six subfractions (Fr. 3.1–3.6). Fr. 3.1 was further separated on the LH-20 column and eluted with MeOH-CH_2_Cl_2_ (1:1, *v*/*v*) to yield three subfractions (Fr. 3.1.1–3.1.3.). Fr. 3.1.1 was purified by separation over a semi-preparative column, eluted with CH_3_CN-H_2_O (60:40, v/v) to yield compound **1** (*t*_R_ = 19.56 min, 7.0 mg). Fr. 3.1.2 was chromatographed on a semi-preparative, eluted with CH_3_CN-H_2_O (43:57 *v*/*v*) to give compound **2** (*t*_R_ = 23.33 min, 14.8 mg). Fr. 3.1.3 was subjected to a silica gel column (300–400 mesh) and eluted with a gradient of CH_2_Cl_2_-MeOH (40:1 to 10:1, *v*/*v*) to achieve three subfractions (Fr. 3.1.3.1–3.1.3.3). Fr. 3.1.3.1 was purified on a semi-preparative column, eluted with CH_3_CN-H_2_O (50:50 *v*/*v*) to give compounds **3** (*t*_R_ = 5.61 min, 6.8 mg) and **4** (*t*_R_ = 15.65 min, 3.5 mg). Fr. 3.1.3.2 was purified on a semi-preparative column with an elution of CH_3_CN-H_2_O (35:65 *v*/*v*) to obtain compounds **5** (*t*_R_ = 5.05 min, 13.7 mg) and **6** (*t*_R_ = 10.19 min, 3.0 mg).

Fr.1 was chromatographed on 300–400 mesh silica gel and eluted with PE/CH_2_Cl_2_ (40:1 and 20:1, *v*/*v*) to yield three fractions (Fr. 1.1 to Fr. 1.3). Fr. 1.1 was subjected to separation on the Sephadex LH-20 and eluted with CH_2_Cl_2_/MeOH (3:1, *v*/*v*) to yield a black eluate. This residue was purified on a semipreparative HPLC with acetonitrile as the mobile phase to obtain a mixture of 7 and 9, and the mixture was eluted with 90% acetonitrile/10% MeOH to yield 7 (25.7 mg, *t*_R_ = 10.8 min) and 9 (16.9 mg, *t*_R_ = 11.1 min). Fr. 1.2 was isolated on the Sephadex LH-20 with the elution of CH_2_Cl_2_/MeOH (1:1, *v*/*v*) to give 8 (51.9 mg). Fr. 1.3 was purified using the Sephadex LH-20 and eluted with CH_2_Cl_2_/MeOH (1:1, *v*/*v*) to give 10 (10.0 mg).

#### 3.3.1. Compound **1**

Colorless solid; [*α*]^20^_D_ + 9.08 (*c* 0.5, CH_2_Cl_2_); UV (CH_2_Cl_2_) *λ*_max_ (log*ε*) 201 (1.16) nm; HR-ESI-MS *m*/*z* 539.3718 [M+Na]^+^, calcd. for C_32_H_52_O_5_Na, 539.3712; ^1^H NMR (600 MHz) and ^13^C NMR (151 MHz) in CDCl_3_ (see Table 1).

#### 3.3.2. Compound **2**

White powder; [*α*]^20^_D_ + 4.36 (*c* 0.5, CH_2_Cl_2_); UV (CH_2_Cl_2_) *λ*_max_ (log*ε*) 199 (1.32) nm; HR-ESI-MS *m*/*z* 571.3605 [M+Na]^+^, calcd. for C_32_H_52_O_7_Na, 571.3611; ^1^H NMR (600 MHz) and ^13^C NMR (151 MHz) in CDCl_3_ (see Table 1).

#### 3.3.3. Compound **3**

Pale powder; [*α*]^20^_D_ + 3.01 (*c* 0.5, CH_2_Cl_2_); UV (CH_2_Cl_2_) *λ*_max_ (log*ε*) 201 (0.94) nm; HR-ESI-MS *m*/*z* 569.3452 [M+Na]^+^, calcd. for C_32_H_50_O_7_Na, 569.3454; ^1^H NMR (600 MHz) and ^13^C NMR (151 MHz) in CDCl_3_ (see Table 1).

#### 3.3.4. Compound **4**

White powder; [*α*]^20^_D_ + 15.27 (*c* 0.5, CH_2_Cl_2_); UV (CH_2_Cl_2_) *λ*_max_ (log*ε*) 192 (1.40) nm; HR-ESI-MS *m*/*z* 555.3298 [M+Na]^+^, calcd. for C_31_H_48_O_7_Na, 555.3298; ^1^H NMR (600 MHz) and ^13^C NMR (151 MHz) in CDCl_3_ (see Table 1).

#### 3.3.5. Compound **5**

White powder; [*α*]^20^_D_ − 4.21 (*c* 0.5, CH_2_Cl_2_); UV (CH_2_Cl_2_) *λ*_max_ (log*ε*) 225 (1.30), 262 (0.84), 279 (0.37) nm; HR-ESI-MS *m*/*z* 699.4236 [M+Na]^+^, calcd. for C_42_H_60_O_7_Na, 699.4237; ^1^H NMR (600 MHz) and ^13^C NMR (151 MHz) in CDCl_3_ (see Table 2).

#### 3.3.6. Compound **6**

White powder; [*α*]^20^_D_ + 2.90 (*c* 0.5, CH_2_Cl_2_); UV (CH_2_Cl_2_) *λ*_max_ (log*ε*) 230 (1.11), 275 (0.52), 281 (0.29) nm; HR-ESI-MS *m*/*z* 721.4092 [M+Na]^+^, calcd. for C_44_H_58_O_7_Na, 721.4080; ^1^H NMR (600 MHz) and ^13^C NMR (151 MHz) in CDCl_3_ (see Table 2).

#### 3.3.7. Compound **7**

Dark green amorphous solid; [*α*]^20^_D_ + 0.42 (*c* 0.2, CH_2_Cl_2_); UV (CH_2_Cl_2_) *λ*max (log *ε*) 250 (1.06), 410 (2.80), 422 (2.93), 511 (0.40), 539 (0.27), 615 (0.27), 670 (1.53) nm; IR (ATR) *v*_max_ 3329, 3110, 1759, 1620, 1249; HR-ESI-MS *m*/*z* 637.3029 [M+H]^+^, calcd. for C_37_H_41_N_4_O_6_, 637.3026; ^1^H NMR (600 MHz) and ^13^C NMR (151 MHz) in CDCl_3_ (see Table 3 and Table 4).

#### 3.3.8. Compound **8**

Dark green amorphous solid; [*α*]^20^_D_ + 0.67 (*c* 0.2, CH_2_Cl_2_); UV (CH_2_Cl_2_) *λ*max (log *ε*) 250 (1.22), 432 (2.71), 447 (2.90), 534 (0.36), 563 (0.23), 606 (0.21), 657 (0.92) nm; IR (ATR) *v*_max_ 3344, 3107, 2932, 1764, 1250; HR-ESI-MS *m*/*z* 673.2635 [M+Na]^+^, calcd. for C_37_H_60_N_4_O_7_Na, 673.2638; ^1^H NMR (600 MHz) and ^13^C NMR (151 MHz) in CDCl_3_ (see Table 3 and Table 4).

#### 3.3.9. Compound **9**

Dark green powder; [*α*]^20^_D_ + 0.09 (*c* 0.2, CH_2_Cl_2_); UV (CH_2_Cl_2_) *λ*max (log *ε*) 252 (1.03), 420 (2.65), 439 (2.86), 520 (3.60), 552 (0.20), 609 (0.28), 662 (6.81) nm; IR (ATR) *v*_max_ 3320, 3123, 1742, 1230; HR-ESI-MS *m*/*z* 651.3179 [M+H]^+^, calcd. for C_38_H_43_N_4_O_6_, 651.3183; ^1^H NMR (600 MHz) and ^13^C NMR (151 MHz) in CDCl_3_ (see Table 3 and Table 4).

#### 3.3.10. Compound **10**

Dark green powder; [*α*]^20^_D_ + 0.50 (*c* 0.2, CH_2_Cl_2_); UV (CH_2_Cl_2_) *λ*max (log *ε*) 247 (1.12), 428 (2.92), 433 (2.73), 525 (0.24), 566 (0.17), 606 (0.13), 662 (0.59) nm; IR (ATR) *v*_max_ 3358, 3109, 2920, 1777, 1238; HR-ESI-MS *m*/*z* 667.2759 [M+Na]^+^, calcd. for C_37_H_39_N_4_O_8_, 667.2768; ^1^H NMR (600 MHz) and ^13^C NMR (151 MHz) in CDCl_3_ (see Table 3 and Table 4).

### 3.4. Biological Assay

#### 3.4.1. Cytotoxicity Assays

The cytotoxic activity of compounds 1–6 against Hela, MCF-7, BEL-7402, A549, HepG2, and MDA-MB-231 cancer cell lines was evaluated using the MTT assay according to the reported method [51,52] with doxorubicin as a positive control drug. MCF-7 and BEL-7402 cells were cultured in RPMI-1640 medium containing 10% fetal bovine serum, and Hela, A549, HepG2, and MDA-MB-231 cells were cultured in DMEM medium containing 10% fetal bovine serum, all of which were incubated in a constant temperature incubator at 5% CO_2_ at 37 °C. A549 in the logarithmic growth phase were inoculated into 96-well plates at a density of 4 × 10^4^ cells/mL. MCF-7, HepG2, BEL-7402, MDA-MB-231, and Hela were inoculated at a density of 5 × 10^4^ cells/mL, with 100 μL in each well. After the cells were attached to the wall, the drug treatment was performed. Different concentrations (0.1, 0.5, 1.0, 5.0, 1.0, and 20.0 μM) of doxorubicin (dissolved in PBS) and compounds **1**–**6** (1.0, 2.0, 5.0, 10.0, 25.0, and 50.0 μM, dissolved in 0.1% DMSO/PBS) were administered. Three parallel tests were conducted for each concentration and the cells were cultured for 48 h. An amount of 10 μL of MTT was added to each well, and the cells were incubated. After incubation for 4 h at 37 °C, the medium was aspirated and 150 μL of DMSO solution was added and shaken for 10 min, the plate was shocked using an enzyme marker, and the absorbance value was detected at 490 nm to calculate the cell survival rate. The entire experiment was repeated three times.

#### 3.4.2. Photocytotoxicity Assay

The cytotoxicity of compounds **7**–**10** with or without photo-irradiation was evaluated against the same six cancer cell lines by the MTT method. For the photodynamic cytotoxicity assay, a halogen tungsten lamp was employed as the irradiation source. The lamp was positioned immediately above the 96-well plate, maintaining a separation distance of 10 cm. The concentrations of the compounds **7**–**10** were set at 0.1, 0.5, 1.0, 5.0, 10.0, 50.0, and 100.0 μM. Immediately after the addition of different concentrations of these compounds, the cells were exposed to continuous light for 0, 60, 120, 210, and 300 s. The subsequent experimental steps were the same as described in Section 3.4.1.

#### 3.4.3. Photodynamic Antibacterial Activity Assay

The MIC (minimal inhibitory concentration) value was identified as the lowest concentration of the compound that inhibited visible bacterial growth following the incubation period. It was determined using the double dilution method. *S. aureus*, *E. coli*, *K. pneumoniae*, and *P. aeruginosa* were inoculated on Mueller–Hinton agar medium and incubated at 37 °C for 24 h. The concentration of the bacterial suspension was adjusted to 1.0 × 10^6^ CFU/mL by the use of sterile saline solution and then the bacterial solution was inoculated on a 96-well plate with 100 µL per-well.

Compounds **7**–**10** and positive control drugs were diluted to attain the final concentrations within the range of 0.625–10.0 μM. These different concentrations of solutions and positive control drugs were inoculated into 96-well plates with an inoculation volume of 50 µL per well, while for the blank control, only 50 µL of culture medium was added. The solutions were then incubated at 37 °C for 24 h. All the above operations were carried out under light-protected conditions. Bacterial growth in the treated group was determined by comparing the characteristics of bacterial growth in the blank control group and recording the minimum concentration corresponding to bacterial growth.

In the photodynamic antibacterial test, the samples were exposed to continuous light irradiation for a duration of 30 min using a halogen tungsten lamp positioned 10 cm above the samples after administration. The other procedures were the same as in the light avoidance condition. The experiment was repeated three times.

### 3.5. Statistical Analysis

Data from the cytotoxicity assays were evaluated according to their means and standard deviations. The cytotoxic concentration at 50% was determined to compare with the control obtained from nonlinear regression. These analyses were performed using SPSS^®^ Statistics 18.0 (IBM software, Armonk, NY, USA).

## 4. Conclusions

In summary, ten new compounds were isolated from the leaves of *Camellia ptilosperma*, including six triterpenes and four pheophorbides. The structures of the undescribed compounds were based on NMR and HR-ESI-MS spectroscopic data.

Meanwhile, the cytotoxic activity of the six triterpenes against six cancer cell lines was evaluated by MTT assay. Among them, compounds **1**, **4**, and **5** showed no significant cytotoxicity against any of the six cell lines. Compound **2** exhibited potent cytotoxicity against HepG2 cells with an IC_50_ value of 2.57 ± 0.29 μM, which was close to that of the positive control drug doxorubicin, indicating that compound **2** significantly inhibited HepG2 proliferation. Compound **5** also showed notable cytotoxicity against MDA-MB231 cells with an IC_50_ value of 5.52 ± 0.13 μM, which was stronger than that of the positive control drug doxorubicin. Compounds **7**–**10**, the four pheophorbides, did not exhibit more pronounced cytotoxicity and bacteriostatic activity in the absence of light. The cytotoxicity of all compounds increased significantly after exposure to light, in a manner shown to be time-dependent, and the IC_50_ values of compounds **7**–**10** were lower than that of the positive control drug for all cells after 300 s of irradiation. On the other hand, they displayed a certain degree of inhibitory effect against *S. aureus* and *E. coli* after 30 min of irradiation, and the MIC values of compounds **8** and **10** were lower than those of the positive control drug ampicillin. However, these compounds did not show any antibacterial activity against *P. aeruginosa* and *K. peneumoniae* in the presence or absence of light.

Therefore, compounds **2** and **5** are promising for the treatment of liver and breast tumors. Compounds **7**–**10** have potential as photosensitizers for the treatment of tumors and bacterial infections. It is hoped that our study can provide a new direction for the research on and application of *Camellia ptilosperma*. We look forward to investigating its exact mechanism of action in further studies.

## Figures and Tables

**Table 1:** ^1^H (600 MHz) and ^13^C (151MHz) NMR data for compounds **1**–**4** in CDCl_3_.

	1		2		3		4	
No.	*δ*_H_ (*J* in Hz)	*δ*_C_	*δ*_H_ (*J* in Hz)	*δ*_C_	*δ*_H_ (*J* in Hz)	*δ*_C_	*δ*_H_ (*J* in Hz)	*δ*_C_
1	1.32 m 2.27 dt-*like* (13.6, 3.4)	38.6	1.21 m 2.34 dt-*like* (13.4, 3.5)	39.8	1.22 m 2.38 dt-*like* (13.3, 3.3)	39.9	1.22 m 2.40 dt-*like* (13.3, 3.5)	41.8
2	1.60–1.71 m	26.8	1.77 m 2.03 m	28.3	1.78 m 2.04 m	28.4	1.79 m 2.04 m	28.2
3	4.00 dd (11.5, 5.1)	75.3	3.11 dd (12.1, 4.3)	77.9	3.11 ddd (11.9, 11.9, 4.4)	77.9	3.11 td-*like* (11.7, 4.3)	77.8
4		54.2		49.3		49.4		49.2
5	1.55 m	51.1	0.95 m	56.7	0.97 m	56.8	0.97 m	56.6
6	1.01 m 1.55 m	21.3	1.66 m 1.82 m	19.9	1.67 m 1.84 m	19.8	1.68 m 1.85 m	19.9
7	1.31 m 1.53 m	33.7	1.35–1.48 m	34.2	1.40–1.49 m	34.5	1.38–1.51 m	34.2
8		43.1		42.7		42.5		42.4
9	1.94 d (10.4)	46.3	1.86 d (10.7)	45.3	1.89 d (10.7)	44.7	1.61 d (9.8)	53.3
10		37.7		38.5		38.6		38.5
11	4.26 d (10.4)	76.4	4.23 d (10.5)	76.5	4.27 d (10.7)	76.3	4.16 br d (10.7)	70.5
12		141.7		142.8		143.4		145.9
13		118.3		116.6		114.5		112.3
14		40.6		41.0		40.6		40.8
15	0.98 br d (13.5) 1.77 td-*like* (13.5, 5.1)	27.1	1.02 m 1.81 m	26.4	1.13 m 1.79 m	25.6	1.13 m 1.76 m	25.3
16	0.83 br d (13.5) 2.01 td-*like* (13.5, 4.8)	27.5	1.02 m 1.81 m	26.6	1.26 m 2.06 m	28.0	1.26 m 2.05 m	28.1
17		33.3		38.3		47.8		47.7
18	2.24 dd (11.4, 1.3)	47.6	2.62 br d (11.2)	41.8	2.75 d (11.6)	48.1	2.66 br d (11.6)	47.7
19	1.37 m	40.8	1.51 m	39.1	1.95 m	38.5	1.95 m	38.4
20	1.03 m	39.5	1.45 m	39.1	1.29 m	47.2	1.30 m	47.3
21	1.26 m 1.41 m	31.2	3.49 dd (10.5, 2.7)	73.5	4.05 dd (11.6, 3.0)	76.4	4.06 dd (11.6, 3.0)	76.4
22	1.32–1.46 m	41.6	3.40 d (2.7)	78.7		215.3		215.2
23		178.1	1.42 s	23.8	1.43 s	23.8	1.44 s	23.8
24	1.18 s	11.0		178.4		178.3		178.3
25	1.13 s	16.6	0.95 s	14.0	0.97 s	14.0	0.98 s	14.3
26	1.09 s	18.0	1.12 s	18.0	1.12 s	18.1	1.10 s	18.0
27	1.21 s	24.0	1.19 s	24.1	1.29 s	23.6	1.28 s	23.8
28	0.80 s	28.5	0.96 s	21.9	1.09 s	20.1	1.09 s	20.3
29	0.92 d (6.6)	17.0	0.96 d (6.6)	16.8	1.02 d (6.7)	16.0	0.98 d (6.6)	15.8
30	0.93 d (6.4)	21.2	1.06 d (6.1)	16.0	1.20 d (6.3)	16.8	1.19 d (6.3)	16.7
3-OH					3.39 d (11.9)		3.39 d (12.0)	
12-OH	4.53 br s		4.69 br s		4.76 s		4.88 br s	
21-OH					3.79 d (3.0)		3.80 d (3.0)	
11-OCH_3_	3.18 s	51.3	3.14 s	51.1	3.14 s	50.8		
23-OCH_3_	3.73 s	52.2						
24-OCH_3_			3.69 s	51.3	3.70 s	51.3	3.71 s	51.4

**Table 2:** ^1^H (600 MHz) and ^13^C (151MHz) NMR data for compounds **5** and **6** in CDCl_3_.

	5		6	
No.	*δ*_H_ (*J* in Hz)	*δ*_C_	*δ*_H_ (*J* in Hz)	*δ*_C_
1	1.00 m 1.65 m	38.6	1.01 m 1.64 m	38.6
2	1.54–1.66 m	27.2	1.55–1.67 m	27.1
3	3.24 dd (11.5, 4.3)	78.9	3.23 dd (11.6, 4.4)	79.0
4		38.8		38.8
5	0.76 br d (11.8)	55.1	0.76 br d (10.9)	55.2
6	1.40 m 1.56 m	18.3	1.40 m 1.56 m	18.3
7	1.30 m 1.57 m	32.7	1.31 m 1.57 m	32.7
8		41.0		41.0
9	1.64 m	46.5	1.64 m	46.5
10		36.9		36.9
11	1.88–1.96 m	23.5	1.88–1.97 m	23.5
12	5.48 t (3.3)	124.8	5.50 t (3.3)	125.1
13		140.6		140.7
14		39.7		39.7
15	1.37 m 1.70 m	33.7	1.38 m 1.70 m	33.7
16	3.97 br s	69.9	4.02 br s	69.8
17		47.7		47.7
18	2.77 dd (14.3, 4.2)	39.2	2.82 dd (14.4, 4.7)	39.2
19	1.32 m 2.62 dd (14.3, 13.2)	46.4	1.32 m 2.66 dd (14.4, 13.9)	46.4
20		36.1		36.1
21	6.01 d (10.2)	78.7	6.19 d (10.2)	78.6
22	5.55 d (10.2)	73.1	5.66 d (10.2)	74.5
23	1.00 s	28.1	1.00 s	28.1
24	0.79 s	15.6	0.79 s	15.6
25	0.94 s	15.6	0.94 s	15.6
26	0.91 s	16.7	0.91 s	16.7
27	1.47 s	27.1	1.47 s	27.1
28	2.93 d (11.5) 3.30 d (11.5)	63.6	2.97 d (11.5) 3.32 d (11.5)	63.6
29	0.96 s	29.1	0.96 s	29.1
30	1.18 s	19.5	1.18 s	19.5
1′		130.3		130.1
2′, 6′	7.98 d (7.7)	129.5	7.91 d (7.8)	129.7
3′, 5′	7.41 dd (7.7, 7.3)	128.3	7.32 dd (7.8, 7.2)	128.4
4′	7.53 t (7.3)	132.8	7.46 t (7.2)	132.3
7′		166.3		168.3
1″		169.3		129.0
2″		126.9	7.88 d (7.8)	129.5
3″	5.91 br q (7.2)	139.7	7.33 dd (7.8, 7.2)	128.2
4″	1.77 br d (7.2)	15.6	7.42 t (7.2)	132.0
5″	1.67 br s	20.3	7.33 dd (7.8, 7.2)	128.2
6″			7.88 d (7.8)	129.5
7″				166.5

**Table 3:** ^1^H (600 MHz) NMR data for compounds **7**–**10** in CDCl_3_.

No.	7	8	9	10
2^1^	3.42, s	3.39, s	3.43, s	3.37, s
3^1^	7.99, dd (17.8, 11.5)	7.99, dd (17.8, 11.6)	8.03, dd (17.8, 11.6)	7.90, dd (17.8, 11.6)
3^2^	6.18, dd (11.5, 1.2) 6.29, dd (17.8, 1.2)	6.23, d, (11.6) 6.37, d, (17.8)	6.20, dd (11.6, 1.3) 6.31, dd (17.8, 1.3)	6.17, d (11.6) 6.35, d (17.8)
5	9.42, s	10.37, s	9.50, s	10.28, s
7^1^	3.23, s	11.11, s	3.28, s	11.00, s
8^1^	3.68, q (7.7)	4.01, m	3.73, q (7.7)	3.91, q (7.5)
8^2^	1.69, t (7.7)	1.80, t (7.7)	1.71, t (7.7)	1.75, t (7.5)
10	9.57, s	9.64, s	9.64, s	9.65, s
12^1^	3.71, s	3.69, s	3.74, s	3.83, s
13^4^	3.65, s	3.66, s	4.09, m 4.25, m	
13^5^			0.90, t (7.1)	
15^3^				3.79, s
17	4.69, br d (8.5)	4.67, br dd (8.5, 2.2)	4.17 br dd (9.2, 2.5)	4.09, br d (9.2)
17^1^	2.12, m 2.27, m	2.09, m 2.28, m	2.27, m 2.94, m	1.85, m 2.62, m
17^2^	2.04, m 2.44, m	2.09, m 2.46, m	2.25, m 2.52, m	2.27, m 2.51, m
17^4^	4.02, m	4.04, m	4.10, q (7.1)	3.95–4.09, m
17^5^	1.12, t (7.1)	1.14, t (7.1)	1.15, t (7.1)	1.11, t (7.1)
18	4.49, br q (7.3)	4.49, br q (7.3)	4.50, br q (7.3)	4.45, br q (7.1)
18^1^	1.69, d (7.3)	1.71, d (7.3)	1.60, d (7.3)	1.62, d (7.1)
20	8.61, s	8.59, s	8.65, s	8.64, s
13^2^-OH	5.35, br s	5.36, br s	5.51, br s	
15^1^-OH				6.34, br s
NH ^1^	−1.73, 0.42 (br s)	−1.62, 0.44 (br s)	−1.84, 0.26 (br s)	−1.17, −0.71 (br s)

^1^ Interchangeable proton.

**Table 4:** ^13^C (151 MHz) NMR data for compounds **7**–**10** in CDCl_3_.

No.	7	8	9	10
1	142.1	143.6	142.0	143.1
2	131.9	132.3	131.8	131.9
2^1^	12.1	12.1	12.1	12.0
3	136.3	137.9	136.3	137.8
3^1^	129.0	128.6	129.1	128.4
3^2^	122.9	123.7	122.9	123.6
4	136.4	137.0	136.2	137.0
5	97.9	102.0	98.0	103.8
6	155.6	151.2	155.3	151.4
7	136.5	133.0	136.5	132.9
7^1^	11.3	187.7	11.3	187.6
8	145.3	159.4	145.3	159.4
8^1^	19.5	19.1	19.5	19.1
8^2^	17.5	19.4	17.5	19.5
9	151.0	147.1	151.3	146.1
10	104.3	106.7	104.2	106.4
11	137.7	137.7	137.8	141.7
12	129.6	132.9	129.5	131.5
12^1^	12.3	12.5	12.3	12.5
13	126.3	127.1	127.0	112.3
13^1^	192.0	192.0	192.2	160.5
13^2^	89.1	89.0	88.9	
13^3^	173.5	173.1	172.3	
13^4^	53.8	53.9	62.8	
13^5^			14.0	
14	150.2	151.1	150.0	136.7
15	107.6	107.4	107.8	102.0
15^1^				100.5
15^2^				170.6
15^3^				54.3
16	161.9	164.7	162.4	169.0
17	50.2	50.4	51.8	53.9
17^1^	30.2	30.0	31.1	31.3
17^2^	31.2	31.3	31.6	32.2
17^3^	173.0	172.9	173.6	173.1
17^4^	60.4	60.5	60.5	60.5
17^5^	14.1	14.1	14.1	14.1
18	50.8	50.8	50.4	50.2
18^1^	22.7	22.7	22.8	22.2
19	172.8	174.6	172.4	173.3
20	93.4	93.7	93.6	94.1

**Table 5:** Cytotoxicity (IC_50_, μM ± SD, n = 3) of compounds **1**–**6** and doxorubicin against six cancer lines.

Compounds	1	2	3	4	5	6	Doxorubicin
Hela	>50	>50	>50	>50	>50	>50	5.19 ± 0.26
MCF-7	>50	22.18 ± 8.95	30.93 ± 5.10	19.62 ± 2.16	>50	>50	12.03 ± 1.15
BEL-7402	37.20 ± 5.46	>50	>50	>50	>50	20.04 ± 3.46	4.82 ± 0.76
A549	14.08 ± 1.16	>50	>50	48.02 ± 12.14	>50	>50	8.05 ± 1.12
HepG2	29.07 ± 5.69	2.57 ± 0.29	>50	42.63 ± 3.97	>50	>50	2.49 ± 0.36
MDA-MB231	>50	27.05 ± 7.18	19.09 ± 0.75	11.31 ± 3.05	5.52 ± 0.13	>50	7.96 ± 1.17

**Table 6:** Cytotoxicity (IC_50_, μM ± SD, n = 3) of compounds **7**–**10** against six cancer cell lines in darkness.

Compounds	7	8	9	10
Hela	18.19 ± 3.62	18.08 ± 2.48	59.26 ± 5.50	35.41 ± 7.62
MCF-7	5.26 ± 0.71	46.26 ± 4.81	59.08 ± 3.09	76.84 ± 4.93
BEL-7402	22.72 ± 4.98	51.04 ± 6.36	75.07 ± 9.73	7.68 ± 1.87
A549	20.48 ± 2.16	76.43 ± 8.75	27.13 ± 6.08	39.31 ± 3.23
HepG2	60.53 ± 9.40	12.65 ± 4.96	53.57 ± 10.28	3.77 ± 0.49
MDA-MB231	25.92 ± 3.30	37.50 ± 5.09	22.06 ± 1.51	40.06 ± 8.33

**Table 7:** MICs (μM, n = 3) of compounds **7**–**10** against four bacteria.

Compounds	7	8	9	10	Positive Control
*S. aureus*	2.5	5.0	1.25	2.5	1.25 (ampicillin)
*E. coli*	5.0	0.625	2.5	0.625	2.50 (ampicillin)
*P. aeruginosa*	>10	>10	5.0	>10	0.625 (ceftazidimea)
*K. peneumoniae*	>10	>10	>10	>10	1.25 (ceftazidimea)

## Data Availability

The data presented in this study are available in the Supplementary Materials.

## Supplementary Materials

The following supporting information can be downloaded at: https://www.mdpi.com/article/10.3390/molecules28207058/s1, Figure S1 (S1-1 to S1-8): Spectroscopic data for compound **1**; Figure S2 (S2-1 to S2-8): Spectroscopic data for compound **2**; Figure S3 (S3-1 to S3-8): Spectroscopic data for compound **3**; Figure S4 (S4-1 to S4-8): Spectroscopic data for compound **4**; Figure S5 (S5-1 to S5-8): Spectroscopic data for compound **5**; Figure S6 (S6-1 to S6-8): Spectroscopic data for compound **6**; Figure S7 (S7-1 to S7-7): Spectroscopic data for compound **7**; Figure S8 (S8-1 to S8-7): Spectroscopic data for compound **8**; Figure S9 (S9-1 to S9-7): Spectroscopic data for compound **9**; Figure S10 (S10-1 to S10-7): Spectroscopic data for compound **10**; Figure S11: The scheme of extraction and isolation.

## Sample Availability

Compounds were deposited at the Medical College of Guangxi University.
