# Supplementary material for: Triterpenes and Pheophorbides from Camellia ptilosperma and Their Cytotoxicity, Photocytotoxicity, and Photodynamic Antibacterial Activity
Source: Molecules. 2023 Oct 12;28(20):7058. doi: 10.3390/molecules28207058 (PMC10609551; doi:10.3390/molecules28207058)
Supplement: Supplementary file 1 [file molecules-28-07058-s001.zip › molecules-2649594-supplementary.pdf]

Supplementary Material

Triterpenes and Pheophorbides from *Camellia ptilosperma* and their Cytotoxicity, Photocytotoxicity, and Photodynamic Antibacterial Activity

Siyuan Ma [1 †], Mengling Weng [2 †], Ting Yang [3], Li Ge [4, *] and Kedi Yang [1, 4, *]

[1] School of Chemistry & Chemical Engineering, Guangxi University, Nanning 530004, China

[2] Key Laboratory of Sugarcane Biotechnology and Genetic Improvement (Guangxi), Ministry of Agricul-ture & Rural Affairs, Guangxi Key Laboratory of Sugarcane Genetic Improvement, Sugarcane Research Institute, Guangxi Academy of Agricultural Sciences, Nanning 530004, China

[3] Guangxi Fangcheng Golden Camellia National Nature Reserve Management Center, Fangchenggang 538021, China

[4] Medical College, Guangxi University, Nanning 530004, PR China

[*] Correspondences: geli_2009@163.com (L. Ge), kdyang@163.com (K.D. Yang); Tel.: +86-771-3949953 (K.D. Yang)

[†] These authors contributed equally to this work.

CONTENTS

1. Spectroscopic data for compound **1**..1

2. Spectroscopic data for compound **2**..5

3. Spectroscopic data for compound **3**..9

4. Spectroscopic data for compound **4**..13

5. Spectroscopic data for compound **5**..17

6. Spectroscopic data for compound **6**..21

7. Spectroscopic data for compound **7**..25

8. Spectroscopic data for compound **8**..29

9. Spectroscopic data for compound **9**..33

10. Spectroscopic data for compound **10**..37

11. The scheme of extraction and isolation...41

1. Spectroscopic data for compound 1

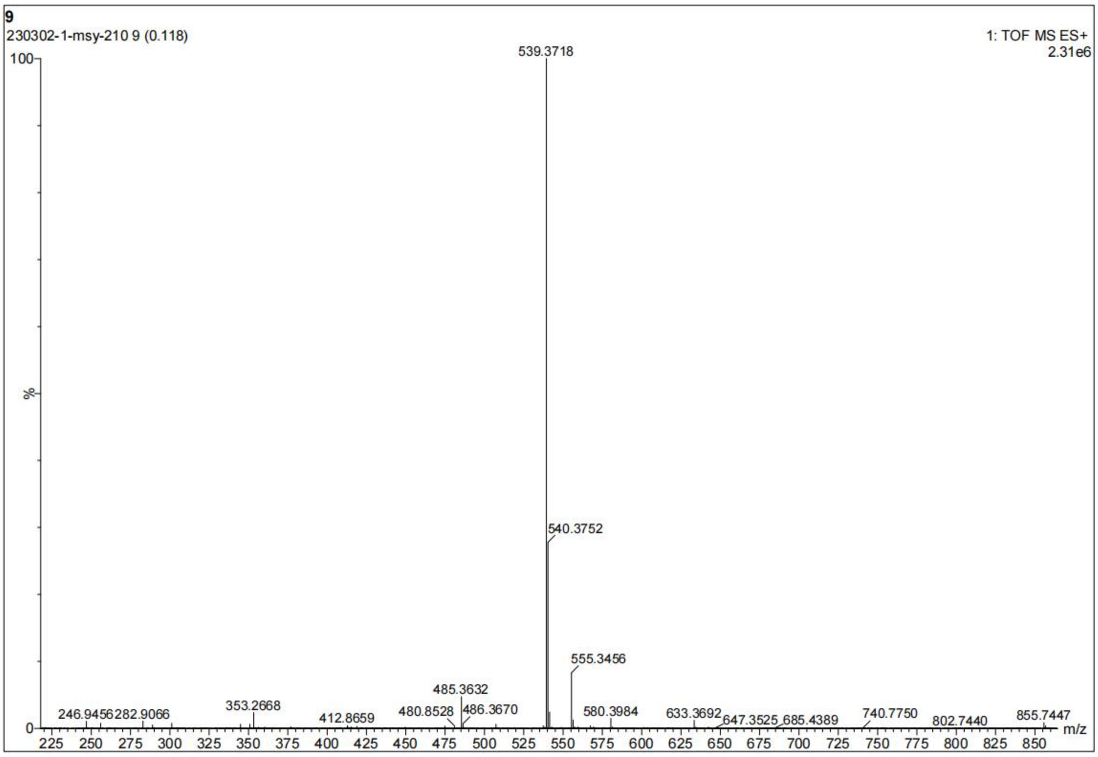

Figure S1-1. The HR-ESI-MS of compound **1**.

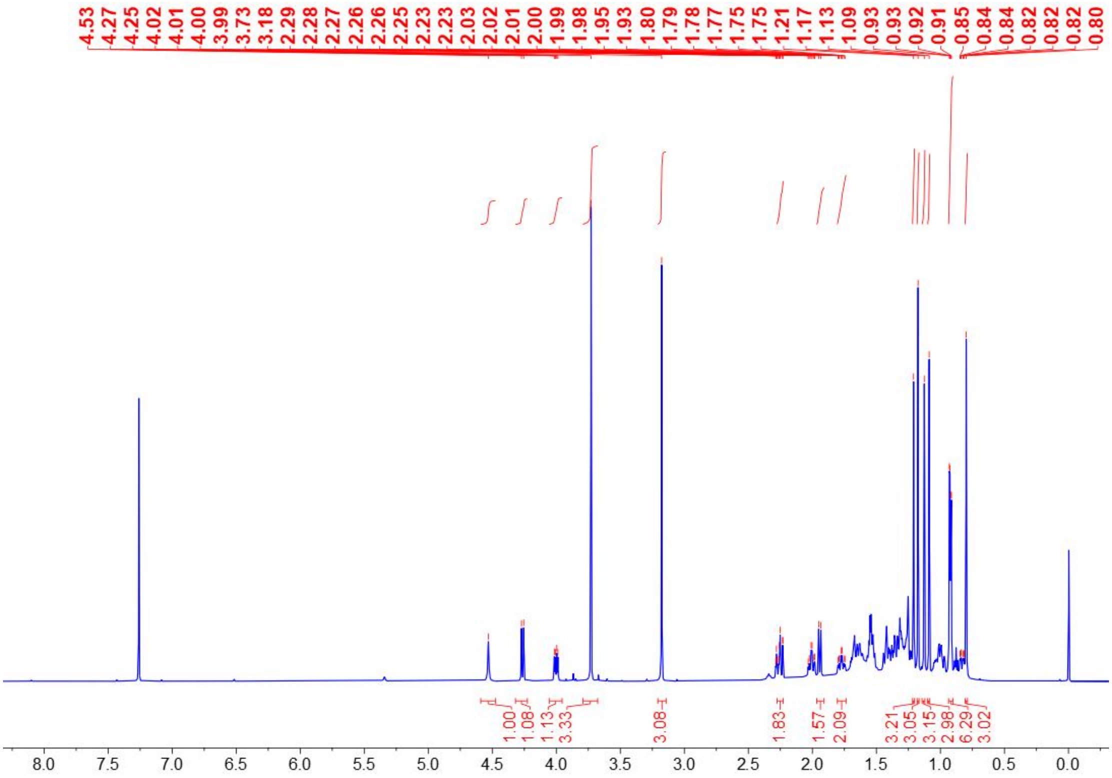

Figure S1-2. The ^{1}H NMR spectrum of compound **1** (600 MHz, $CDCl_3$).

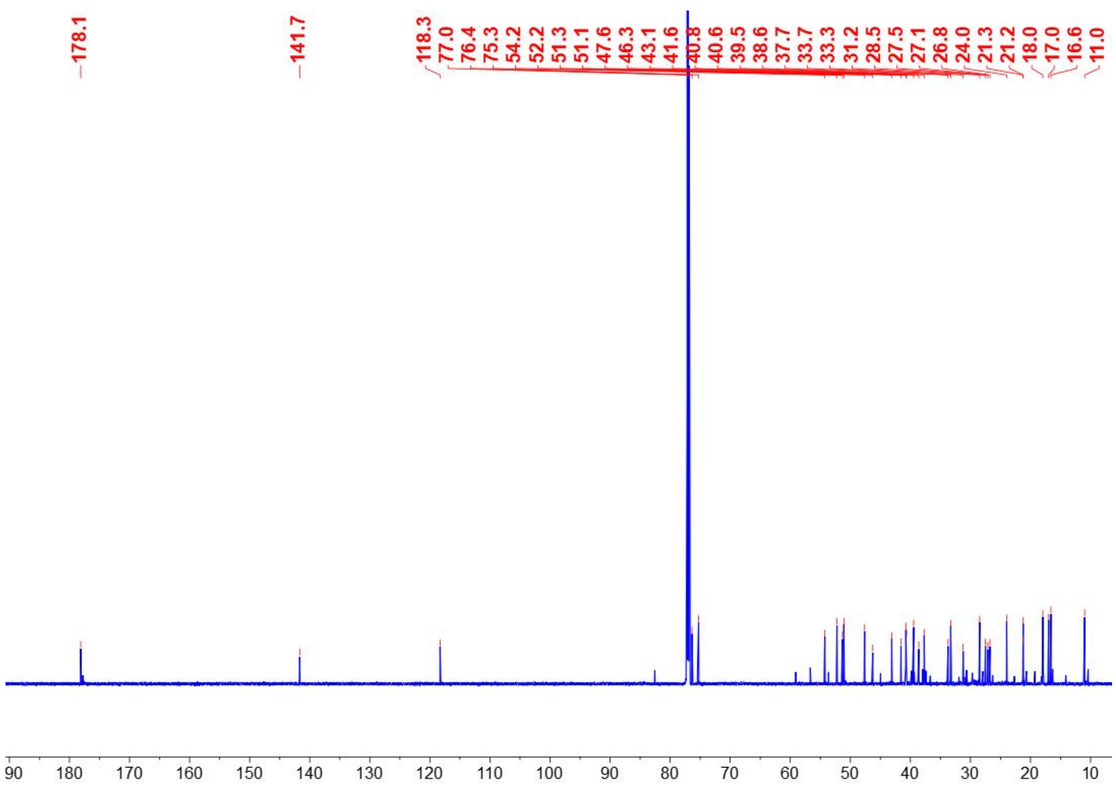

Figure S1-3. The ^{13}C NMR spectrum of compound **1** (151 MHz, $CDCl_3$).

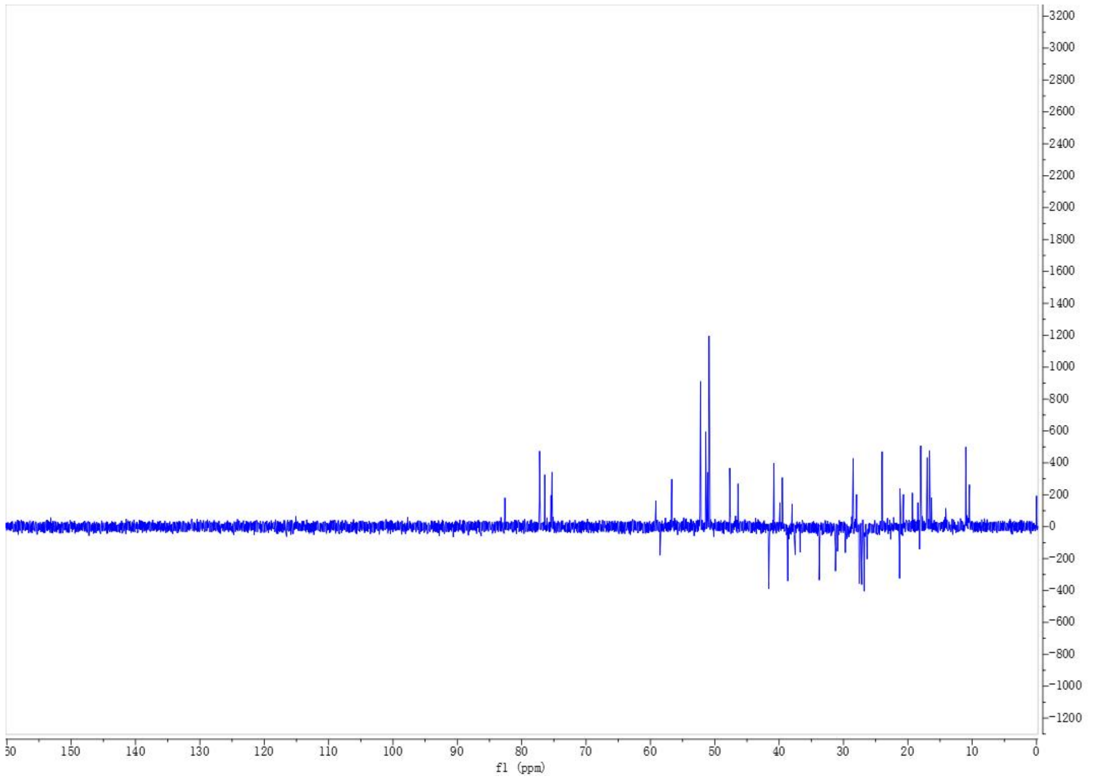

Figure S1-4. The DEPT 135 spectrum of compound **1** (151 MHz, $CDCl_3$).

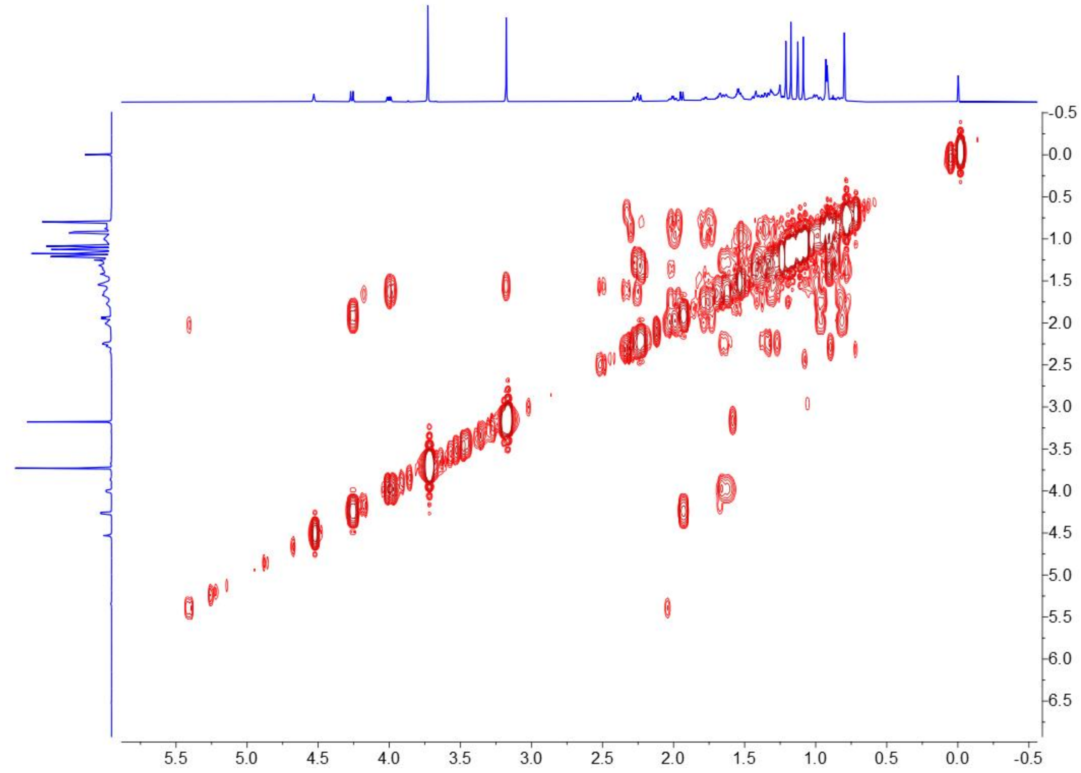

Figure S1-5. The ^{1}H-^{1}H COSY spectrum of compound **1** (600 MHz, $CDCl_3$).

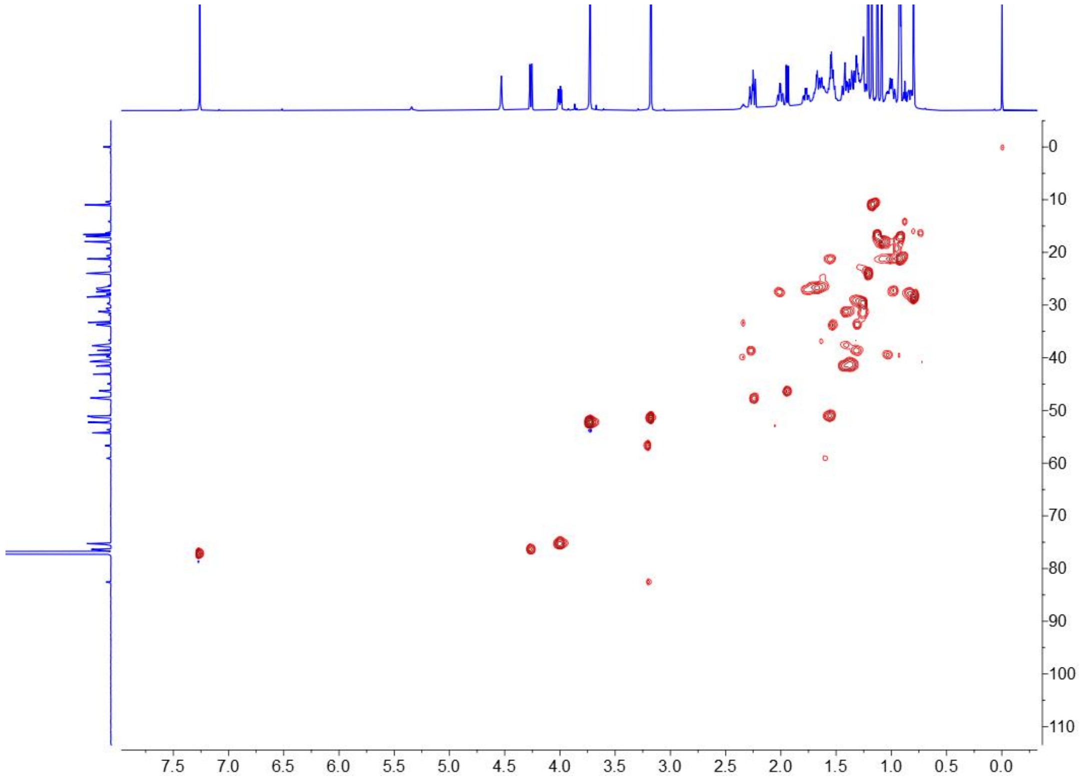

Figure S1-6. The HSQC spectrum of compound **1** (600/151 MHz, $CDCl_3$).

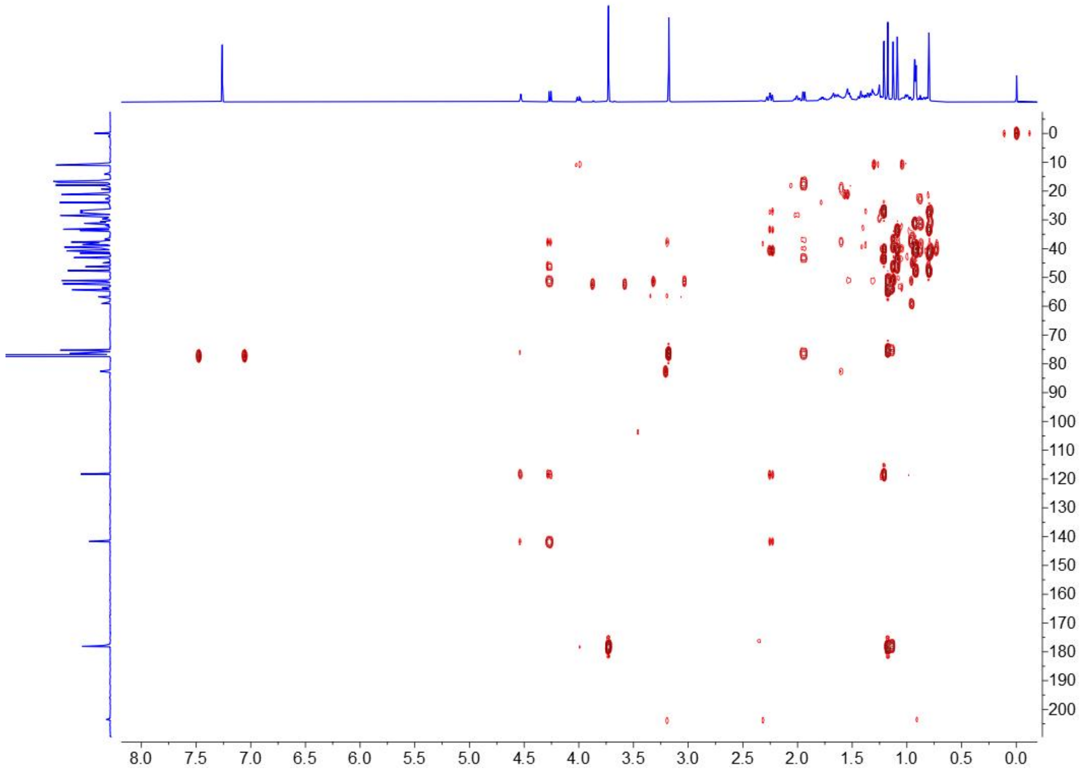

Figure S1-7. The HMBC spectrum of compound **1** (600/151 MHz, $CDCl_3$).

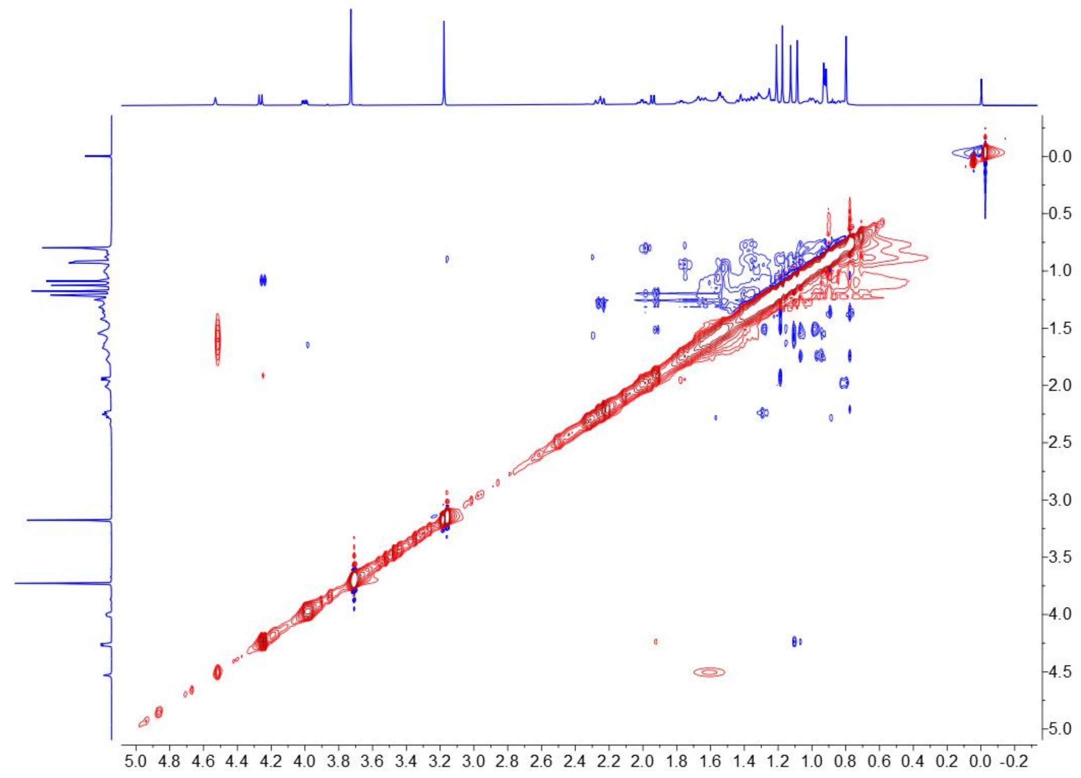

Figure S1-8. The NOESY spectrum of compound **1** (600 MHz, $CDCl_3$).

2. Spectroscopic data for compound 2

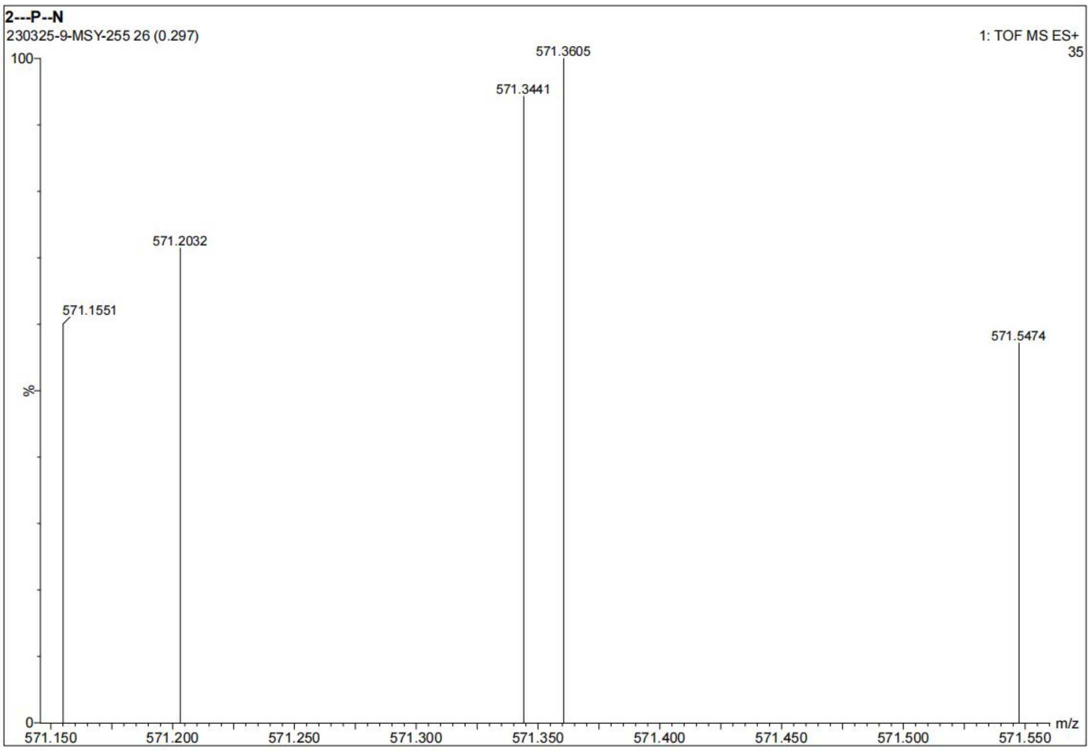

Figure S2-1. The HR-ESI-MS of compound **2**.

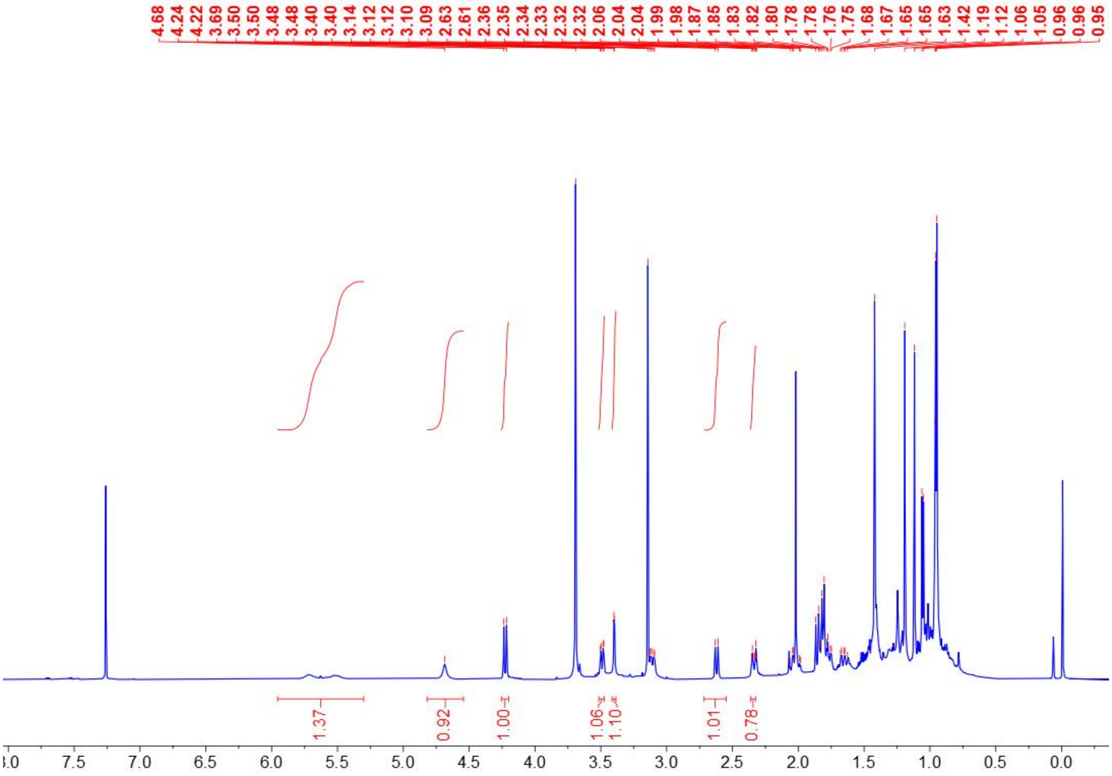

Figure S2-2. The ^{1}H NMR spectrum of compound **2** (600 MHz, $CDCl_3$).

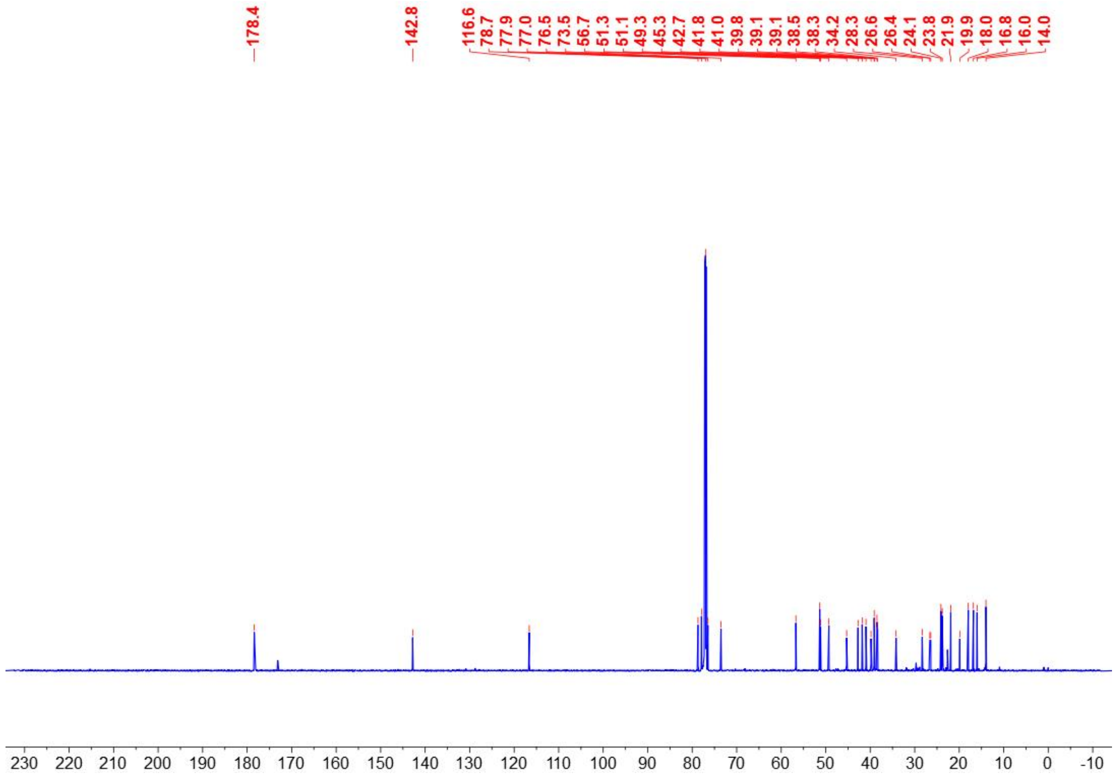

Figure S2-3. The ^{13}C NMR spectrum of compound **2** (151 MHz, $CDCl_3$).

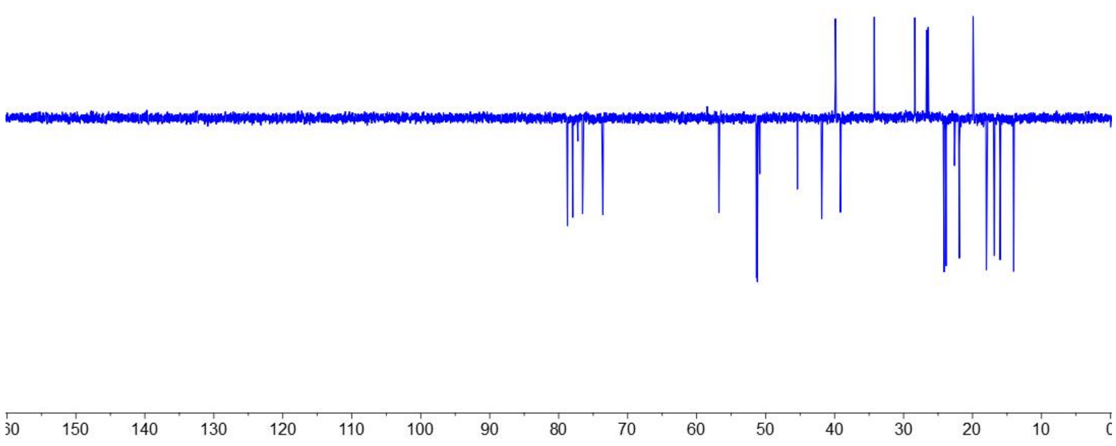

Figure S2-4. The DEPT 135 spectrum of compound **2** (151 MHz, $CDCl_3$).

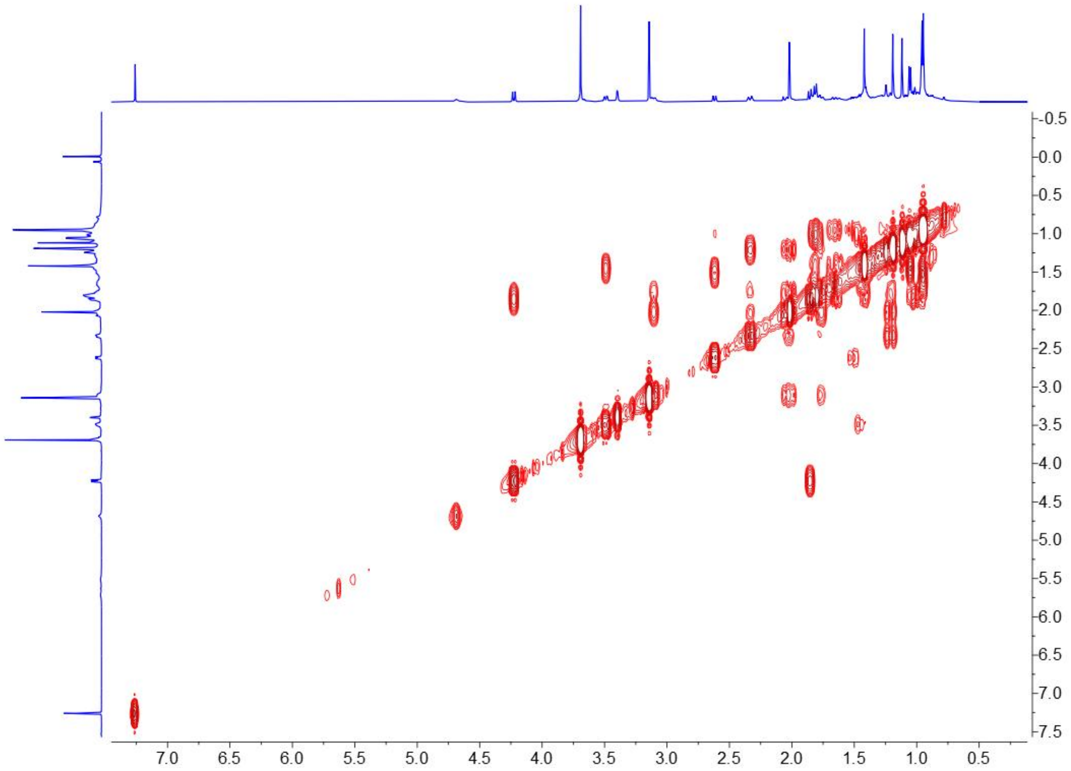

Figure S2-5. The ^{1}H-^{1}H COSY spectrum of compound **2** (600 MHz, $CDCl_3$).

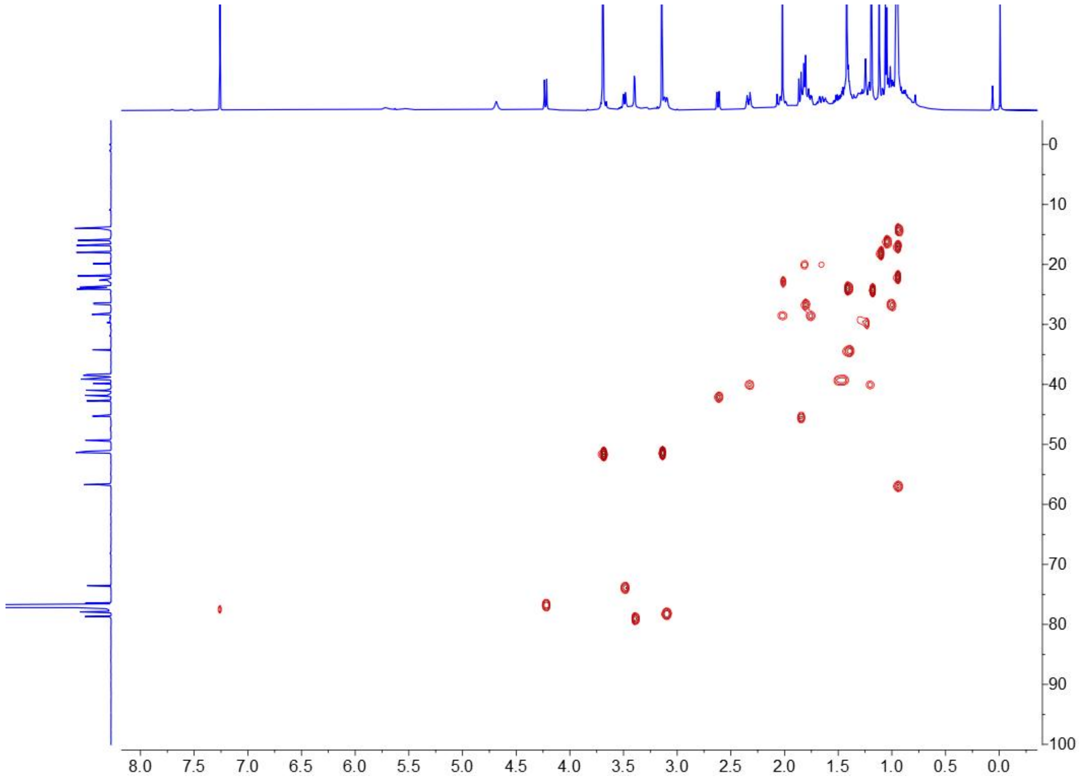

Figure S2-6. The HSQC spectrum of compound **2** (600/151 MHz, $CDCl_3$).

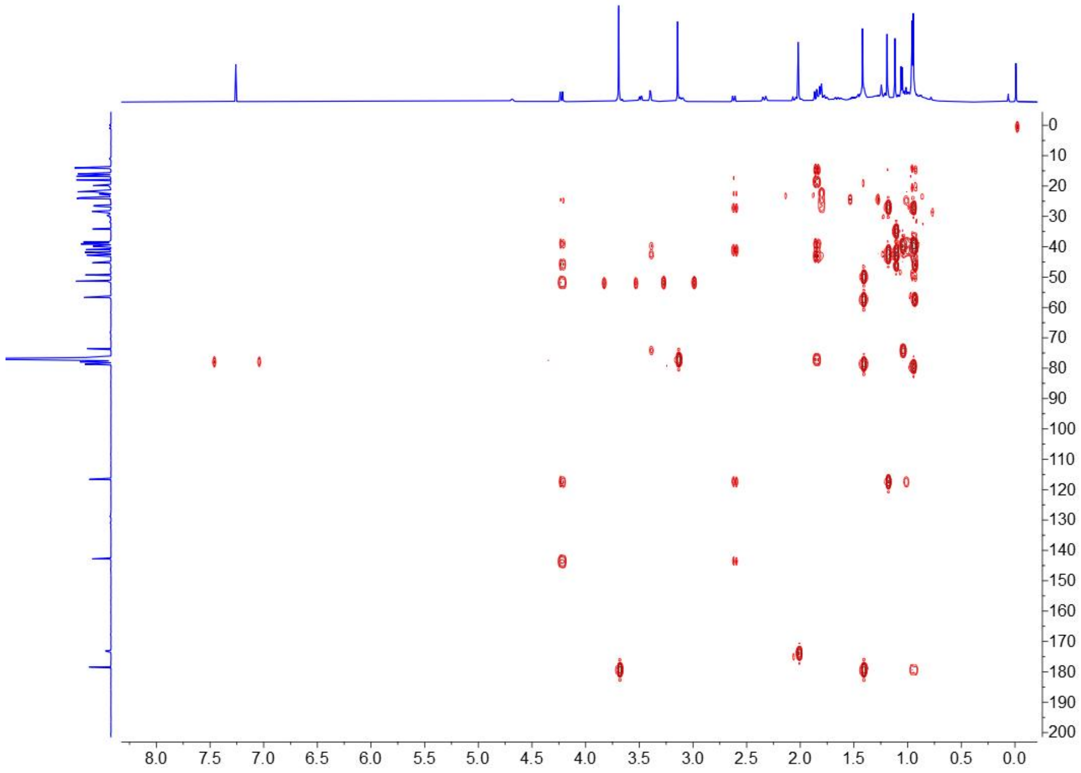

Figure S2-7. The HMBC spectrum of compound **2** (600/151 MHz, $CDCl_3$).

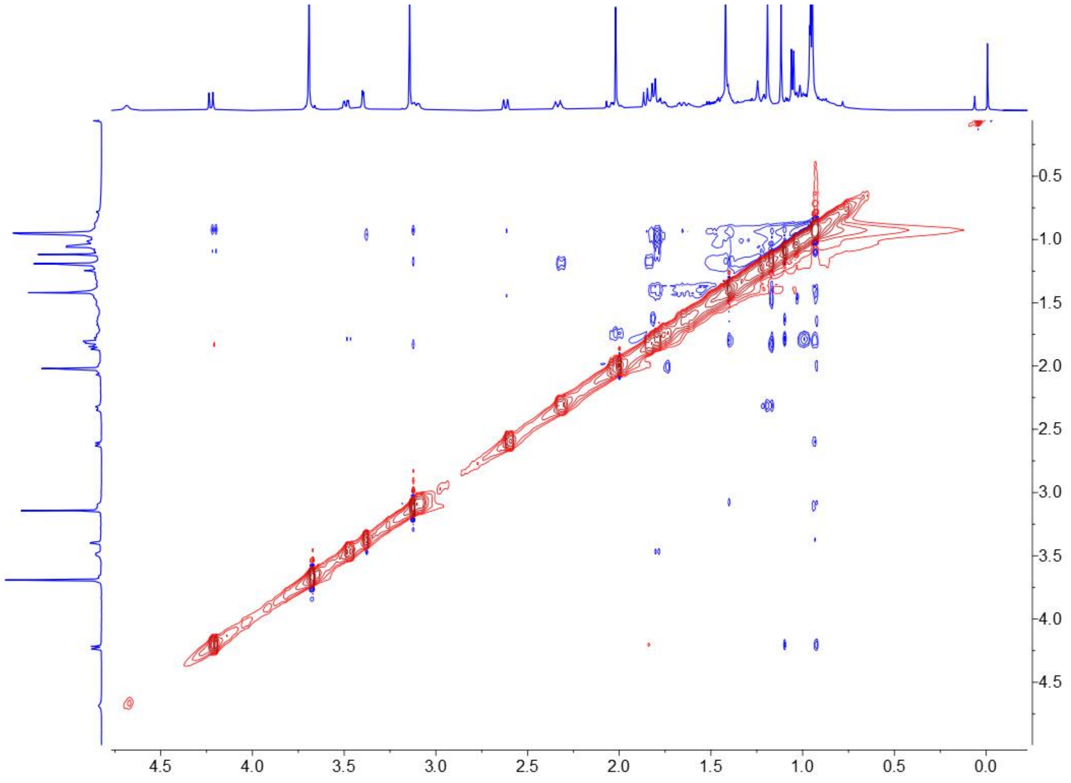

Figure S2-8. The NOESY spectrum of compound **2** (600 MHz, $CDCl_3$).

3. Spectroscopic data for compound 3

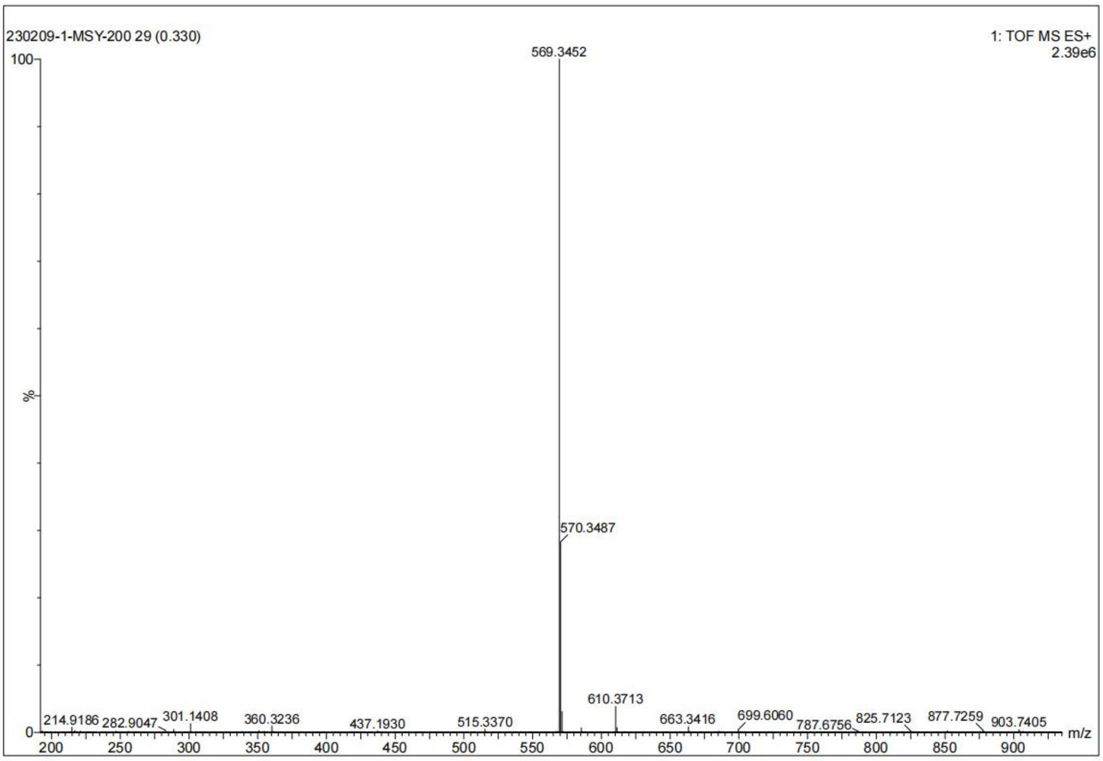

Figure S3-1. The HR-ESI-MS of compound **3**.

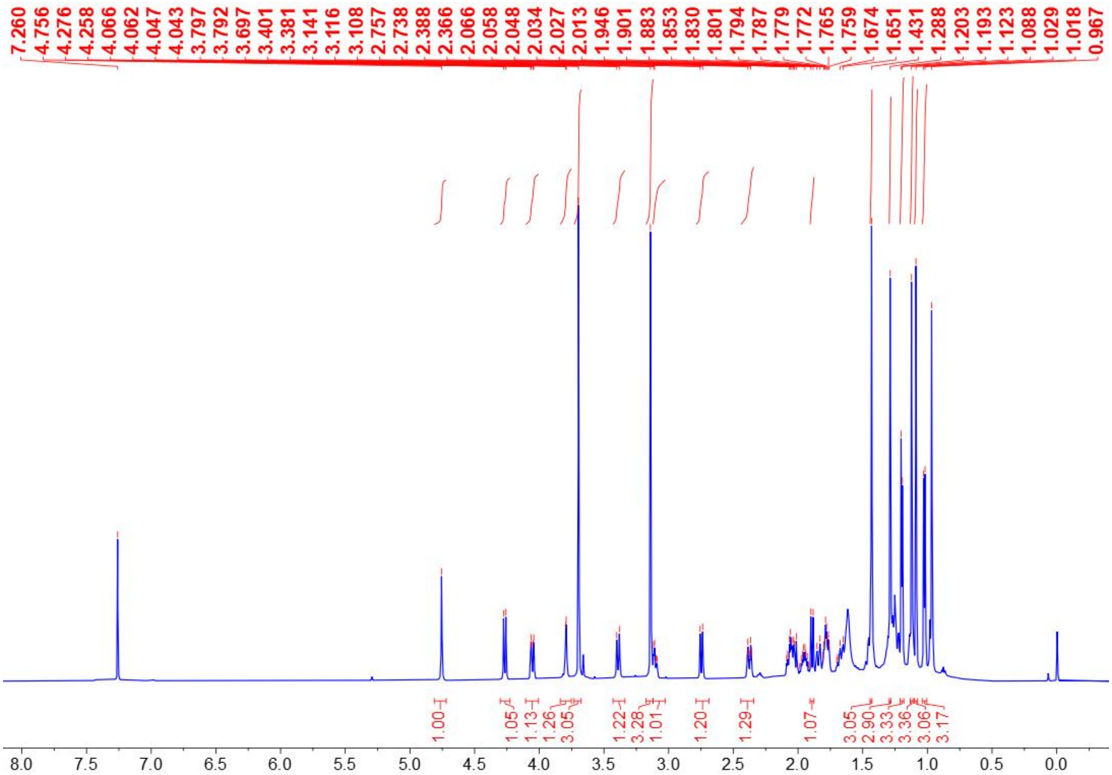

Figure S3-2. The ^{1}H NMR spectrum of compound **3** (600 MHz, $CDCl_3$).

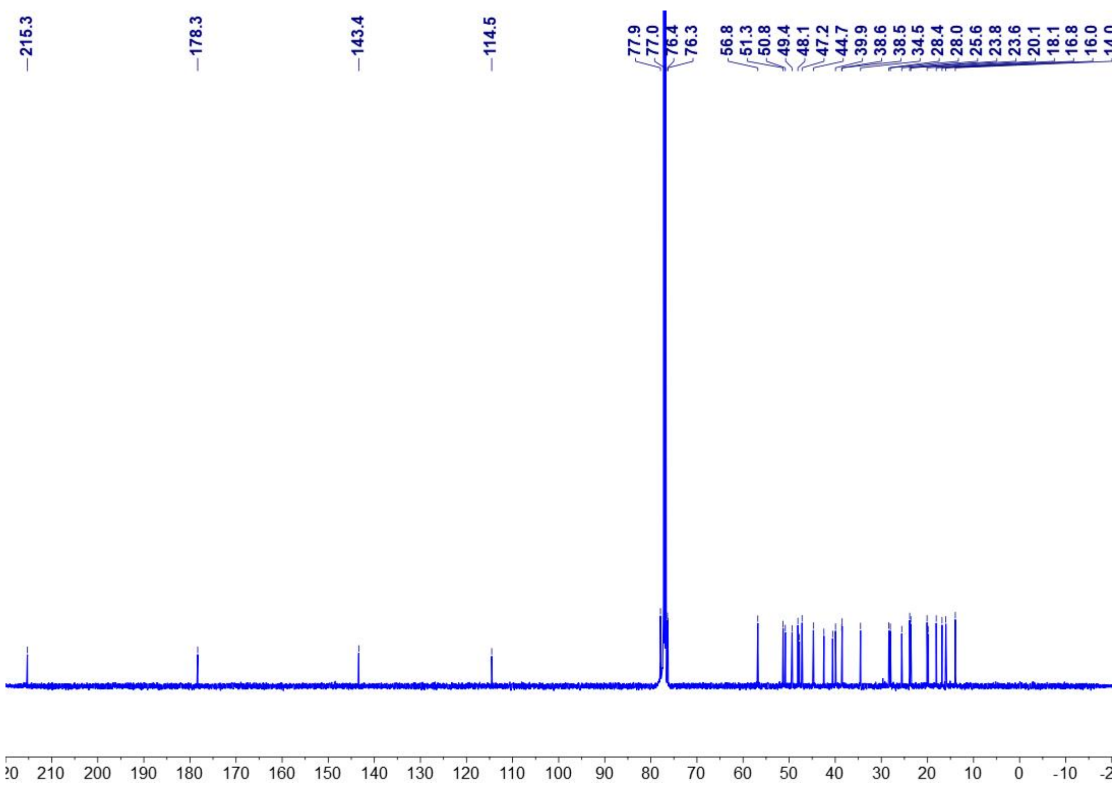

Figure S3-3. The ^{13}C NMR spectrum of compound **3** (151 MHz, $CDCl_3$).

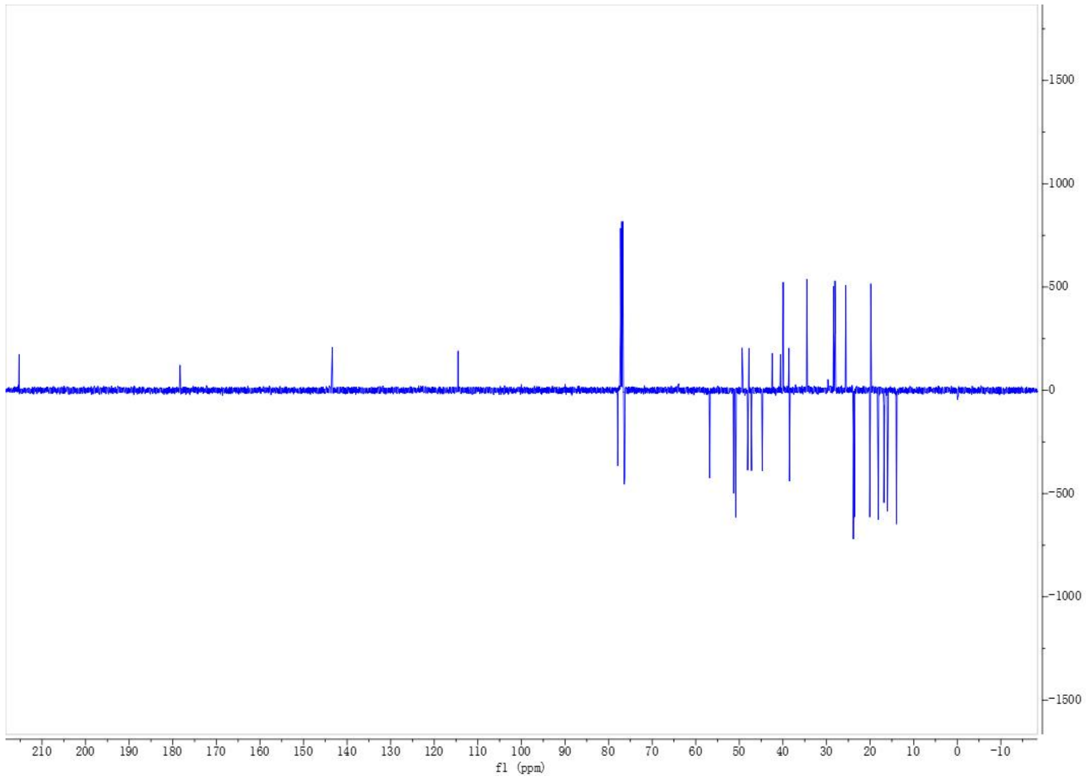

Figure S3-4. The DEPT 135 spectrum of compound **3** (151 MHz, $CDCl_3$).

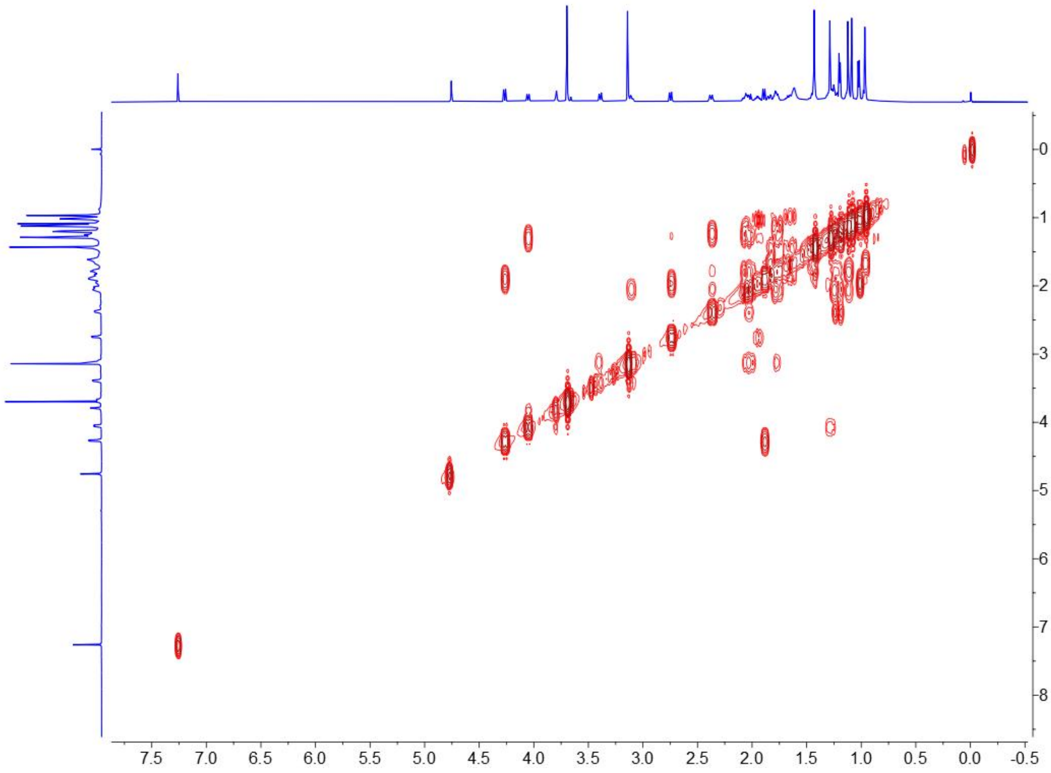

Figure S3-5. The ^{1}H-^{1}H COSY spectrum of compound **3** (600 MHz, $CDCl_3$).

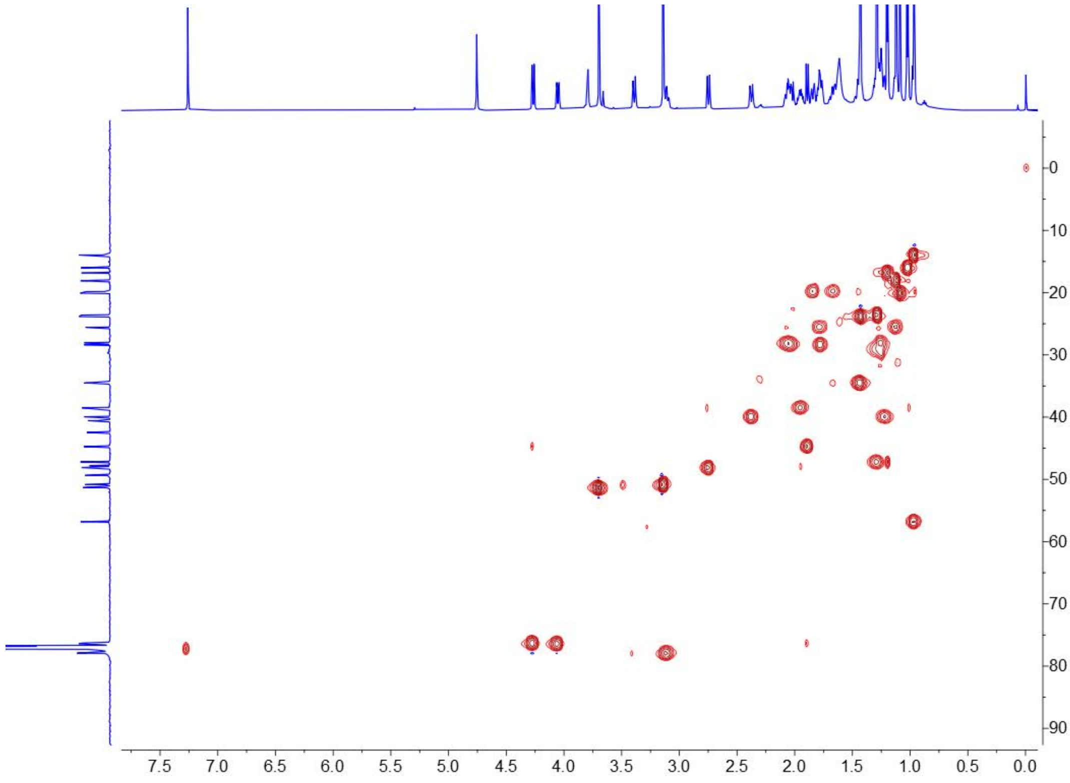

Figure S3-6. The HSQC spectrum of compound **3** (600/151 MHz, $CDCl_3$).

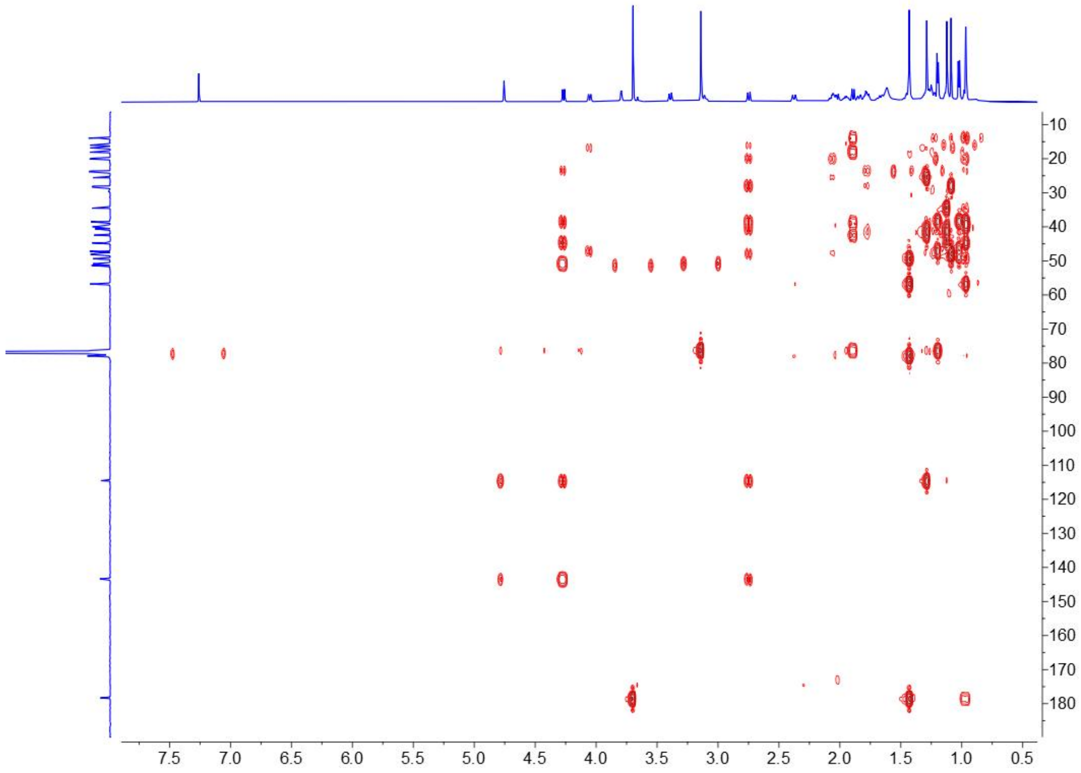

Figure S3-7. The HMBC spectrum of compound **3** (600/151 MHz, $CDCl_3$).

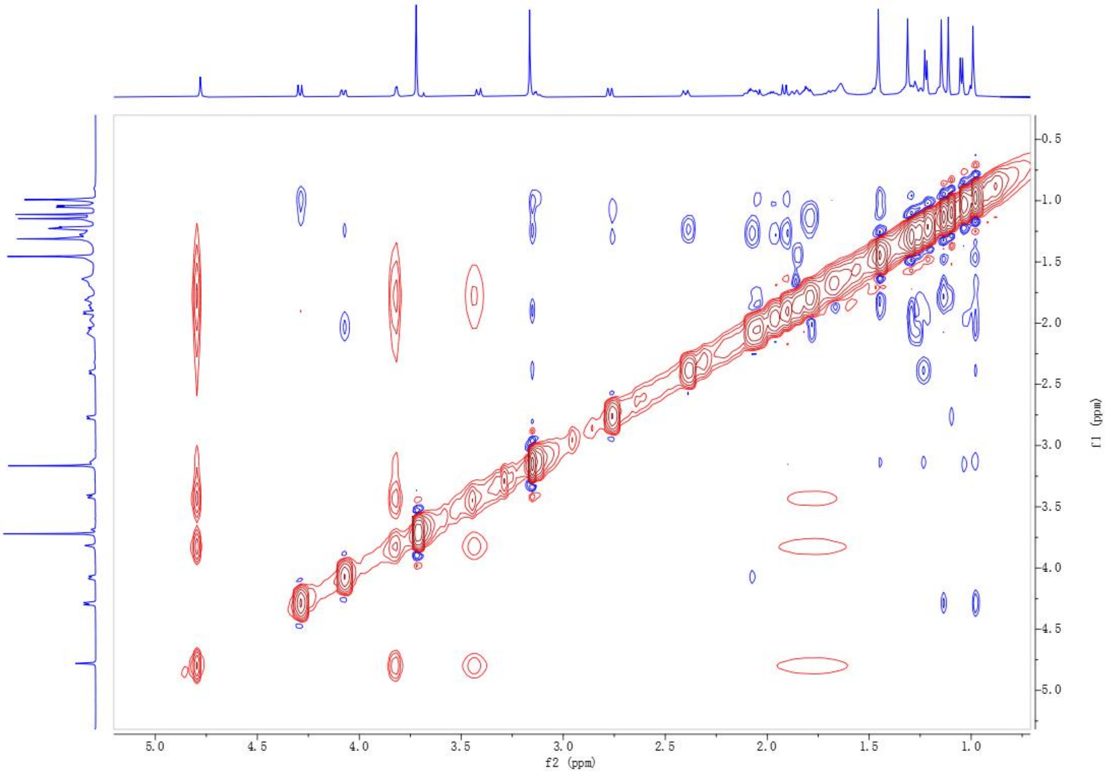

Figure S3-8. The NOESY spectrum of compound **3** (600 MHz, $CDCl_3$).

4. Spectroscopic data for compound 4

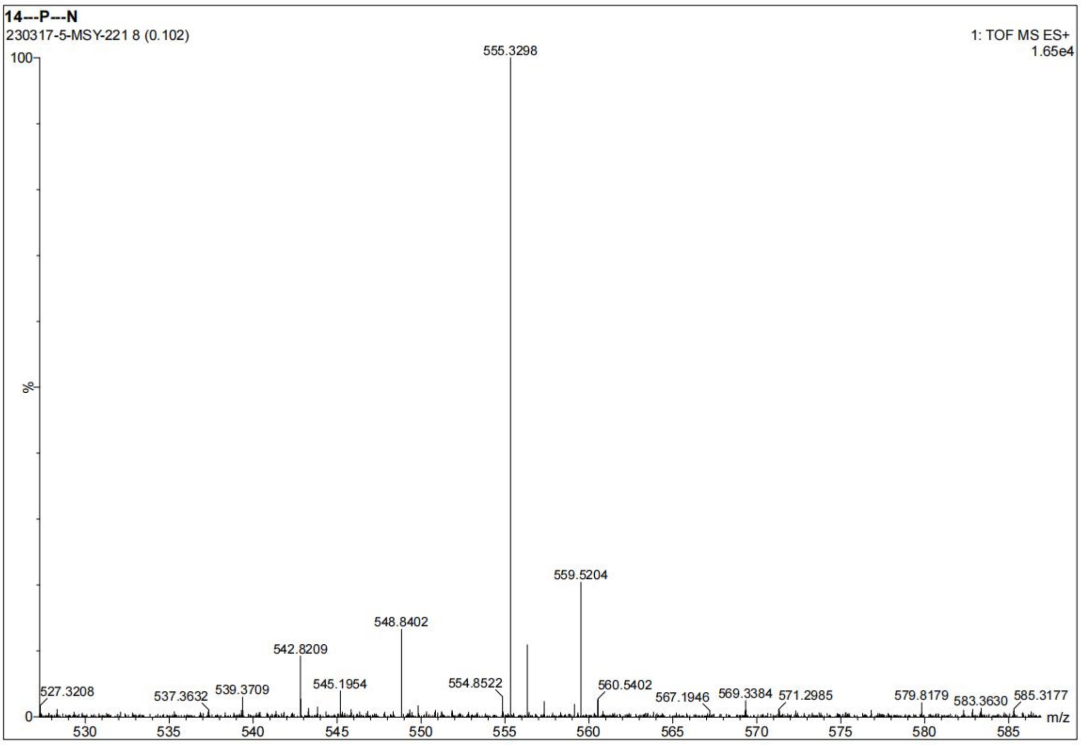

Figure S4-1. The HR-ESI-MS of compound **4**.

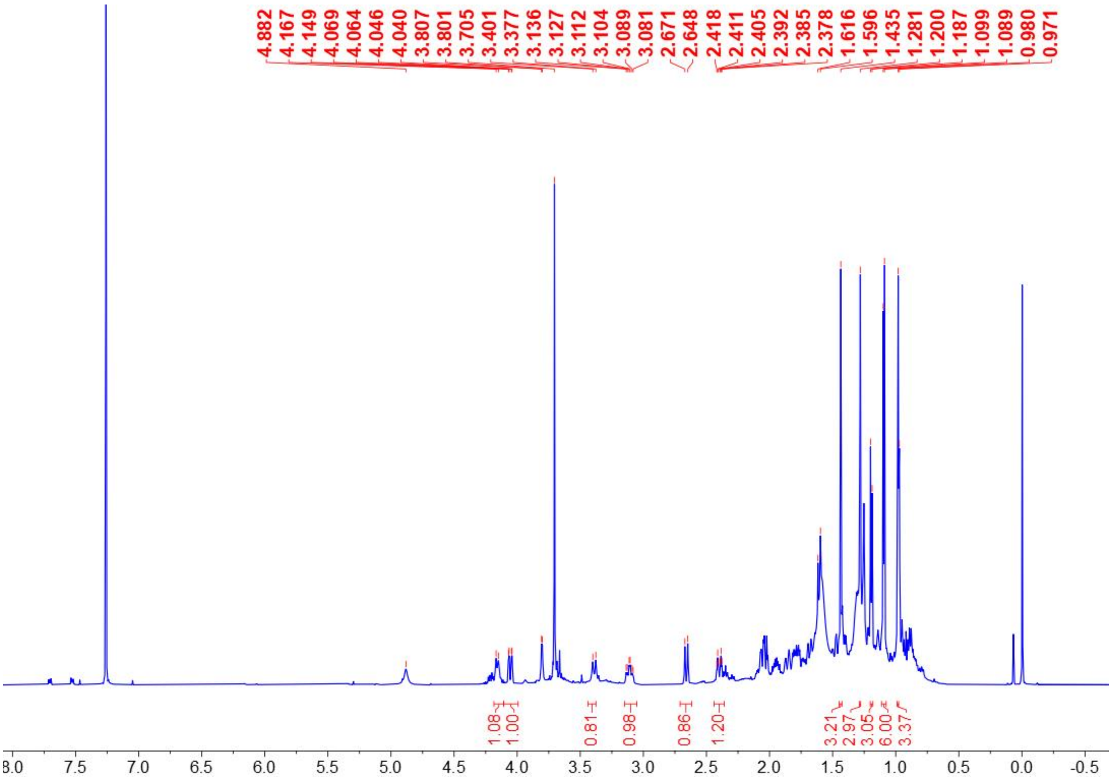

Figure S4-2. The ^{1}H NMR spectrum of compound **4** (600 MHz, $CDCl_3$).

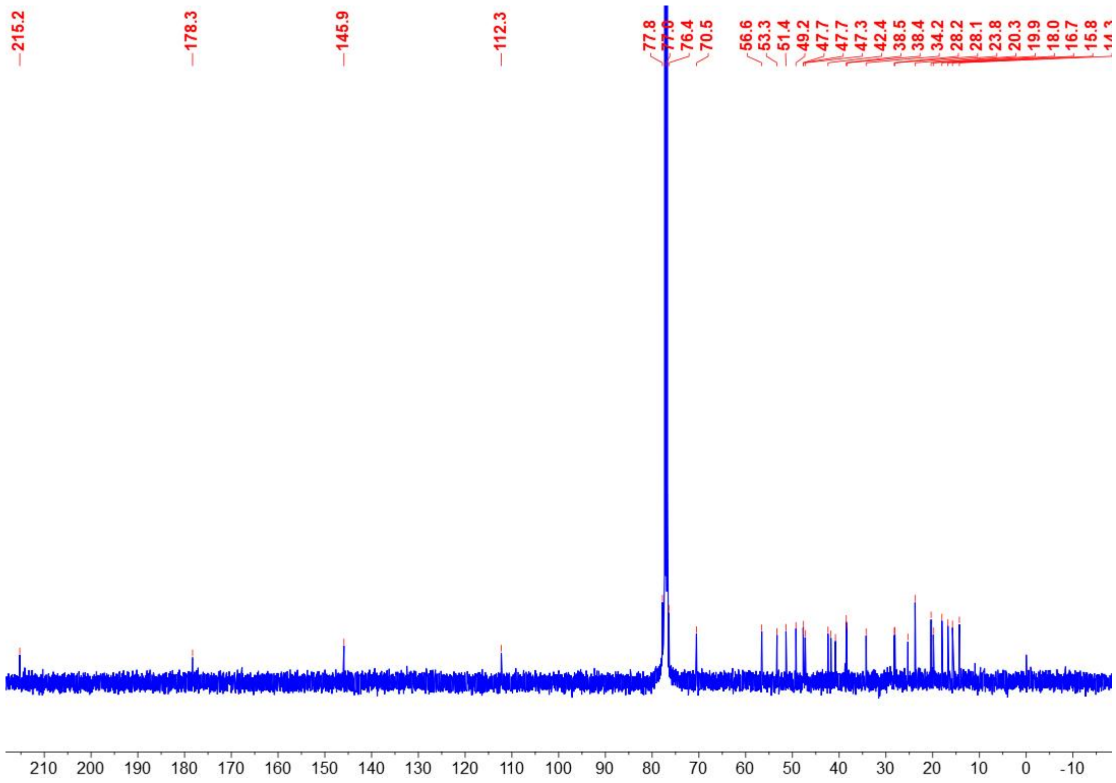

Figure S4-3. The ^{13}C NMR spectrum of compound **4** (151 MHz, $CDCl_3$).

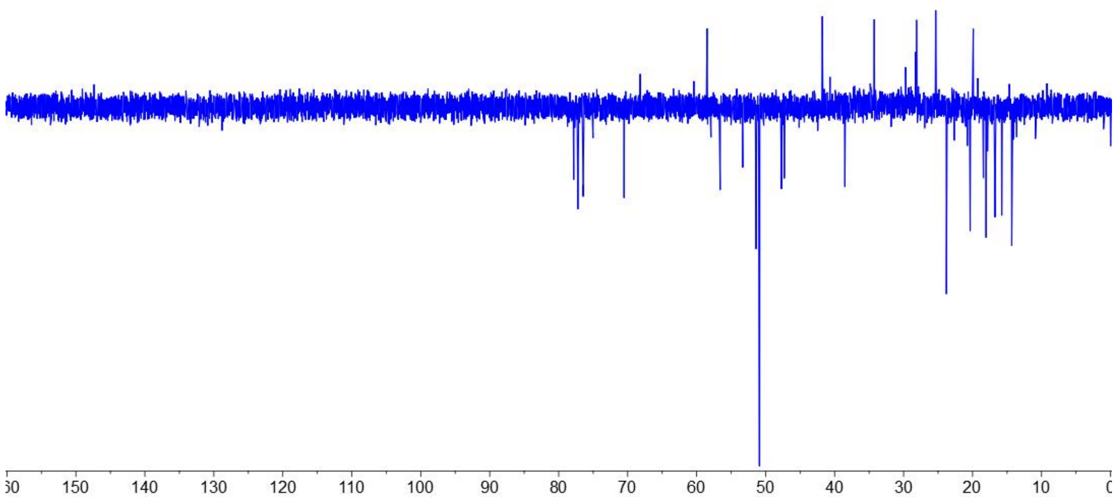

Figure S4-4. The DEPT 135 spectrum of compound **4** (151 MHz, $CDCl_3$).

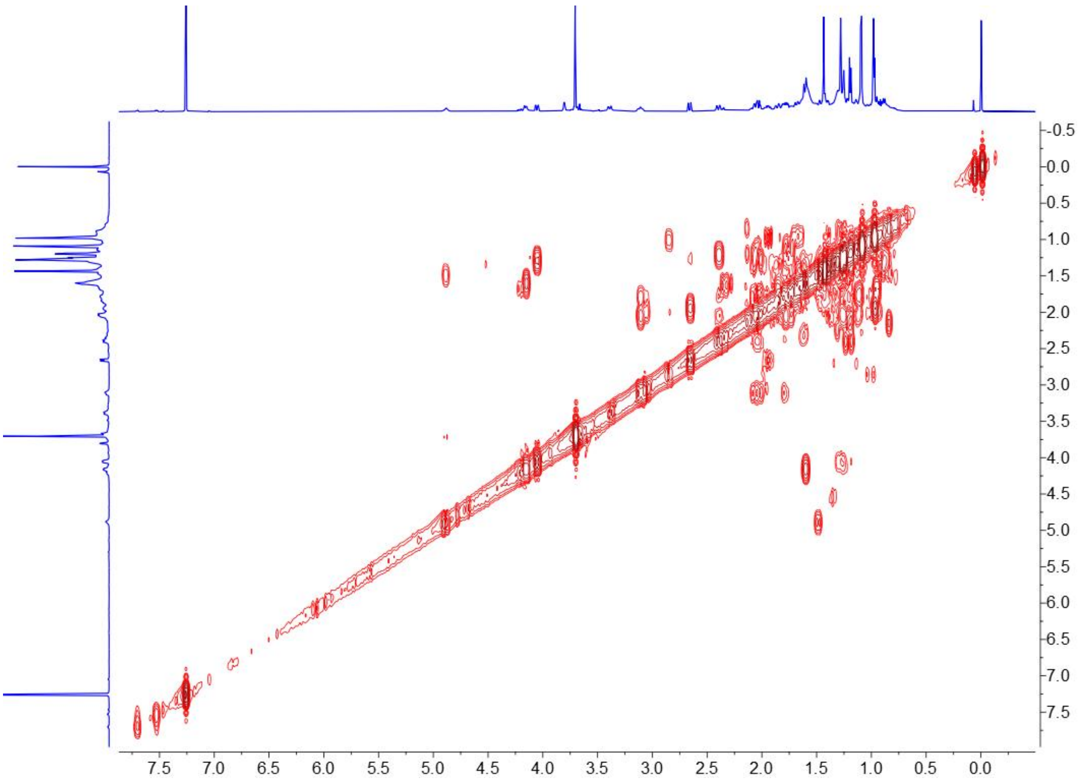

Figure S4-5. The ^{1}H-^{1}H COSY spectrum of compound **4** (600 MHz, $CDCl_3$).

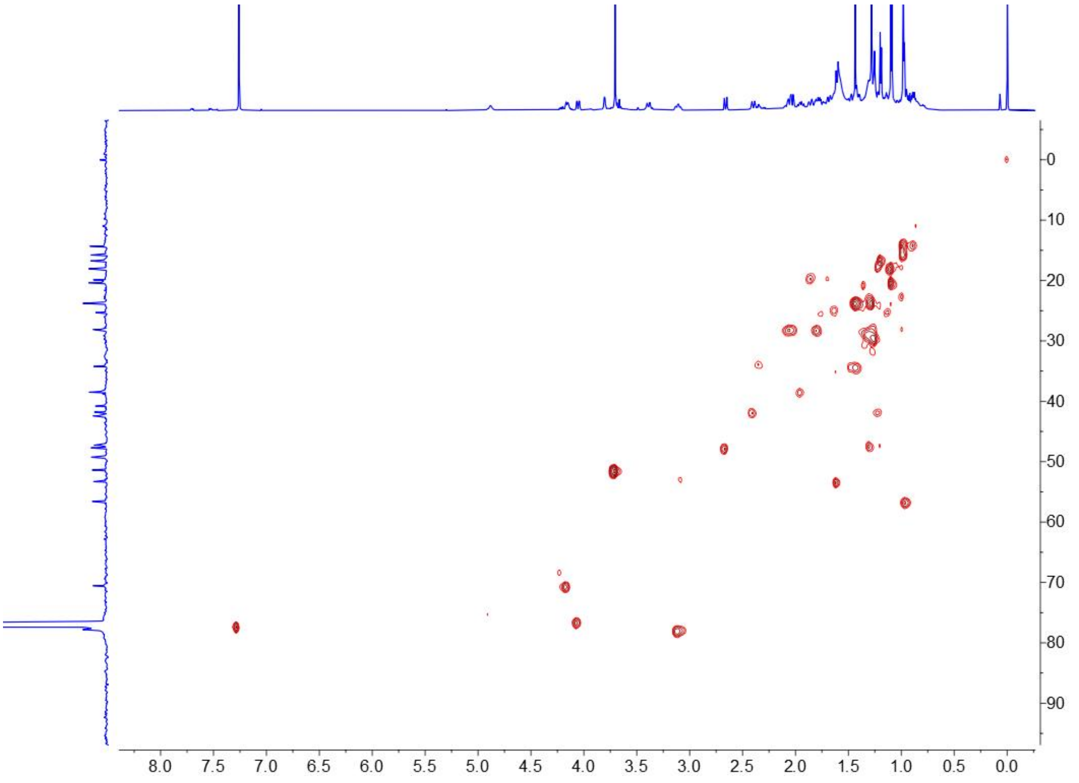

Figure S4-6. The HSQC spectrum of compound **4** (600/151 MHz, $CDCl_3$).

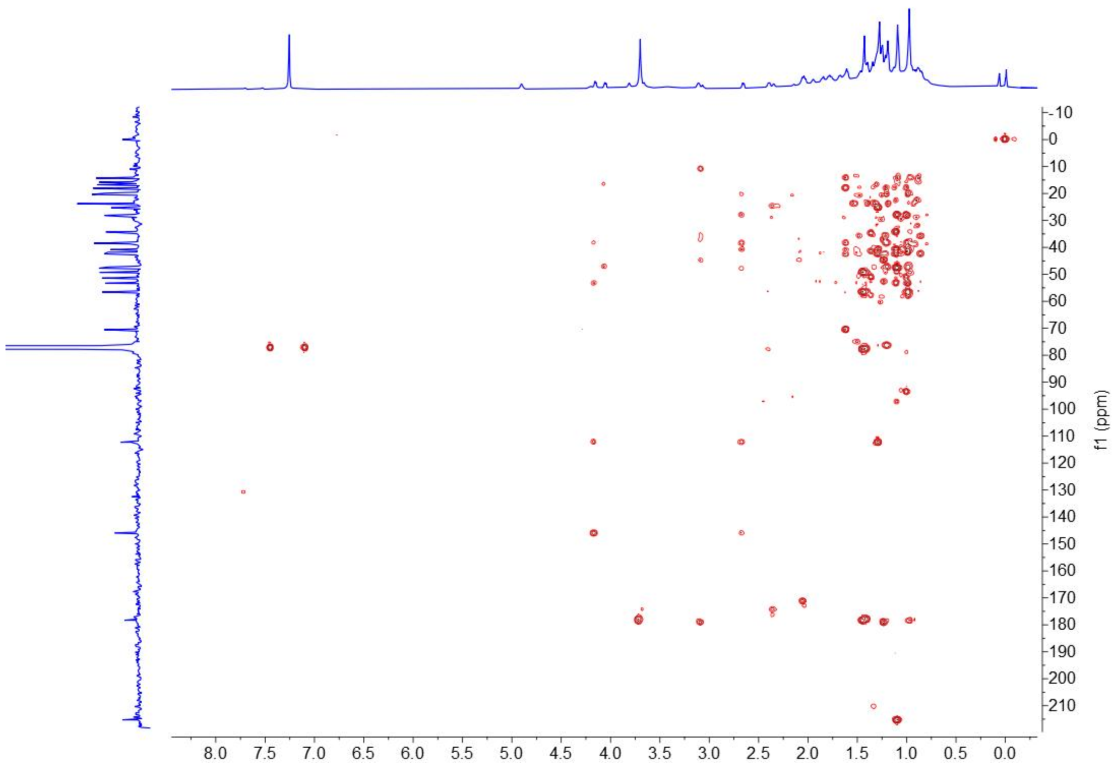

Figure S4-7. The HMBC spectrum of compound **4** (600/151 MHz, $CDCl_3$).

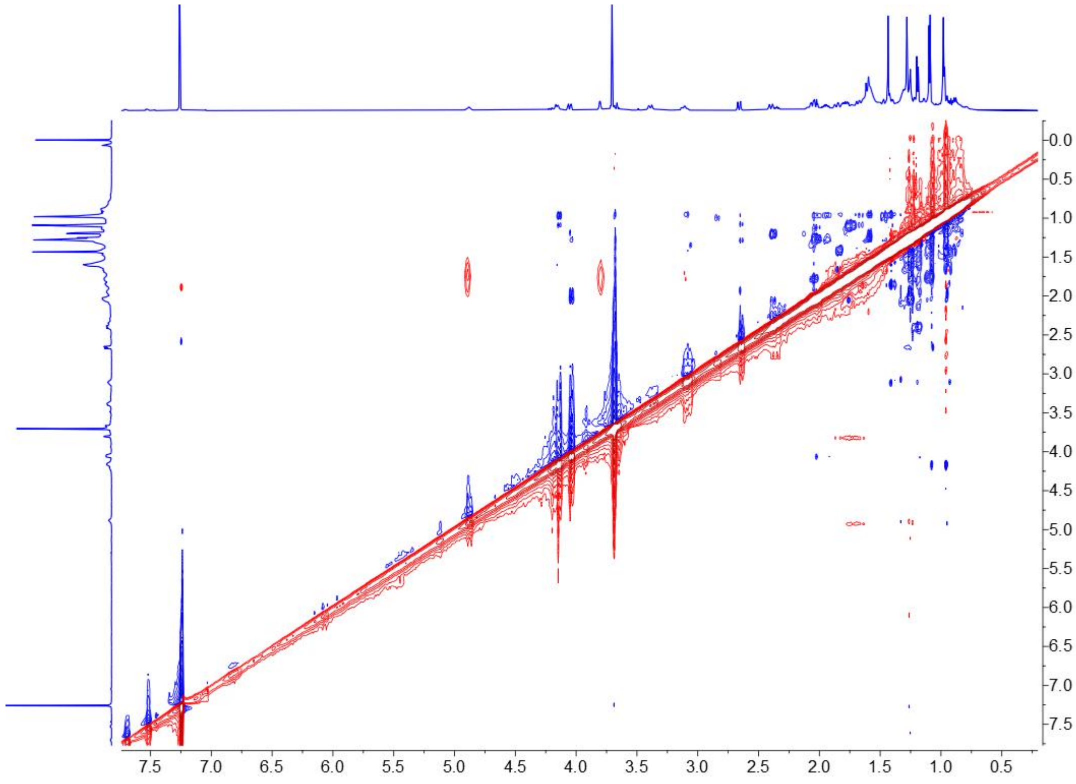

Figure S4-8. The NOESY spectrum of compound **4** (600 MHz, $CDCl_3$).

5. Spectroscopic data for compound 5

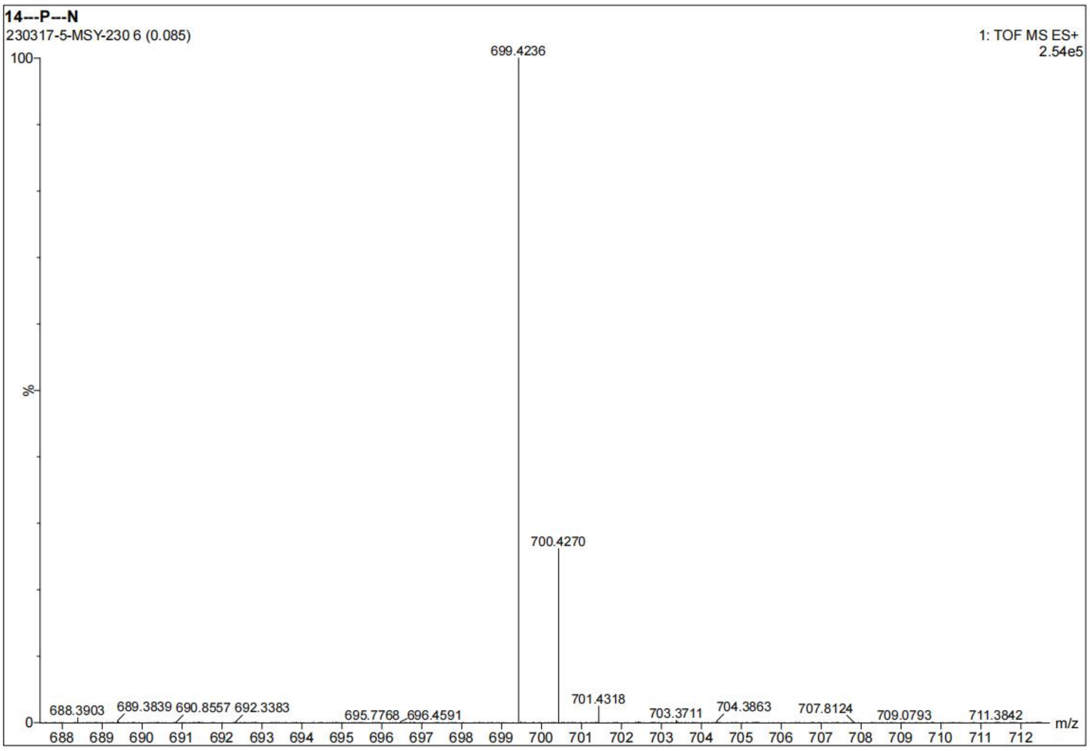

Figure S5-1. The HR-ESI-MS of compound **5**.

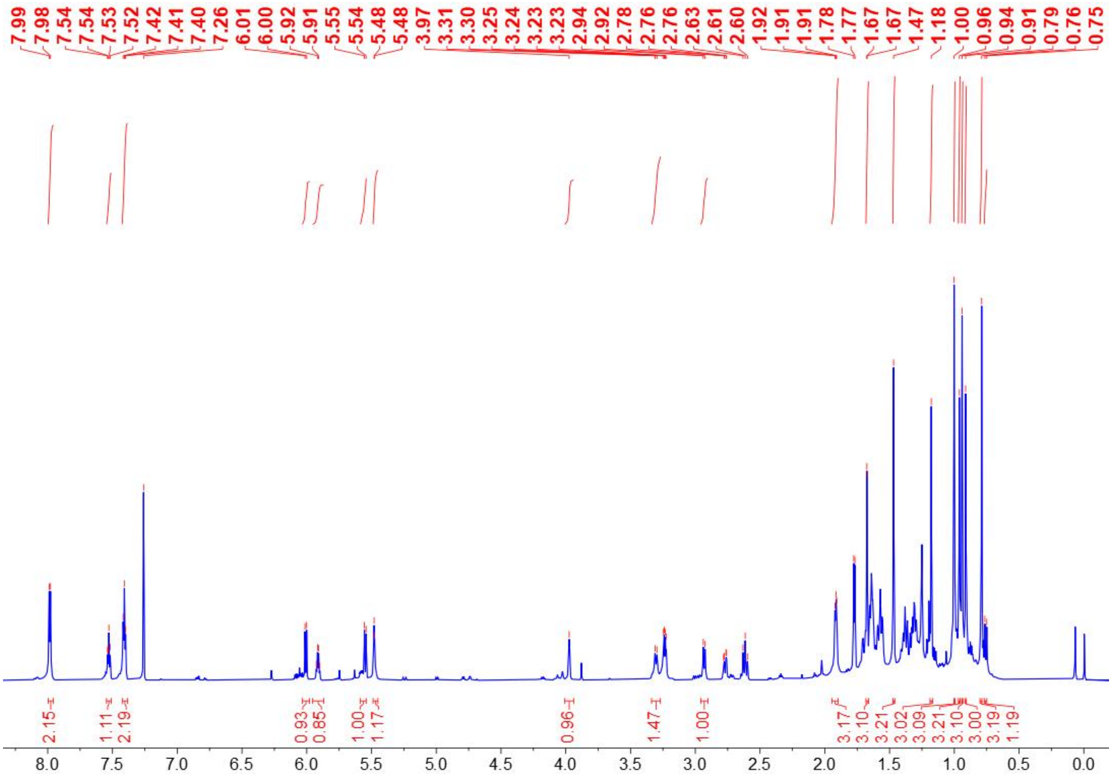

Figure S5-2. The ^{1}H NMR spectrum of compound **5** (600 MHz, $CDCl_3$).

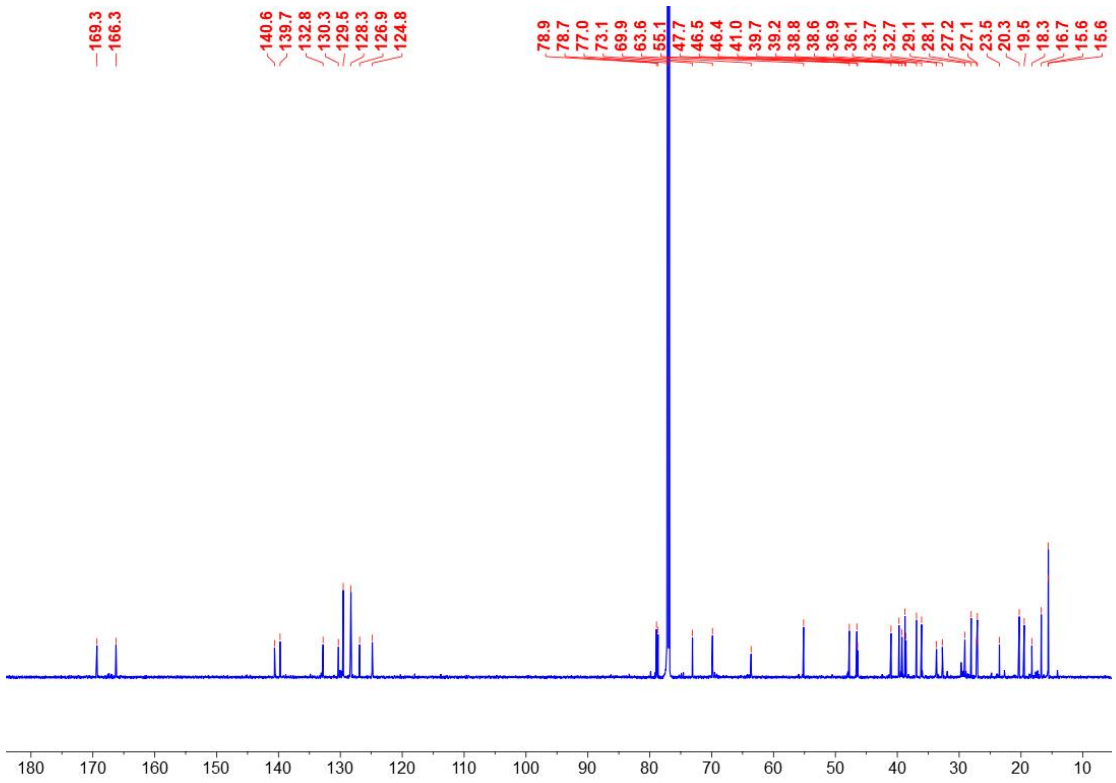

Figure S5-3. The ^{13}C NMR spectrum of compound **5** (151 MHz, $CDCl_3$).

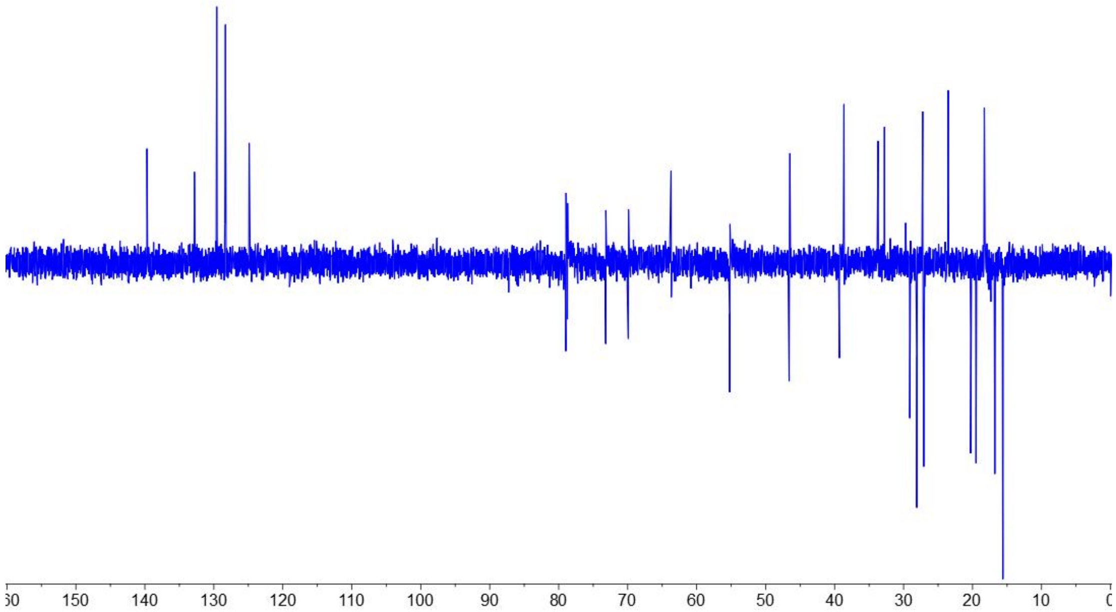

Figure S5-4. The DEPT 135 spectrum of compound **5** (151 MHz, $CDCl_3$).

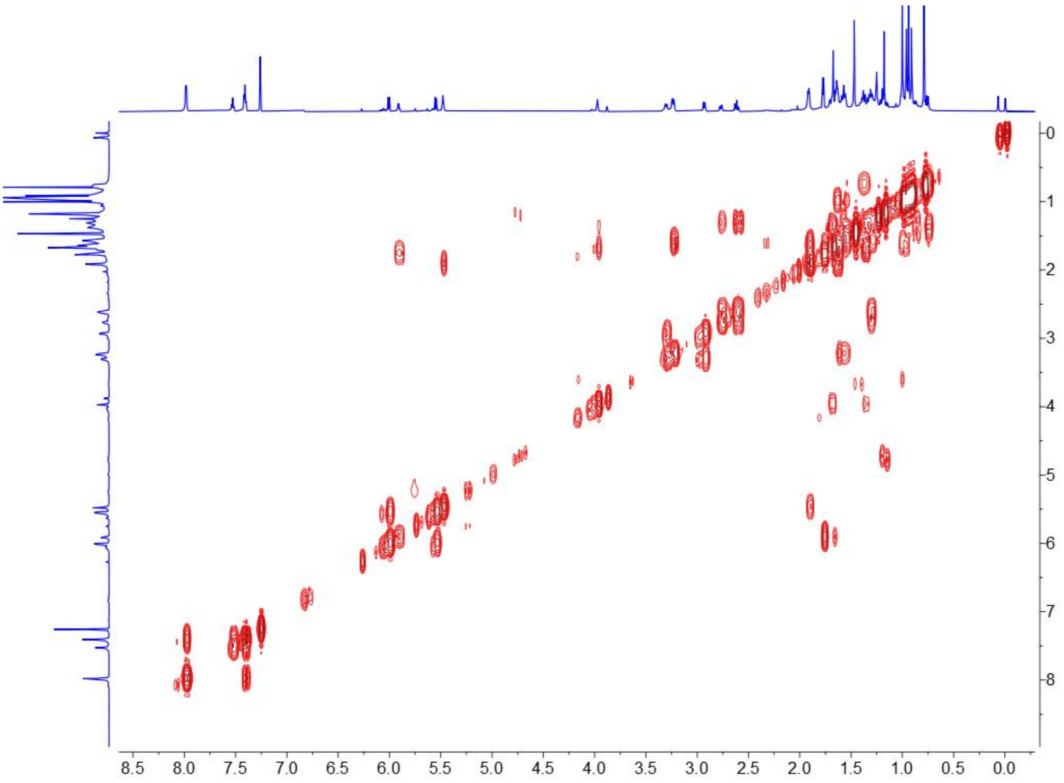

Figure S5-5. The ^{1}H-^{1}H COSY spectrum of compound **5** (600 MHz, $CDCl_3$).

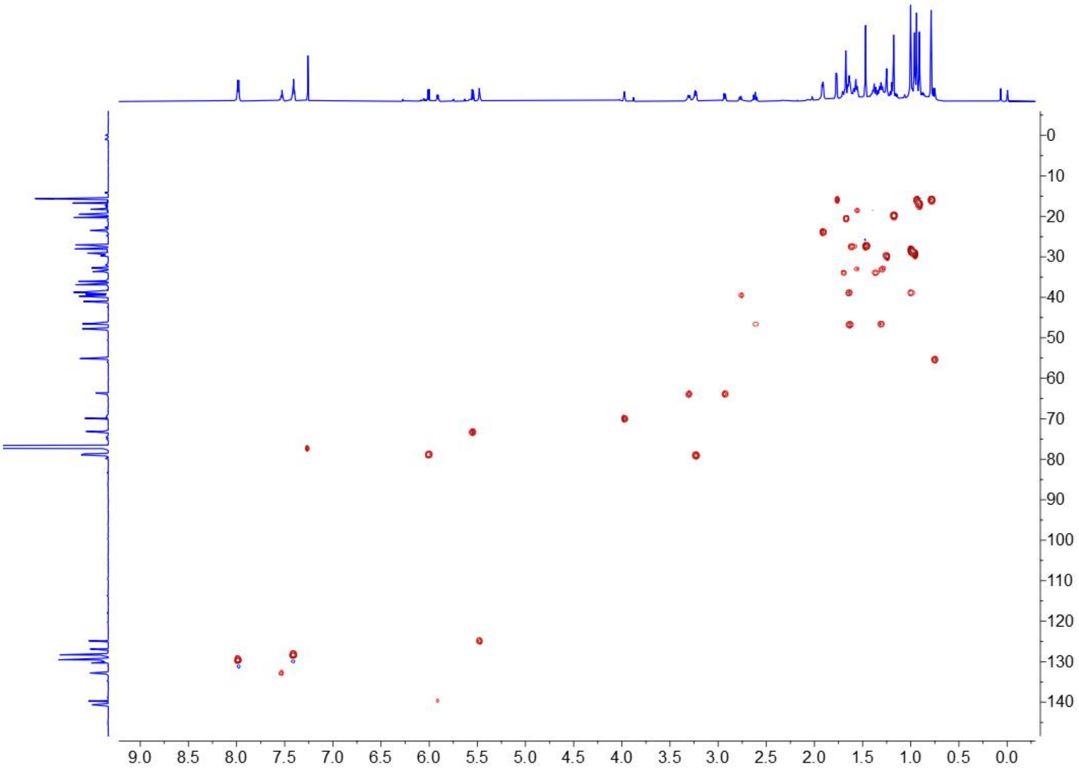

Figure S5-6. The HSQC spectrum of compound **5** (600/151 MHz, $CDCl_3$).

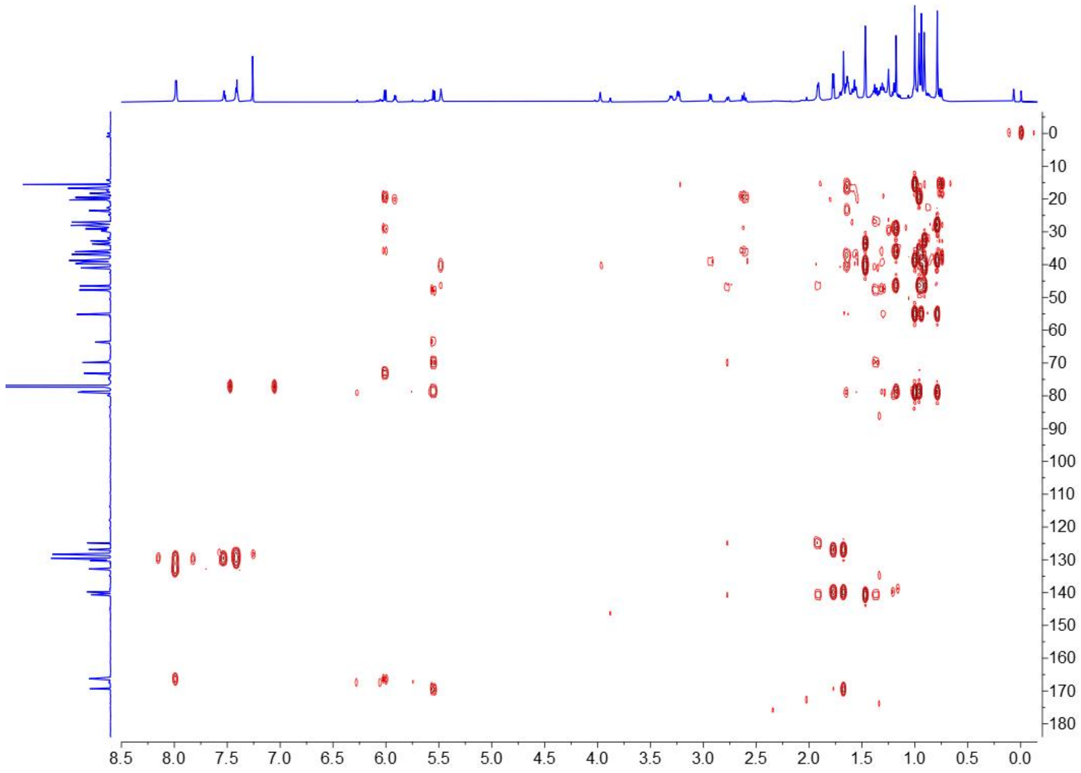

Figure S5-7. The HMBC spectrum of compound **5** (600/151 MHz, $CDCl_3$).

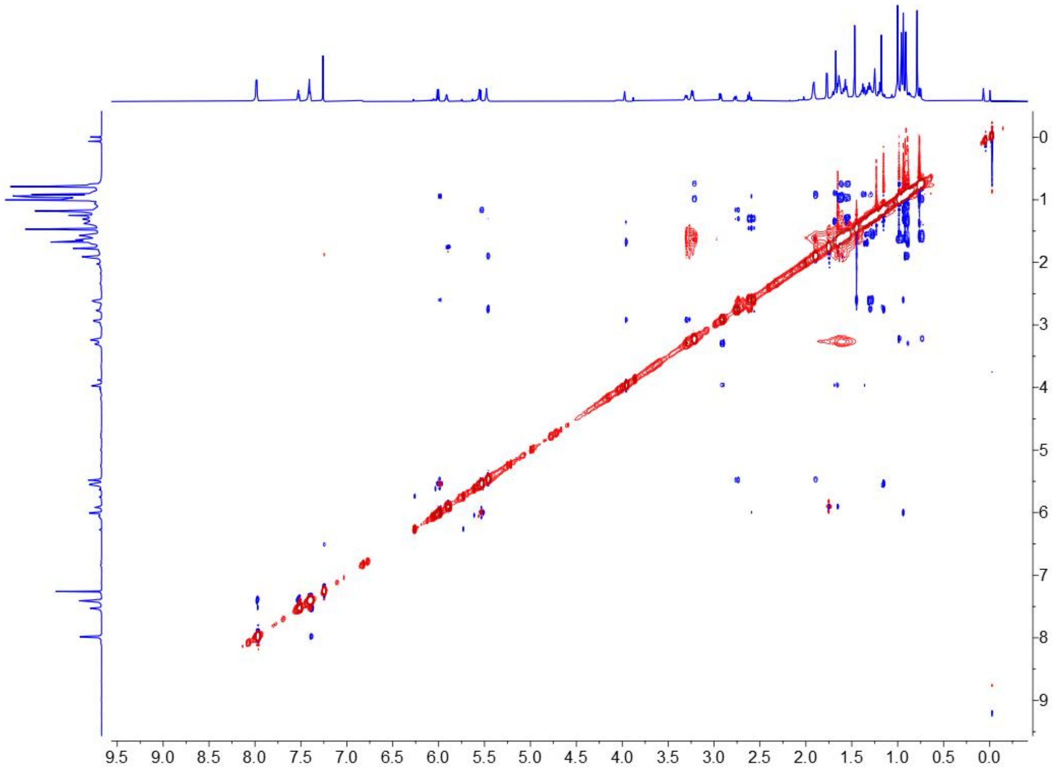

Figure S5-8. The NOESY spectrum of compound **5** (600 MHz, $CDCl_3$).

6. Spectroscopic data for compound 6

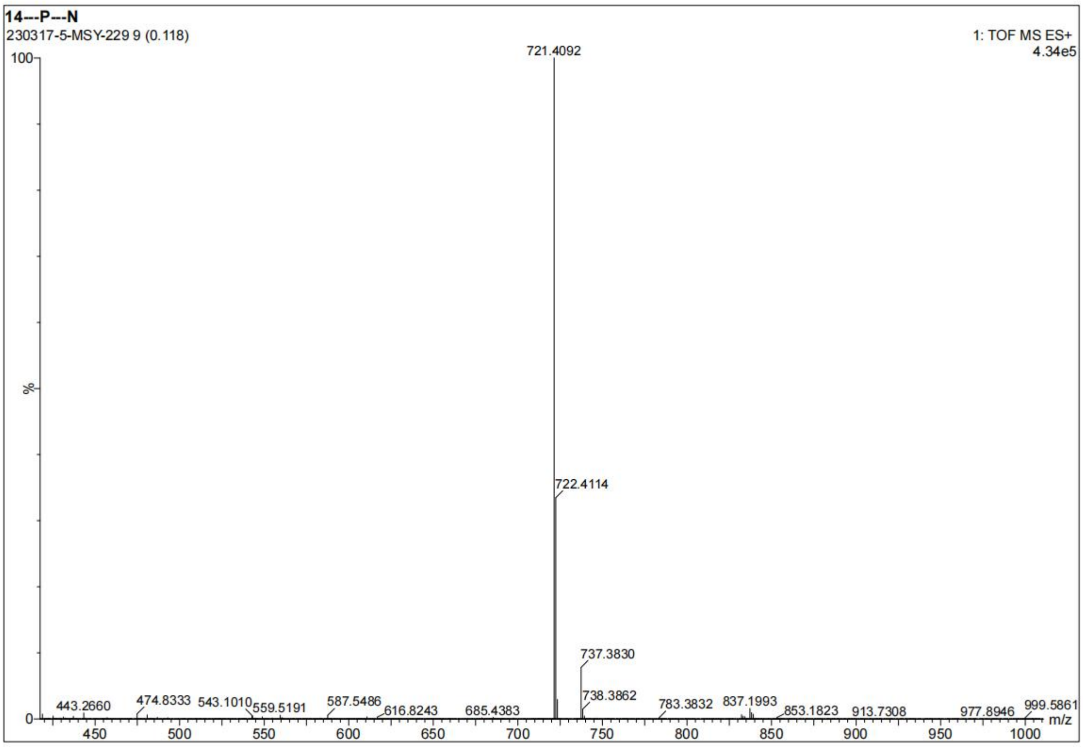

Figure S6-1. The HR-ESI-MS of compound **6**.

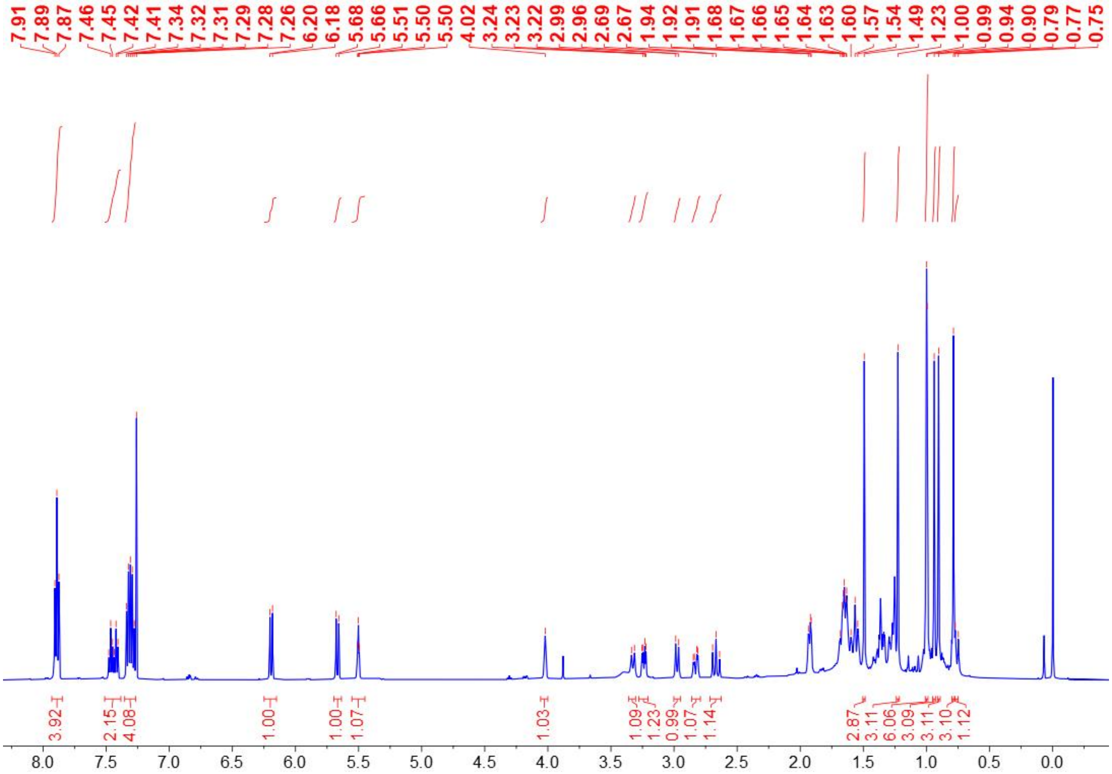

Figure S6-2. The ^{1}H NMR spectrum of compound **6** (600 MHz, $CDCl_3$).

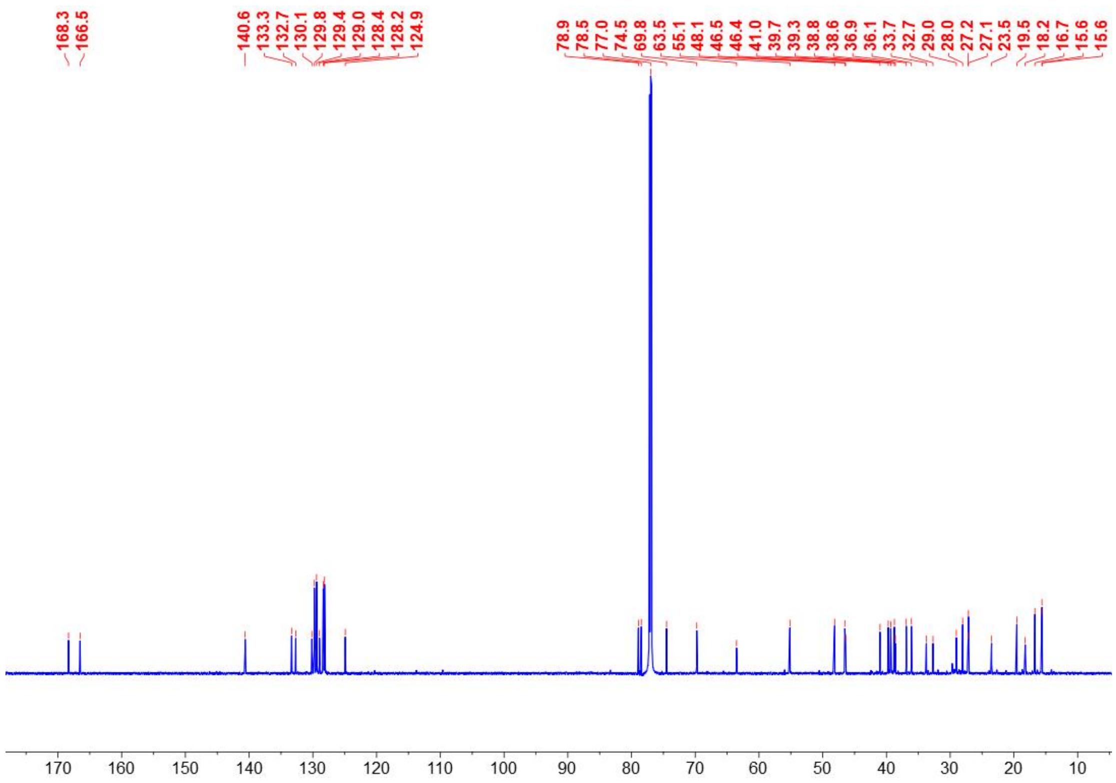

Figure S6-3. The ^{13}C NMR spectrum of compound **6** (151 MHz, $CDCl_3$).

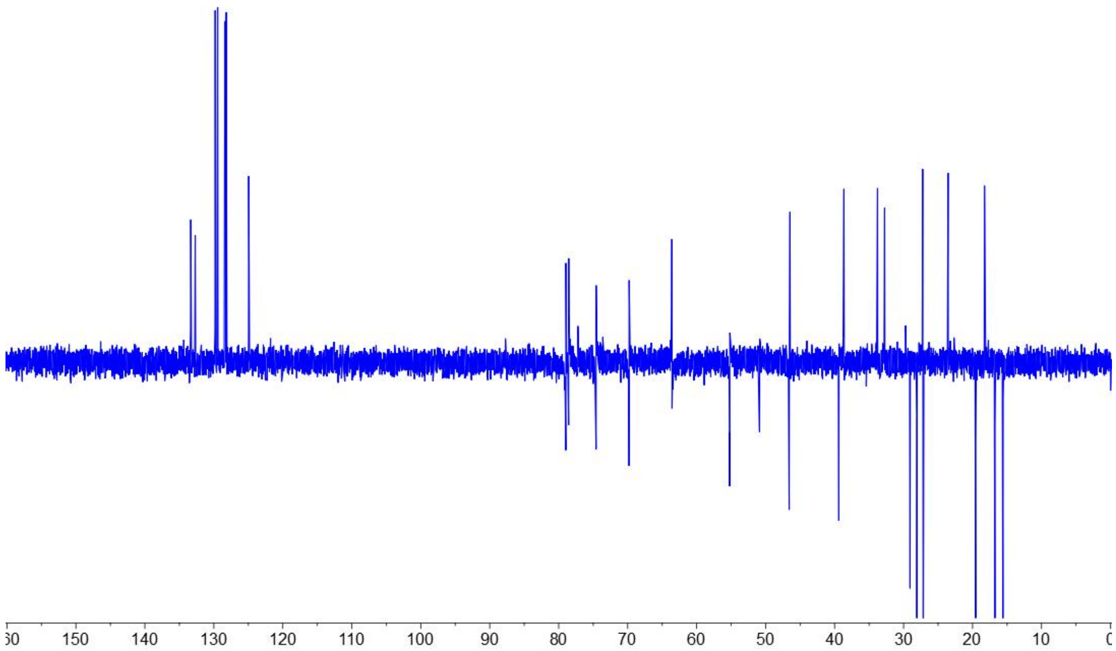

Figure S6-4. The DEPT 135 spectrum of compound **6** (151 MHz, $CDCl_3$).

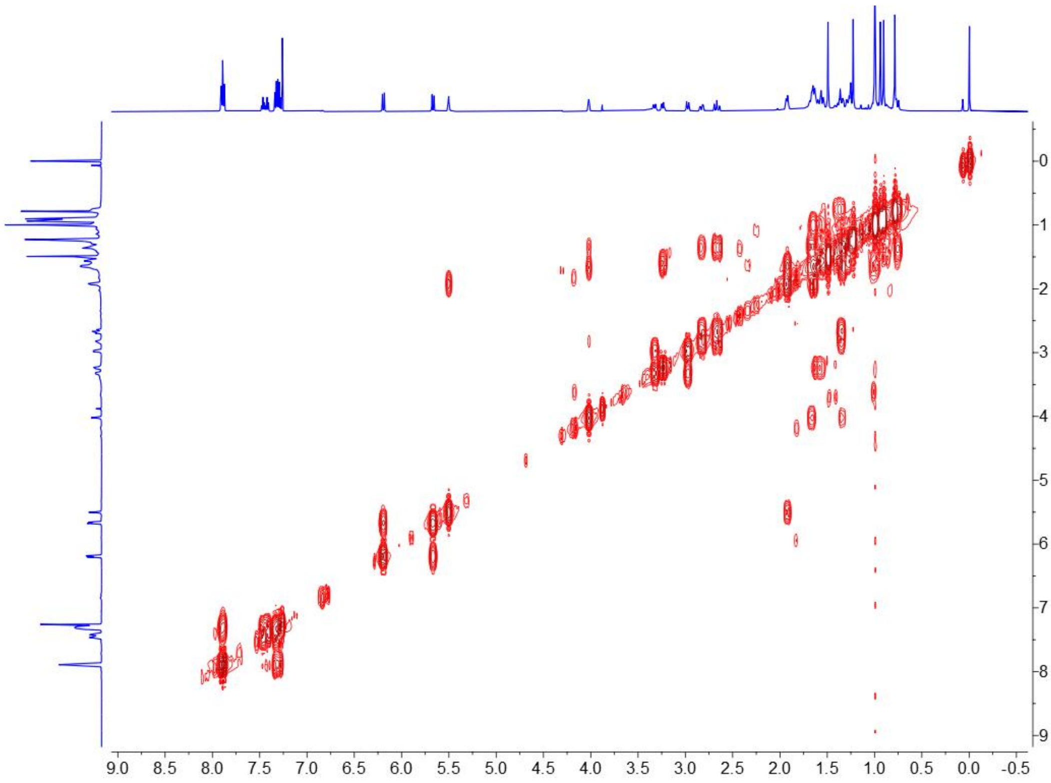

Figure S6-5. The ^{1}H-^{1}H COSY spectrum of compound **6** (600 MHz, $CDCl_3$).

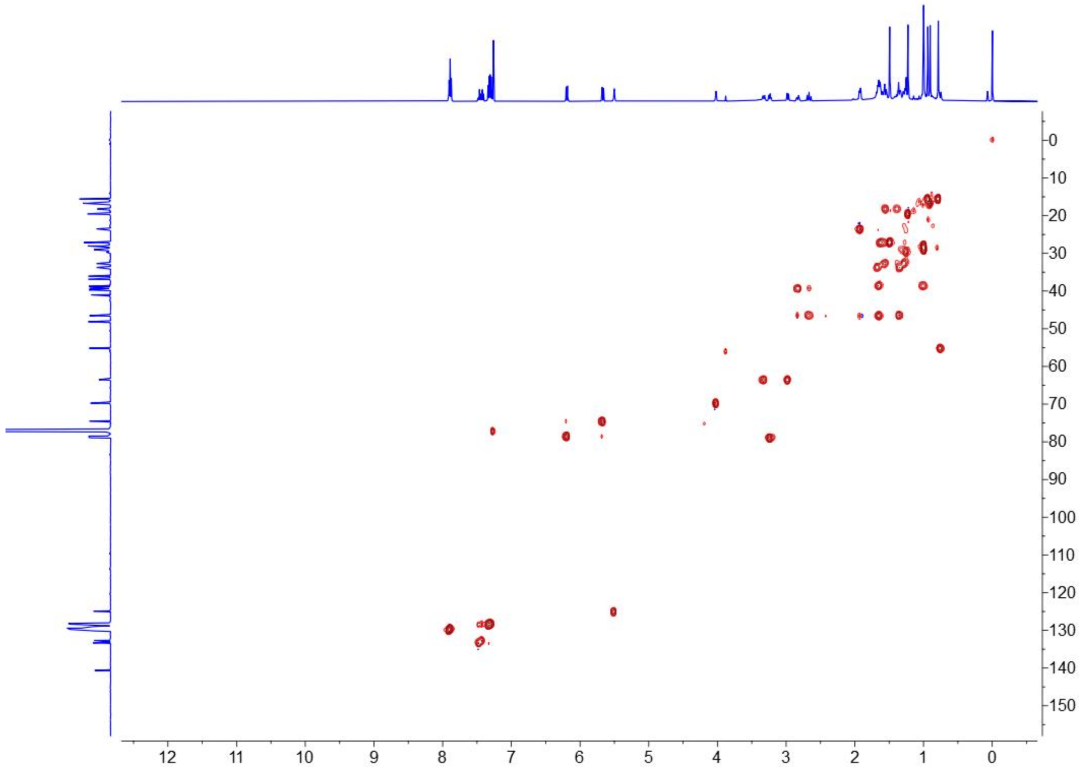

Figure S6-6. The HSQC spectrum of compound **6** (600/151 MHz, $CDCl_3$).

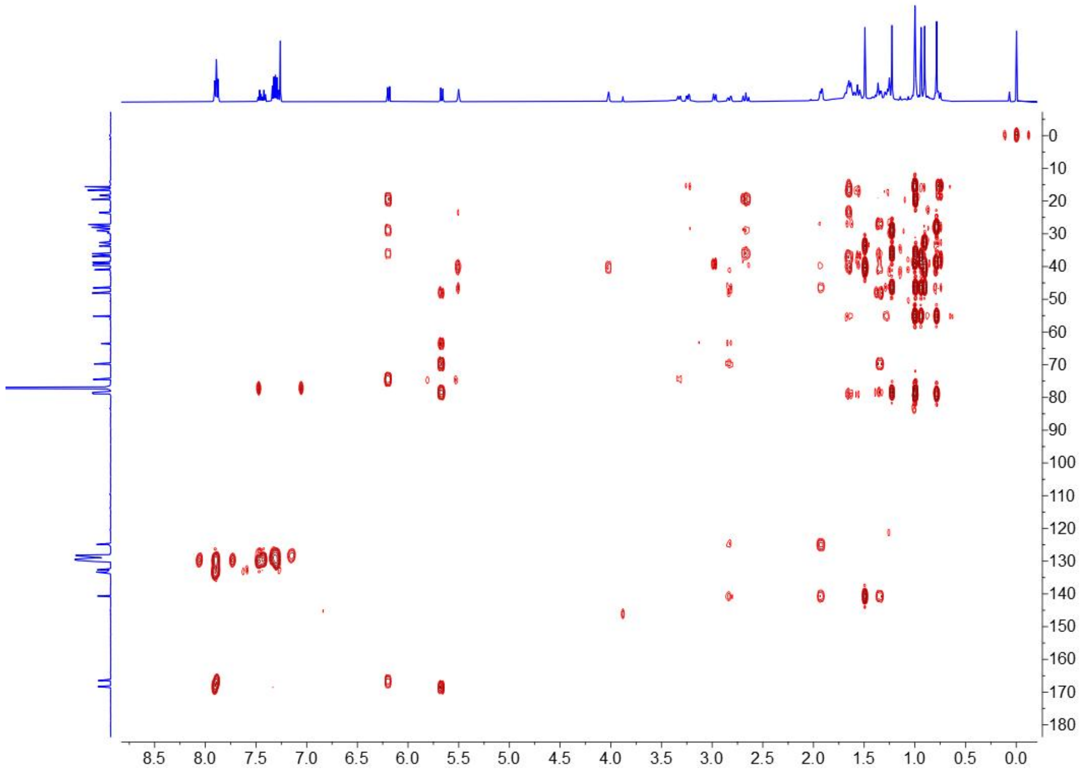

Figure S6-7. The HMBC spectrum of compound **6** (600/151 MHz, $CDCl_3$).

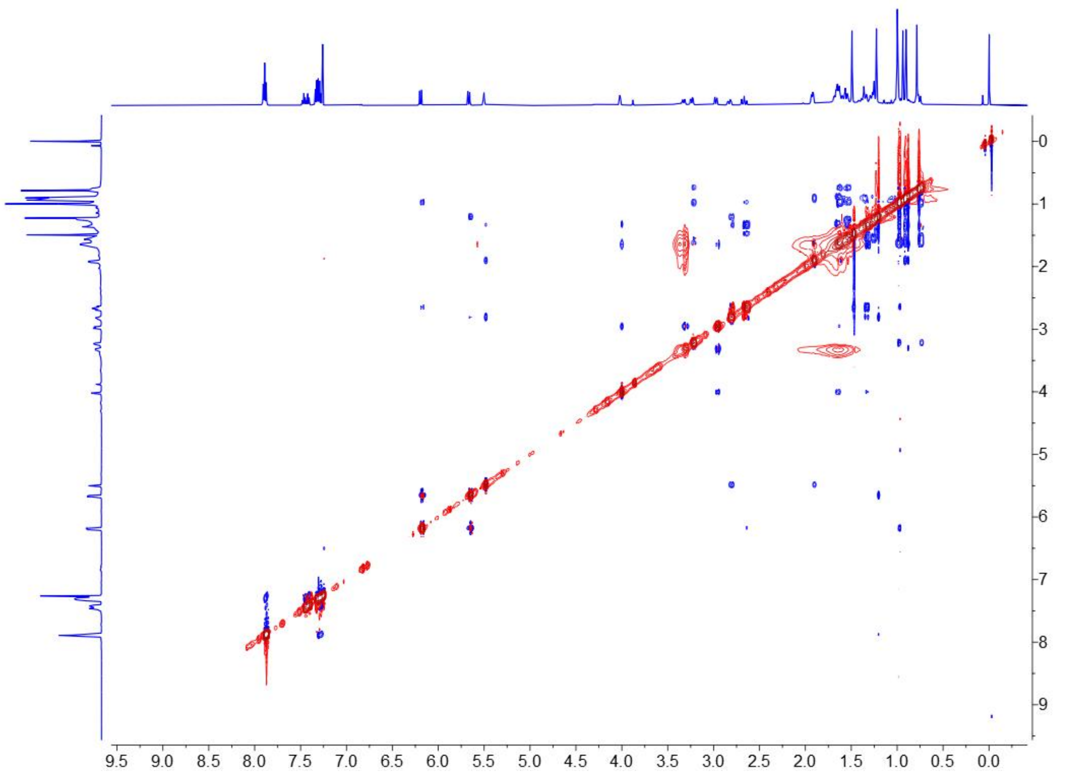

Figure S6-8. The NOESY spectrum of compound **6** (600 MHz, $CDCl_3$).

7. Spectroscopic data for compound 7

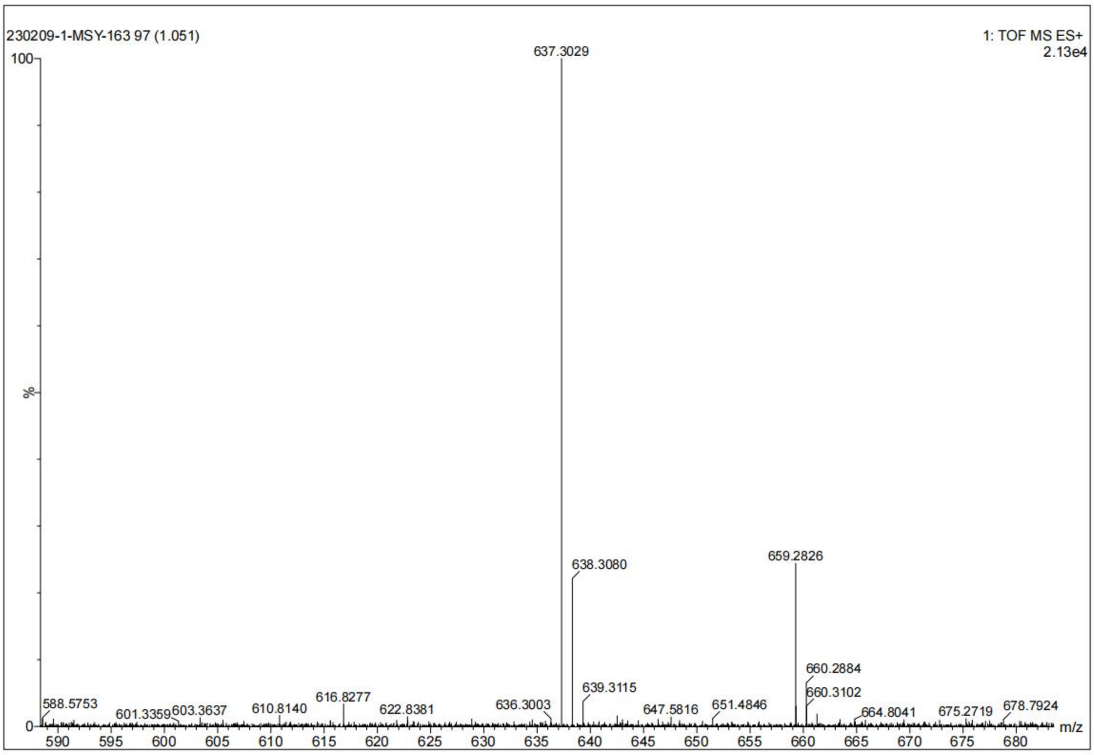

Figure S7-1. The HR-ESI-MS spectrum of compound **7.**

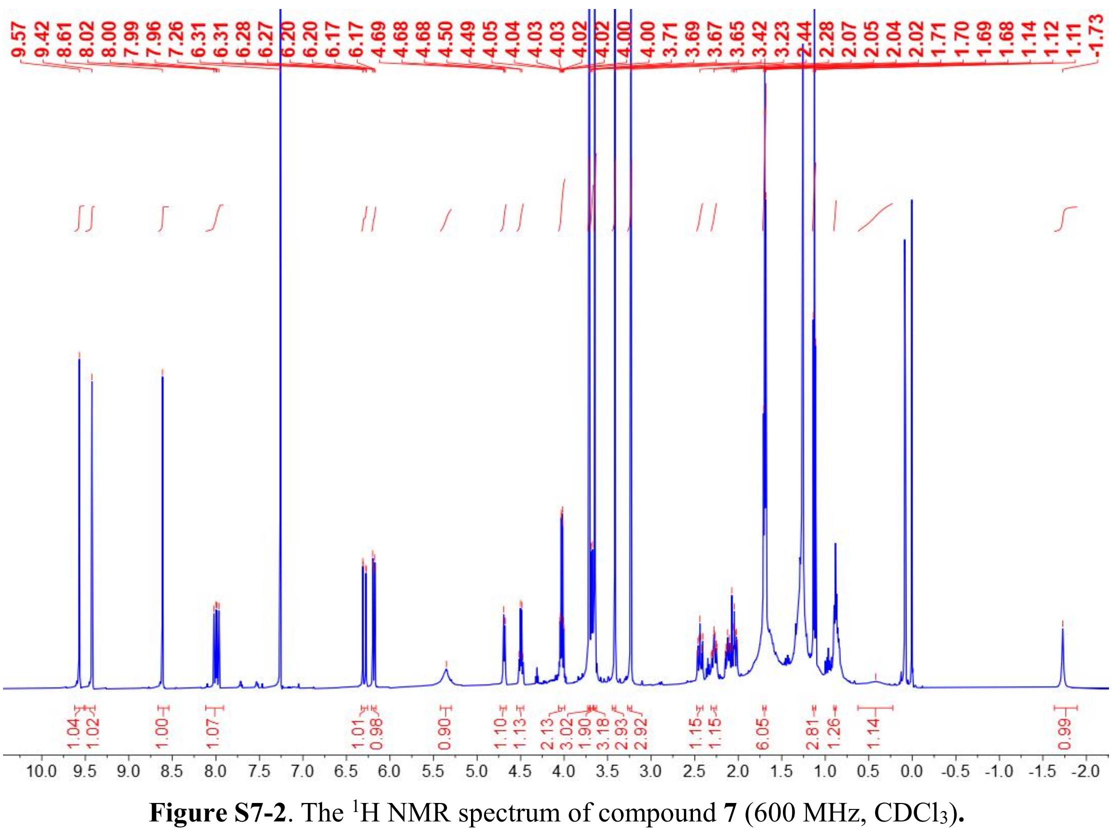

Figure S7-2. The ^{1}H NMR spectrum of compound **7** (600 MHz, $CDCl_3$)**.**

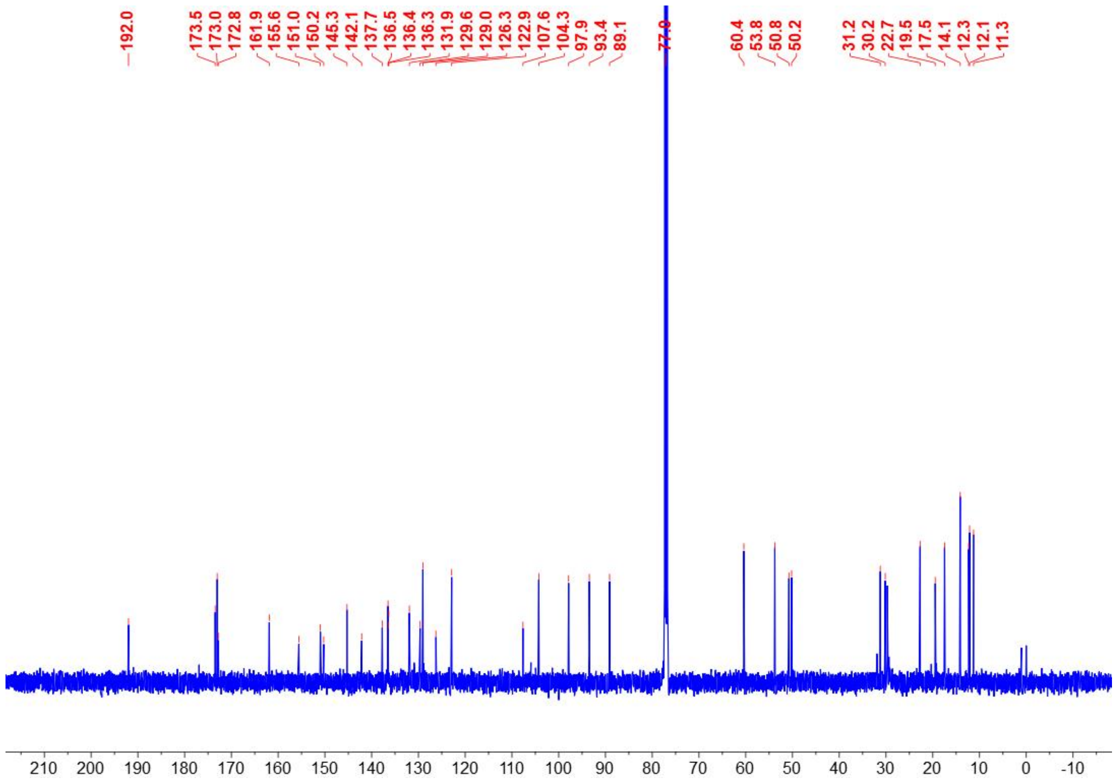

Figure S7-3. The ^{13}C NMR spectrum of compound **7** (151 MHz, $CDCl_3$).

.

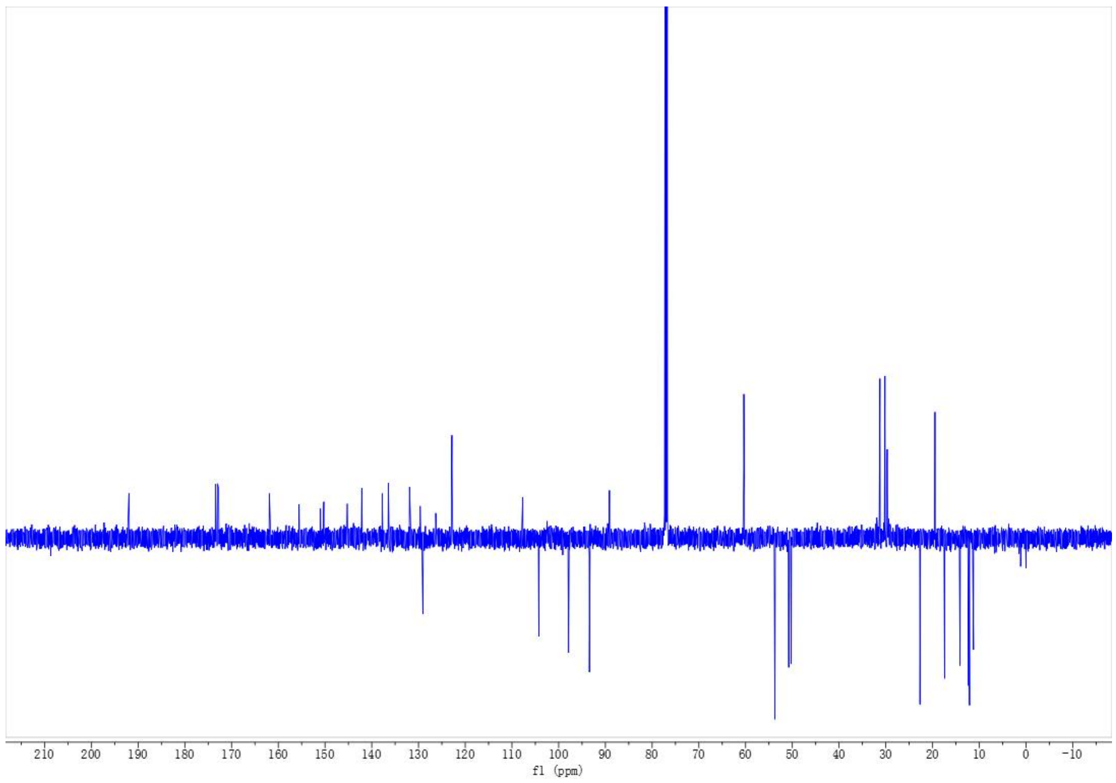

Figure S7-4. The DEPT 135 spectrum of compound **7** (151 MHz, $CDCl_3$).

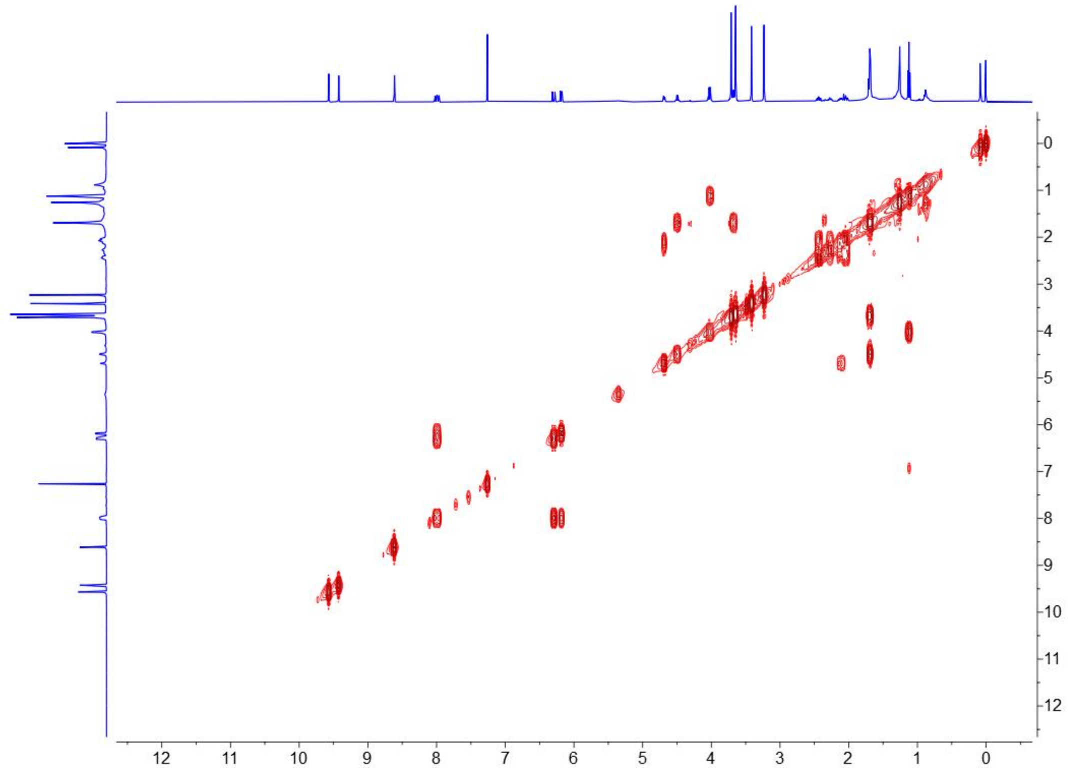

Figure S7-5. The ^1H-^1H COSY spectrum of compound **7** (600 MHz, $CDCl_3$).

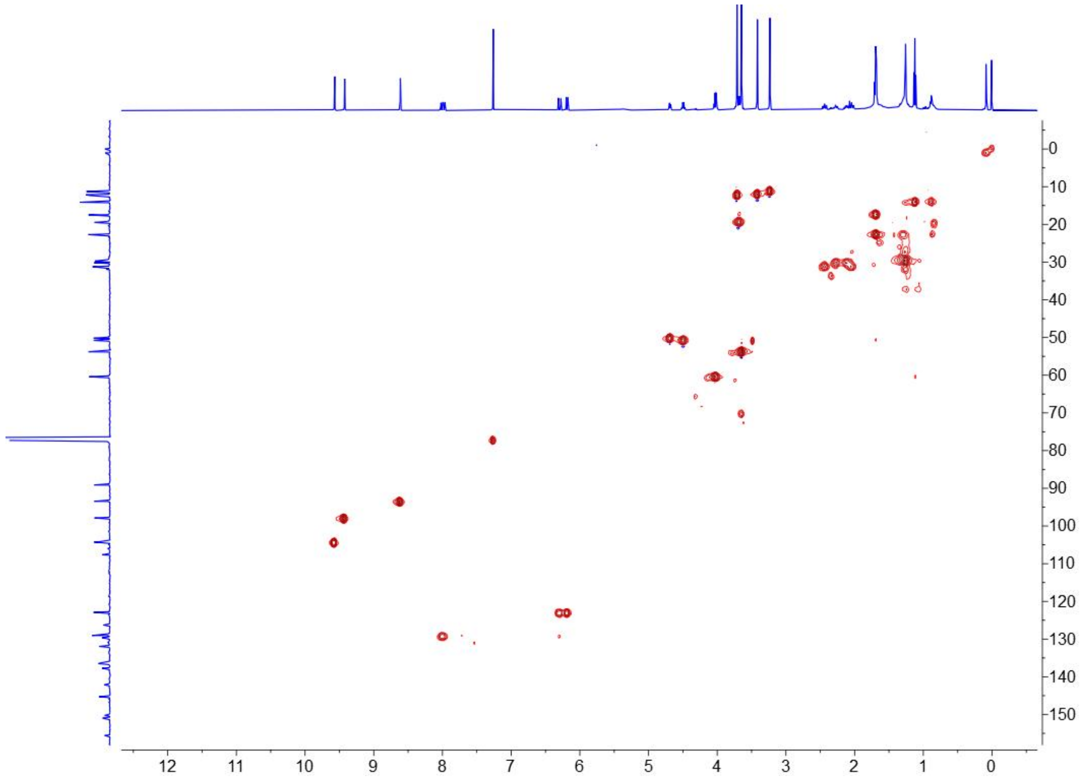

Figure S7-6. The HSQC spectrum of compound **7** (600/151 MHz, $CDCl_3$)

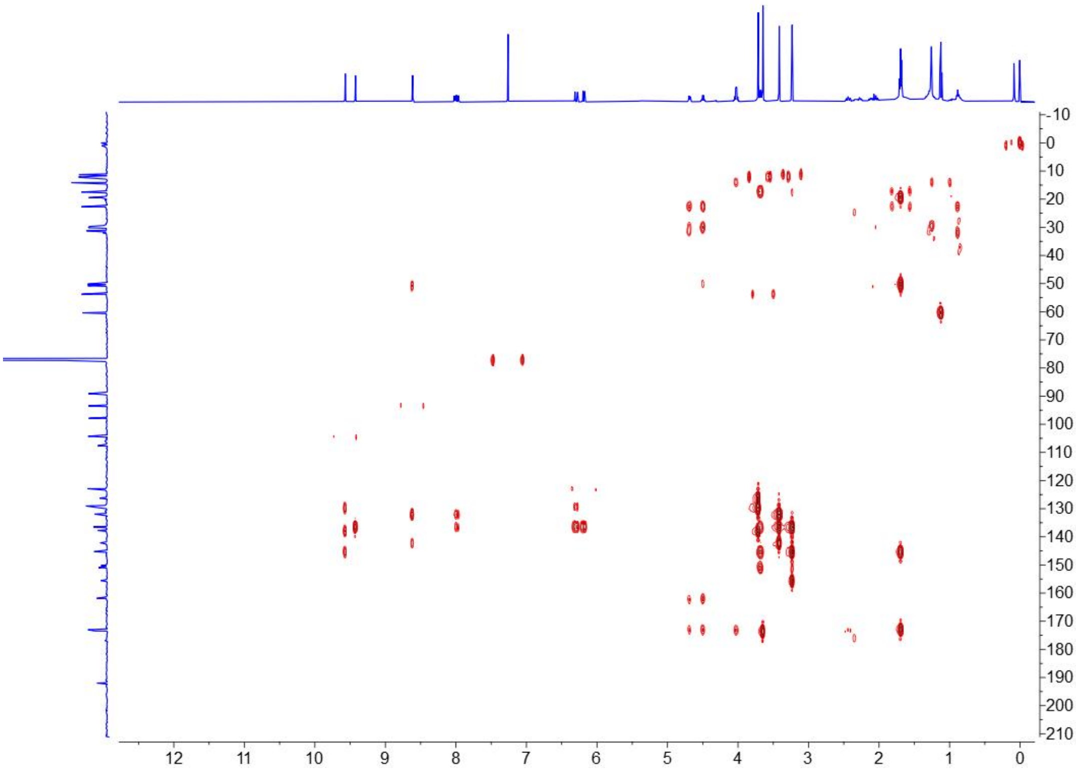

Figure S7-7. The HMBC spectrum of compound **7** (600/151 MHz, $CDCl_3$)

8.

Spectroscopic data for compound 8

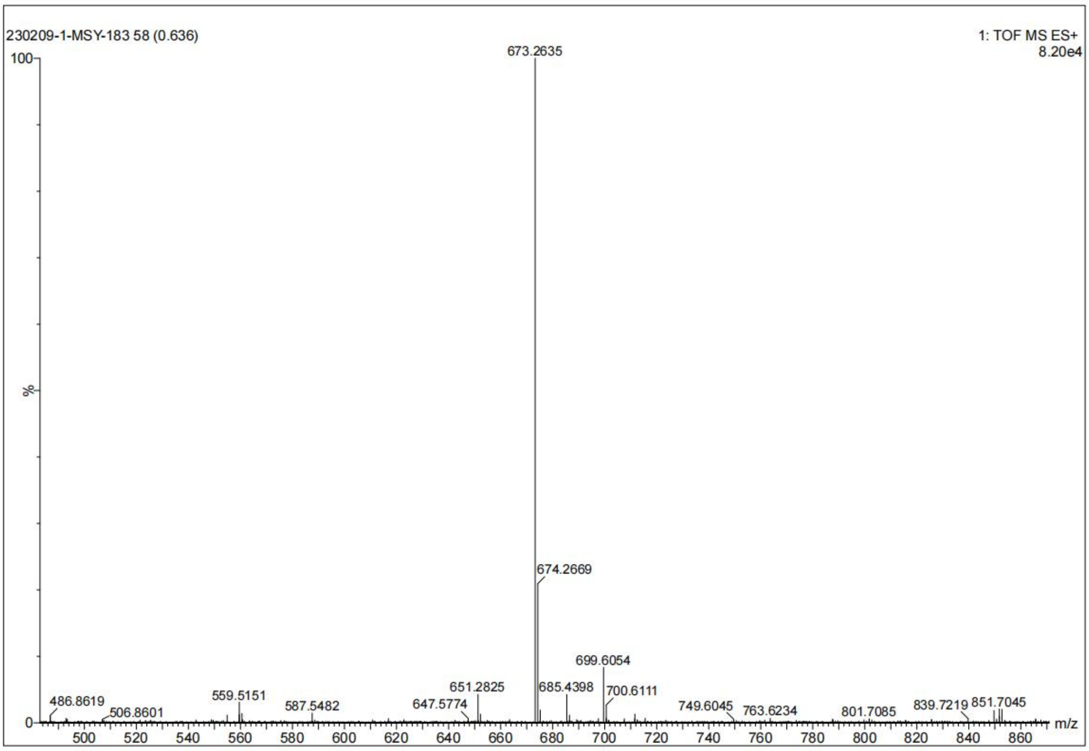

Figure S8-1. The HR-ESI-MS spectrum of compound **8.**

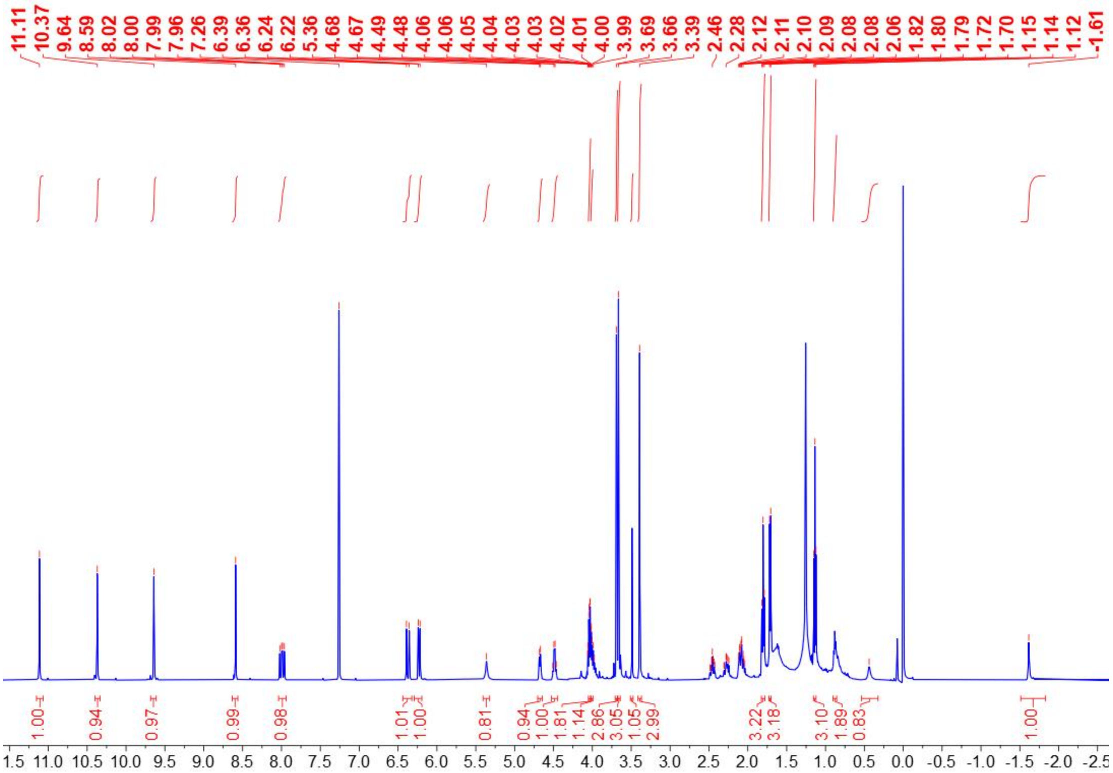

Figure S8-2. The ^{1}H NMR spectrum of compound **8** (600 MHz, $CDCl_3$).

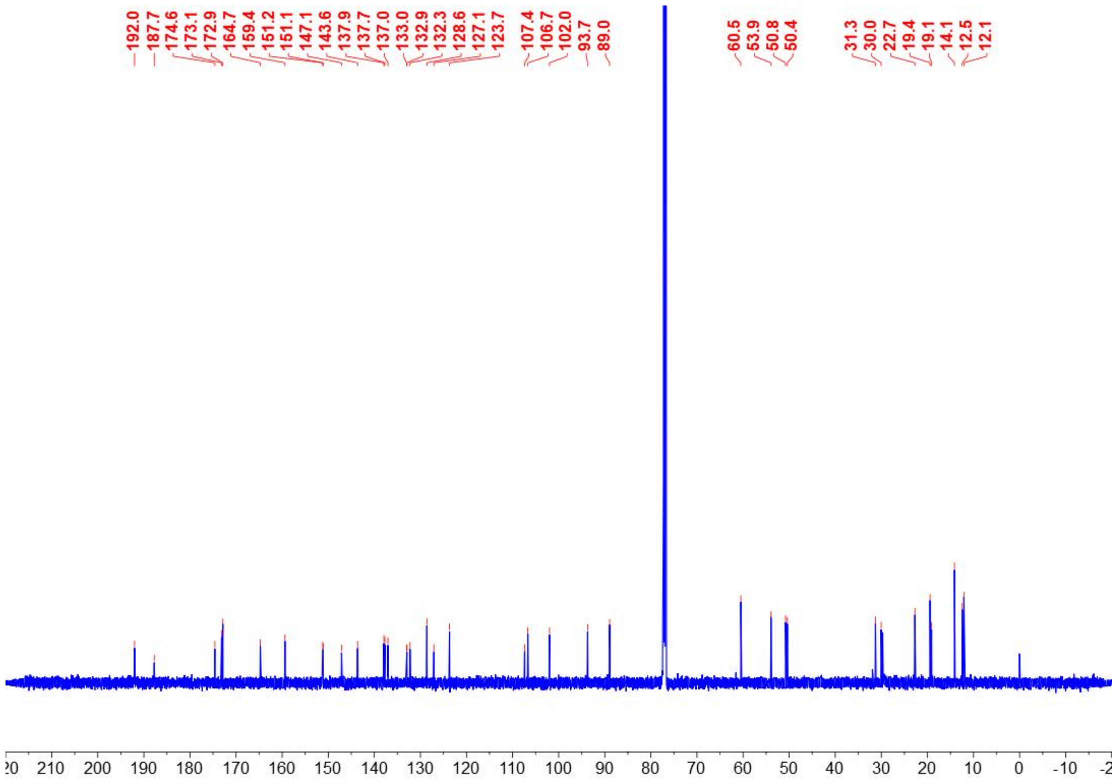

Figure S8-3. The ^{13}C NMR spectrum of compound **8** (151 MHz, $CDCl_3$).

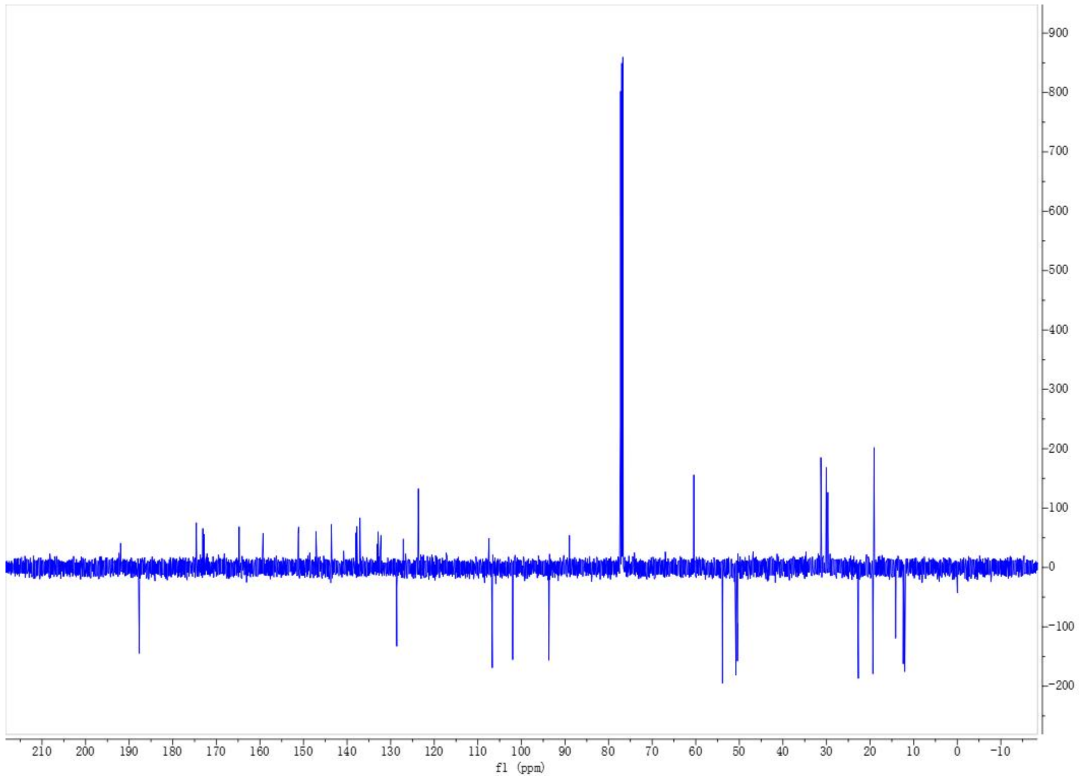

Figure S8-4. The DEPT 135 spectrum of compound **8** (151 MHz, $CDCl_3$).

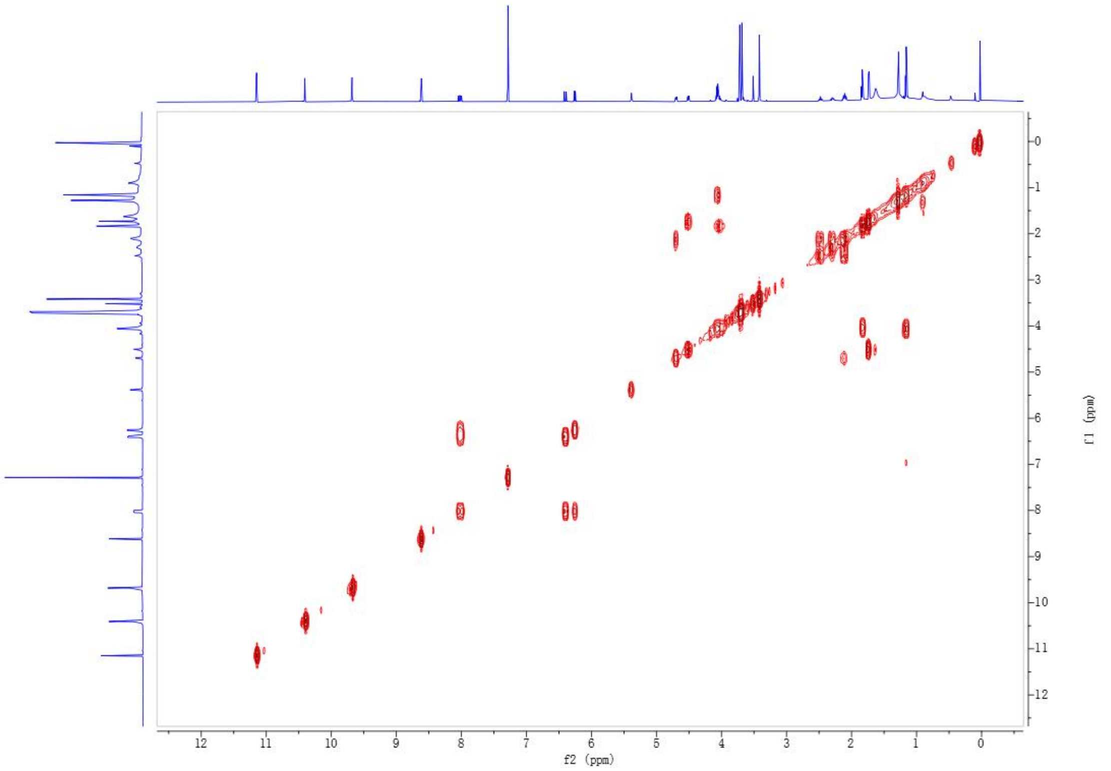

Figure S8-5. The ^1H-^1H COSY spectrum of compound **8** (600 MHz, $CDCl_3$).

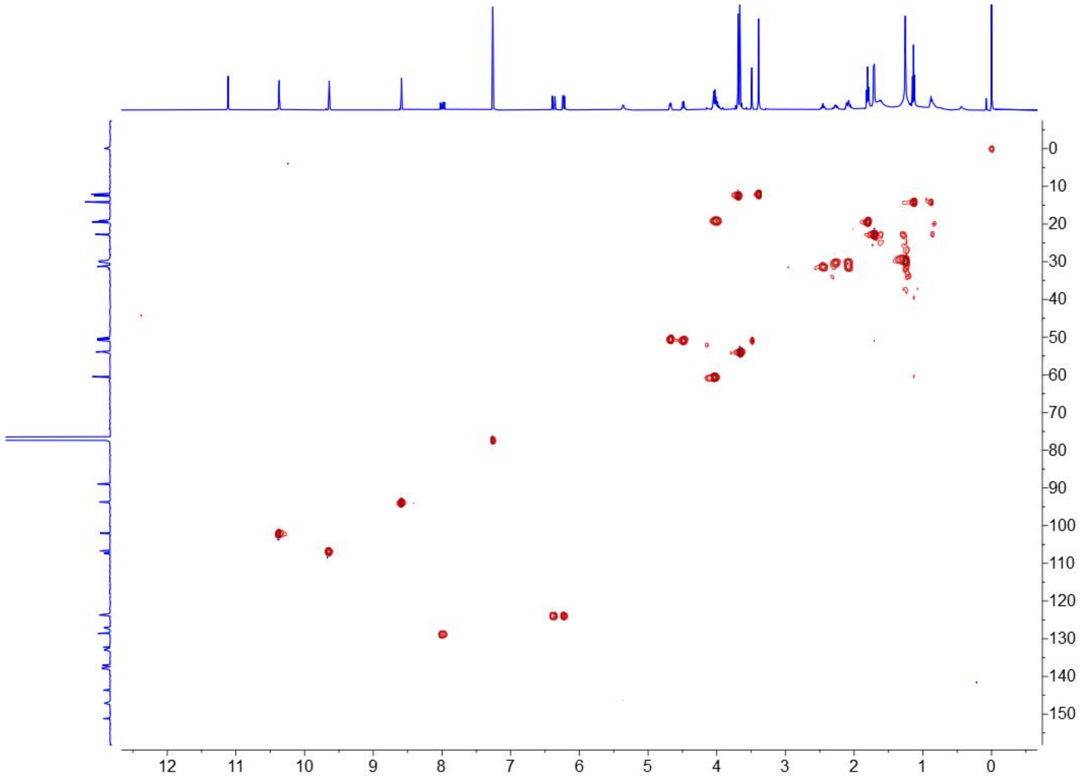

Figure S8-6. The HSQC spectrum of compound **8** (600/151 MHz, $CDCl_3$).

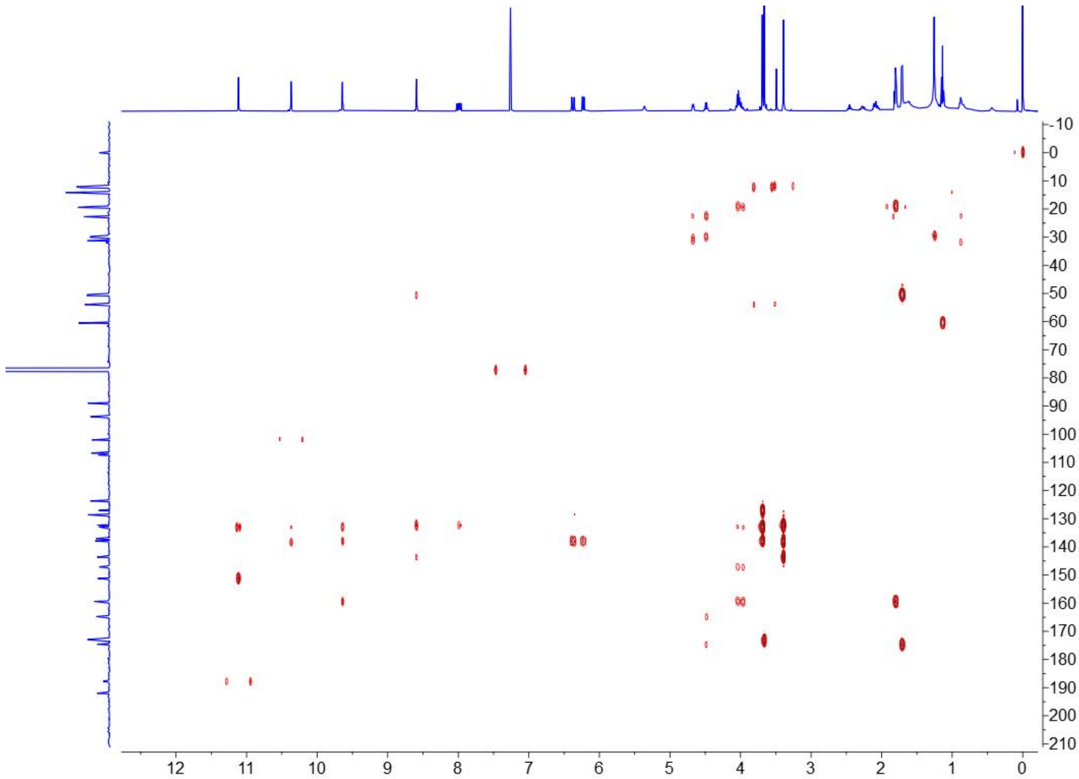

Figure S8-7. The HMBC spectrum of compound **8** (600/151 MHz, $CDCl_3$).

9.

Spectroscopic data for compound 9

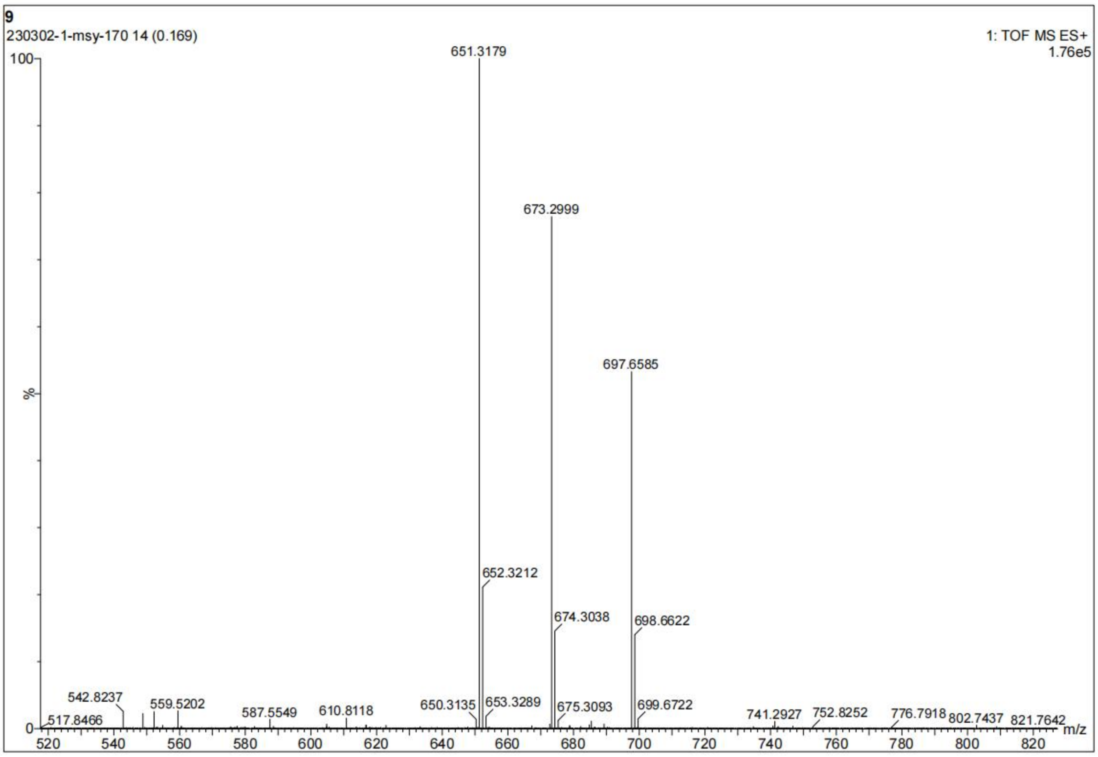

Figure S9-1. The HR-ESI-MS spectrum of compound **9.**

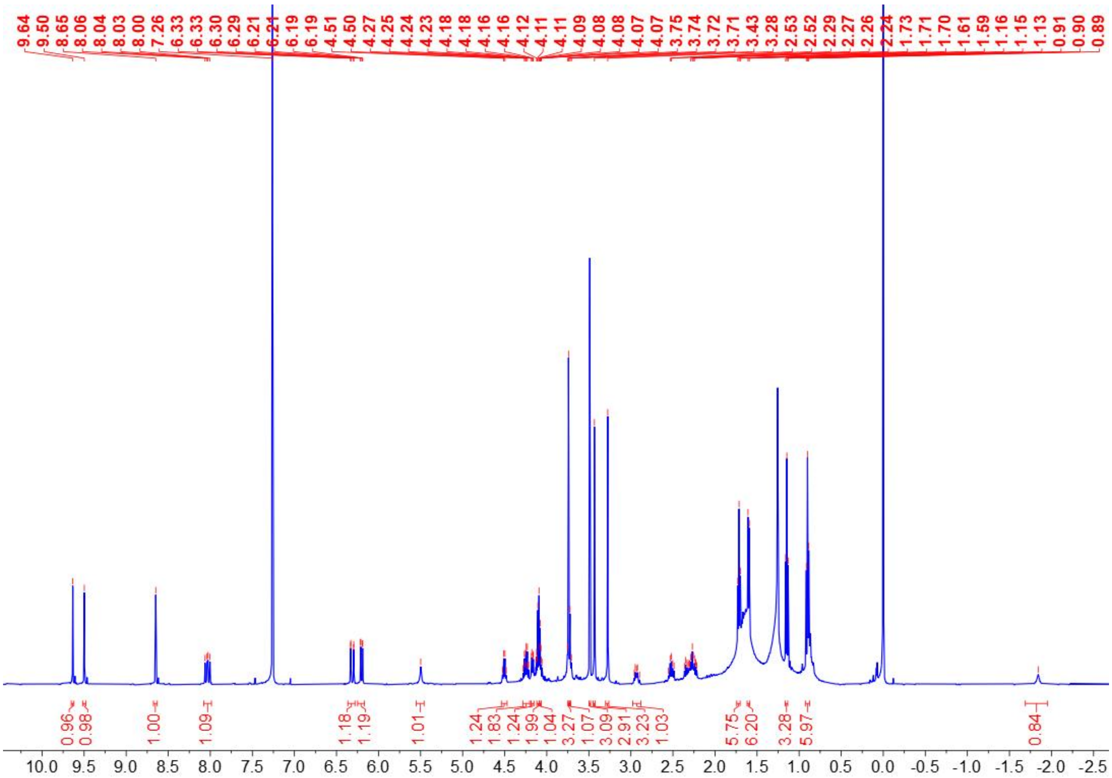

Figure S9-2. The ^{1}H NMR spectrum of compound **9** (600 MHz, $CDCl_3$).

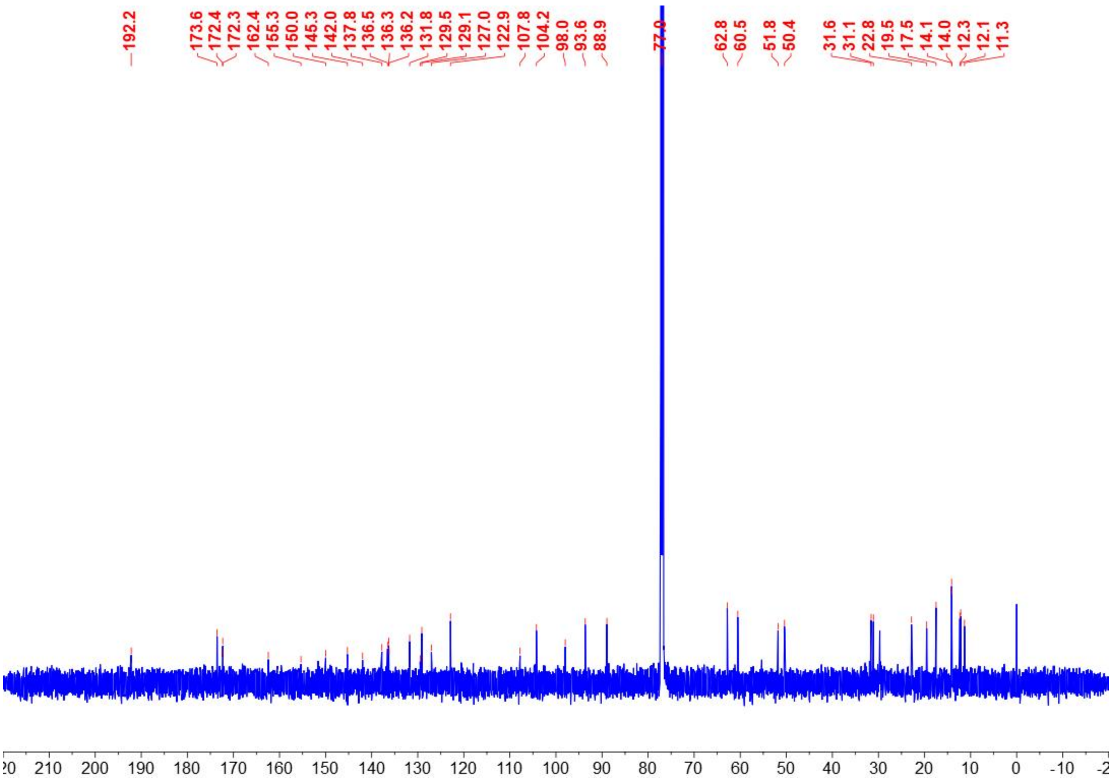

Figure S9-3. The ^{13}C NMR spectrum of compound **9** (151 MHz, $CDCl_3$).

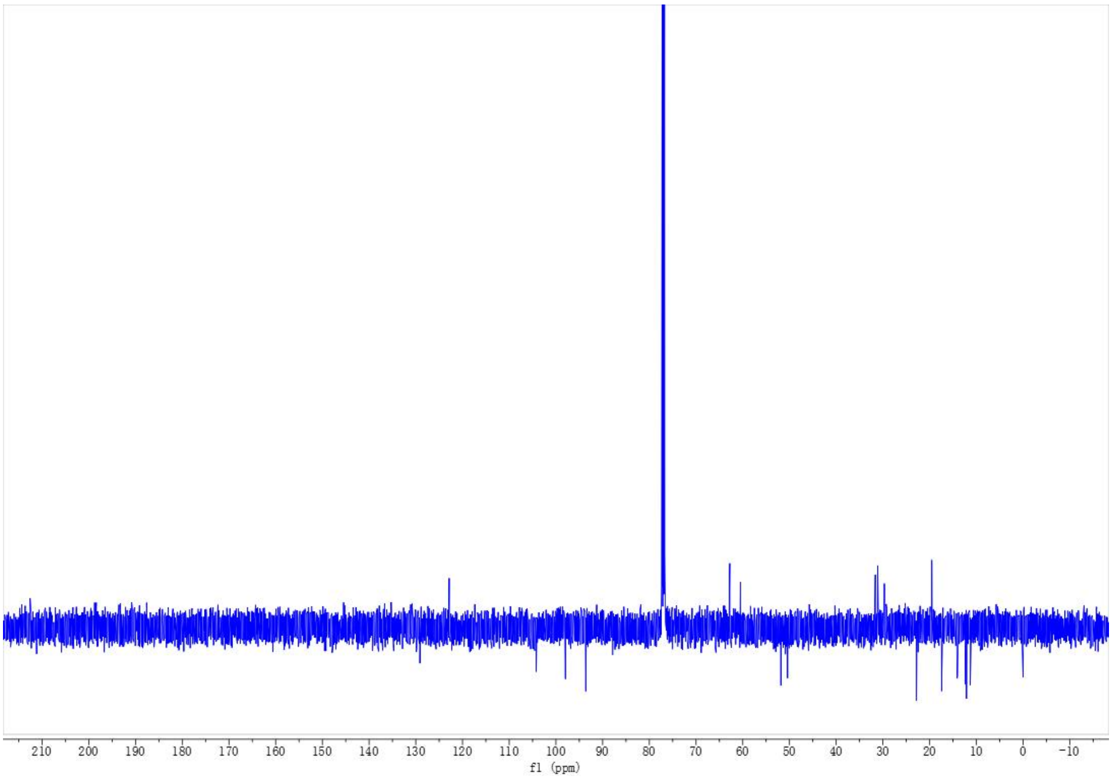

Figure S9-4. The DEPT 135 spectrum of compound **9** (151 MHz, $CDCl_3$).

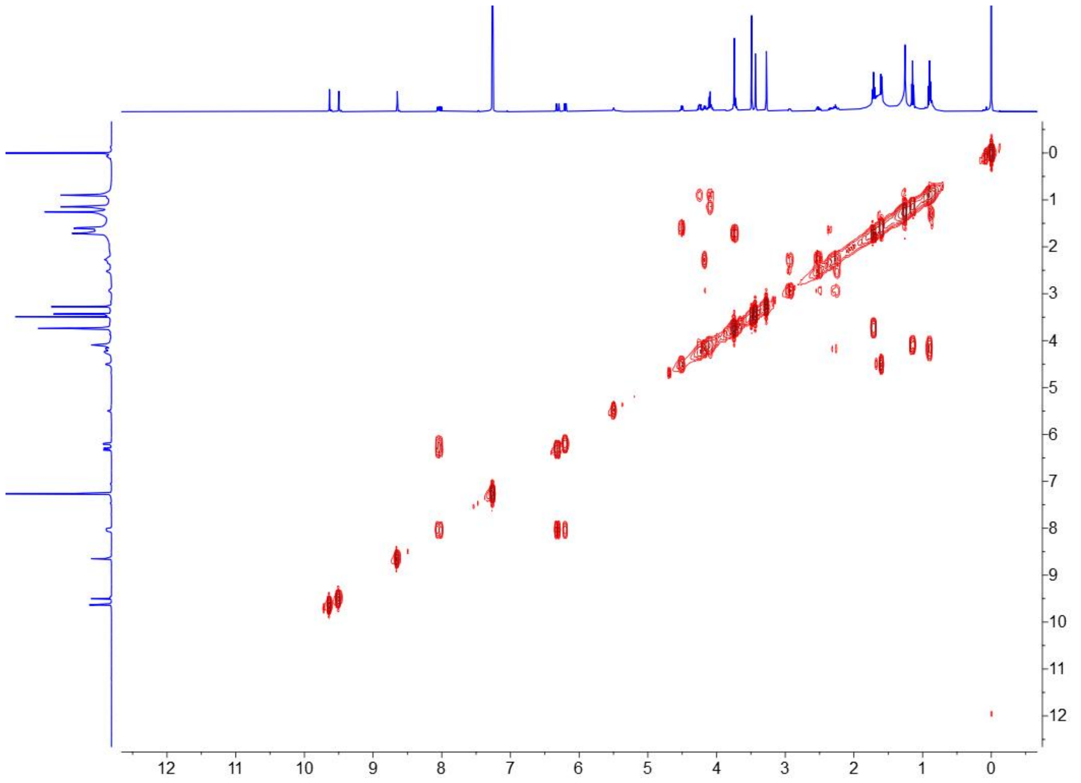

Figure S9-5. The ^{1}H-^{1}H COSY spectrum of compound **9** (600 MHz, $CDCl_3$).

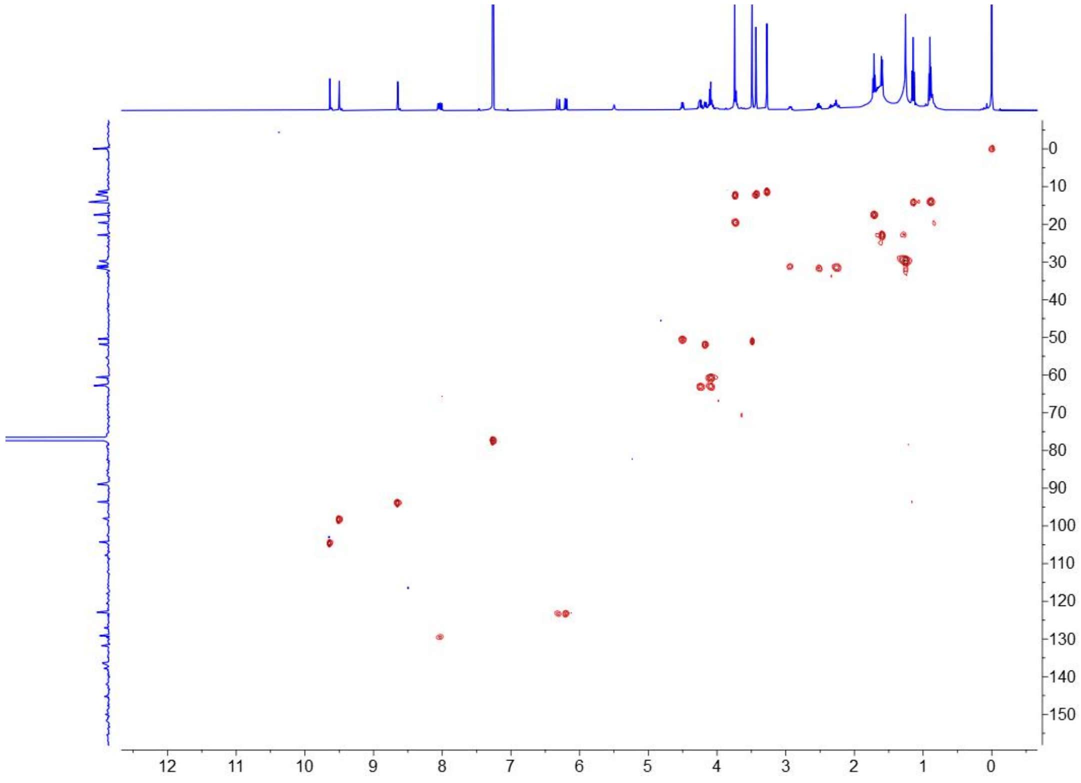

Figure S9-6. The HSQC spectrum of compound **9** (600/151 MHz, $CDCl_3$).

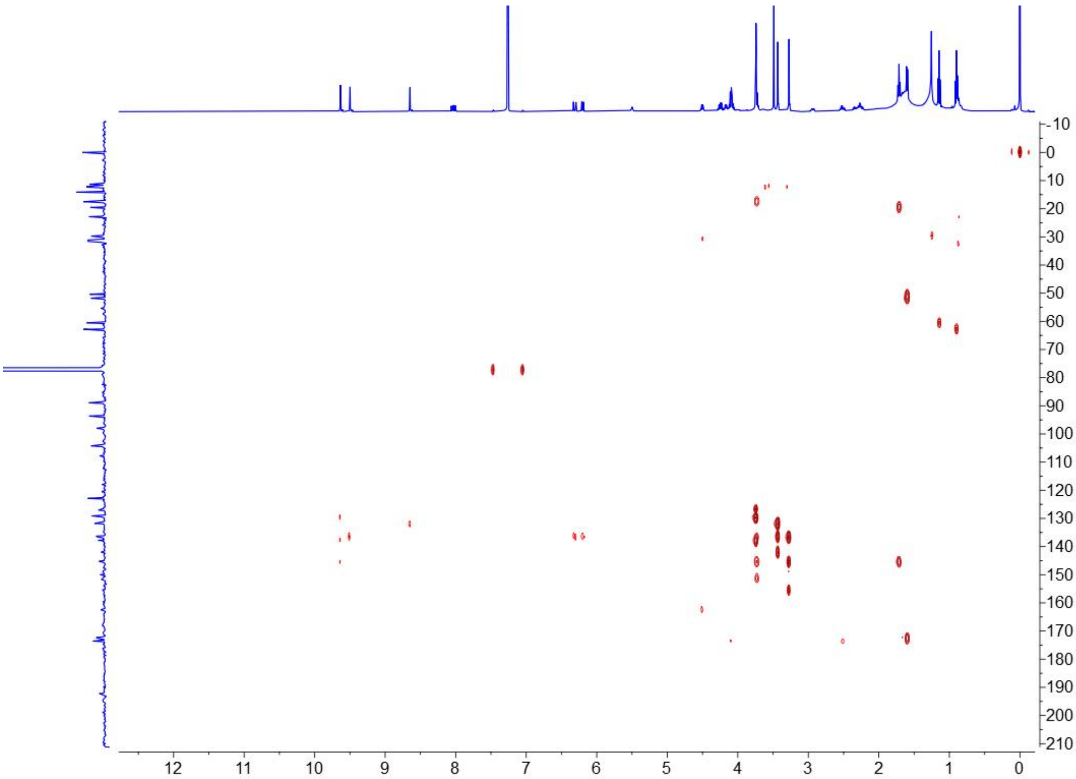

Figure S9-7. The HMBC spectrum of compound **9** (600/151 MHz, $CDCl_3$).

10.

Spectroscopic data for compound 10

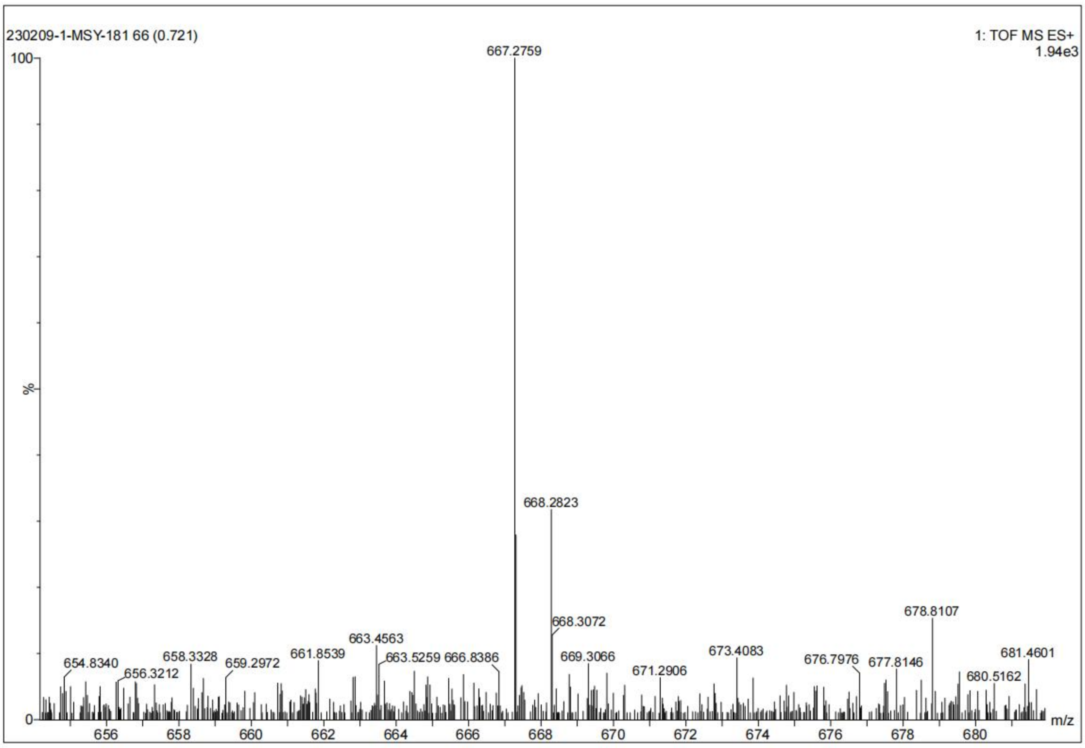

Figure S10-1. The HR-ESI-MS spectrum of compound **10**.

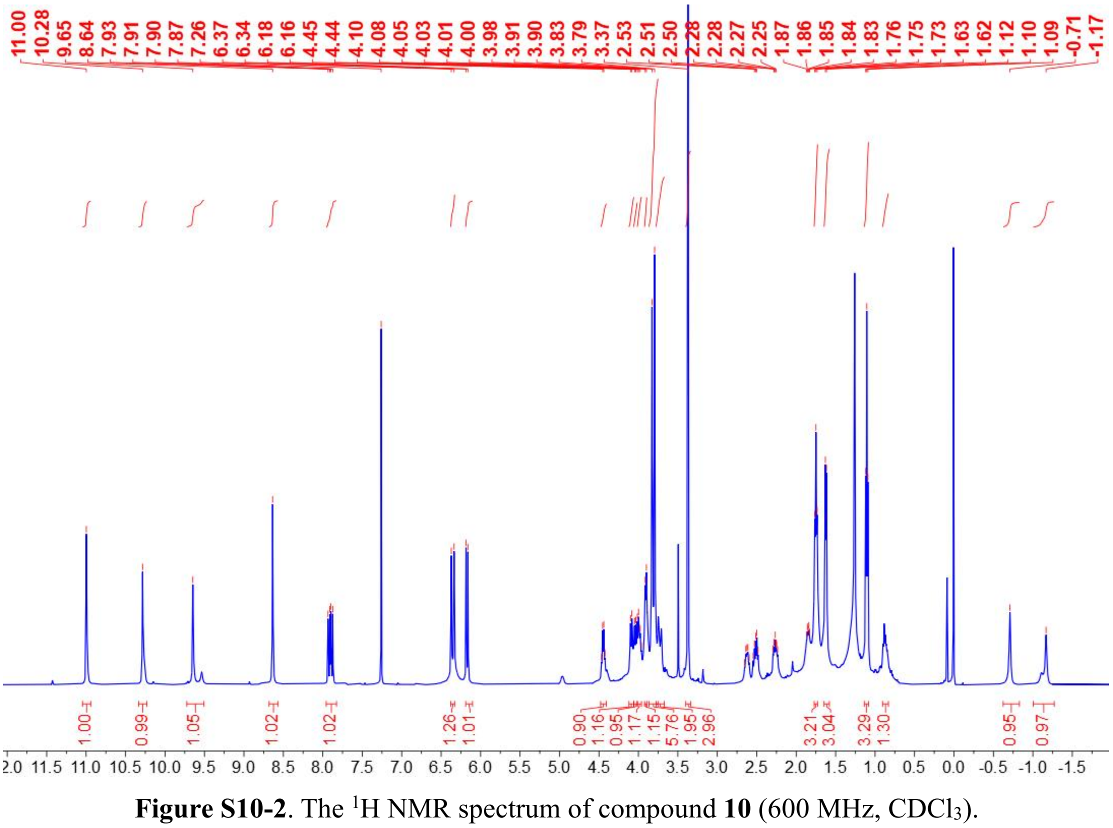

Figure S10-2. The ^{1}H NMR spectrum of compound **10** (600 MHz, $CDCl_3$).

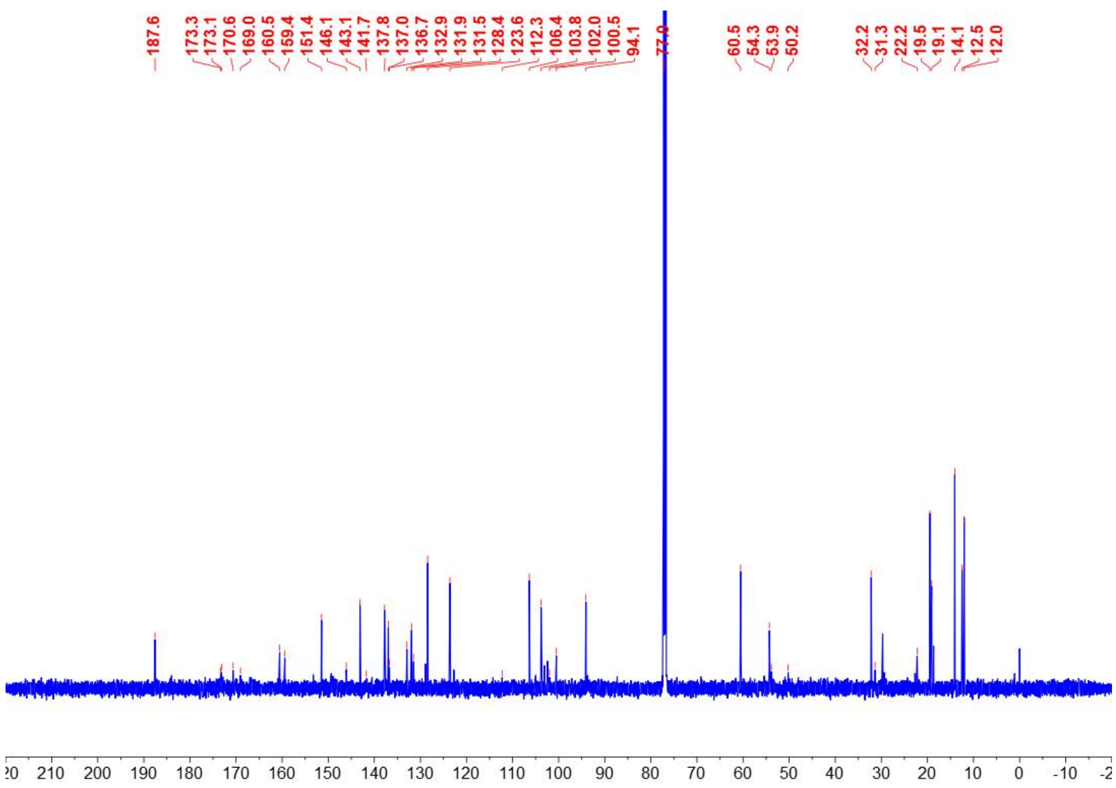

Figure S10-3. The ^{13}C NMR spectrum of compound **10** (151 MHz, $CDCl_3$).

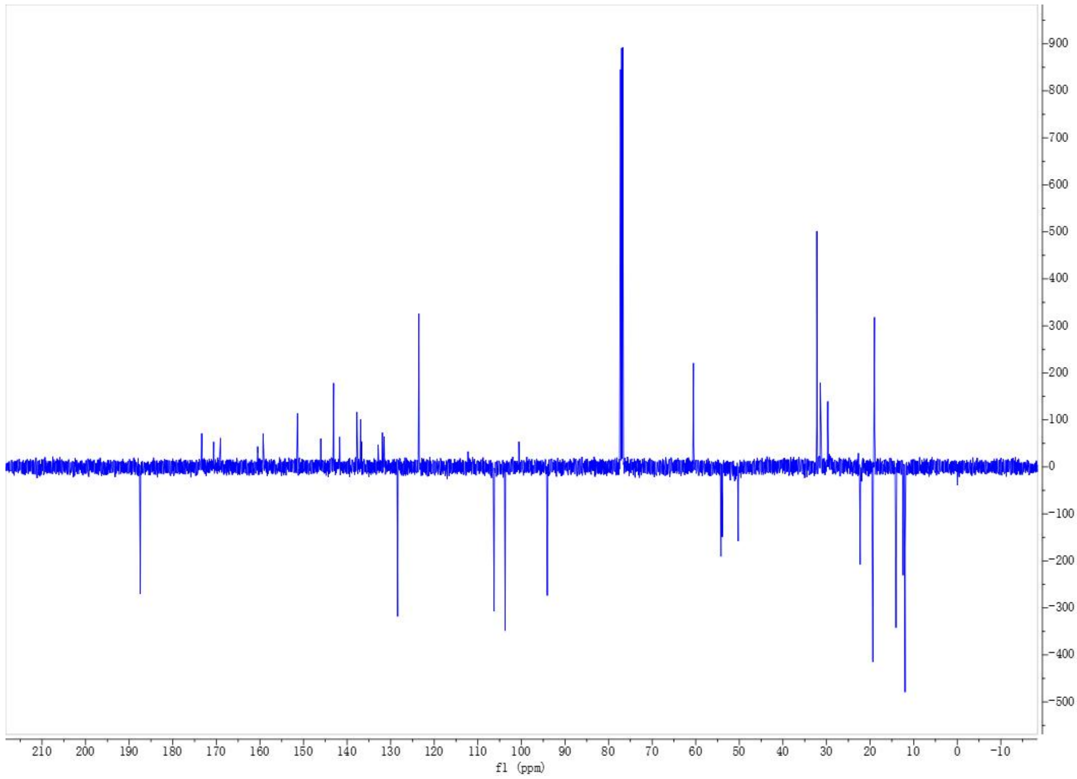

Figure S10-4. The DEPT 135 spectrum of compound **10** (151 MHz, $CDCl_3$).

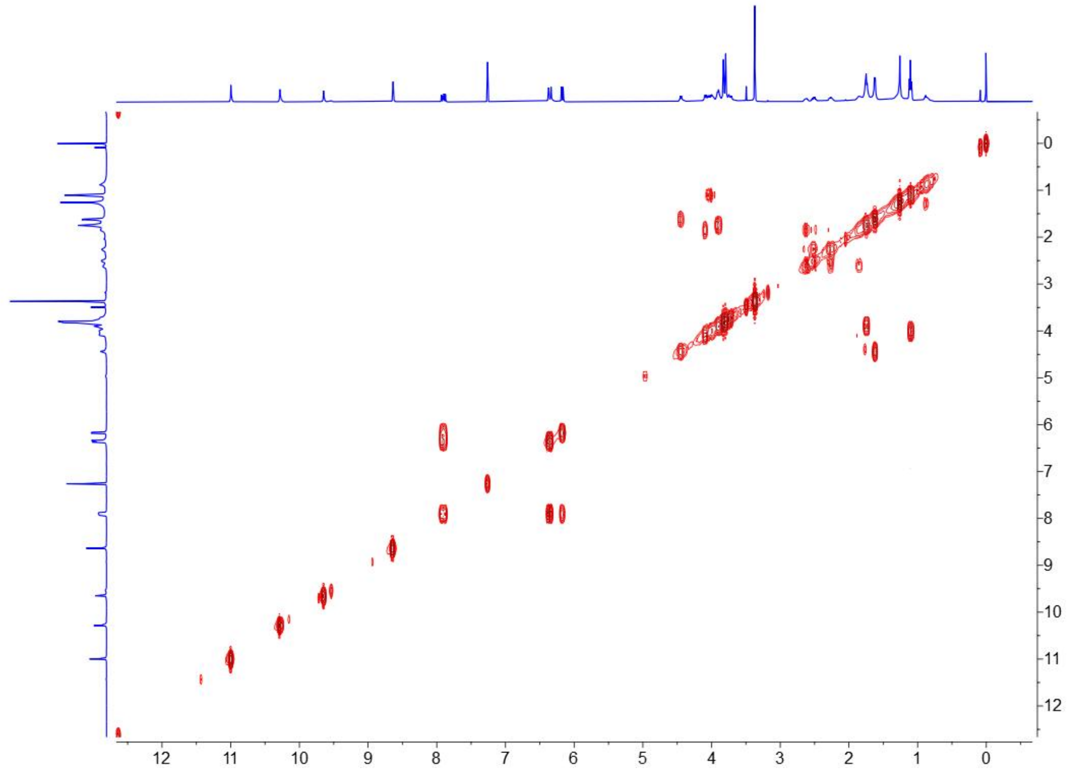

Figure S10-5. The ^{1}H-^{1}H COSY spectrum of compound **10** (600 MHz, $CDCl_3$).

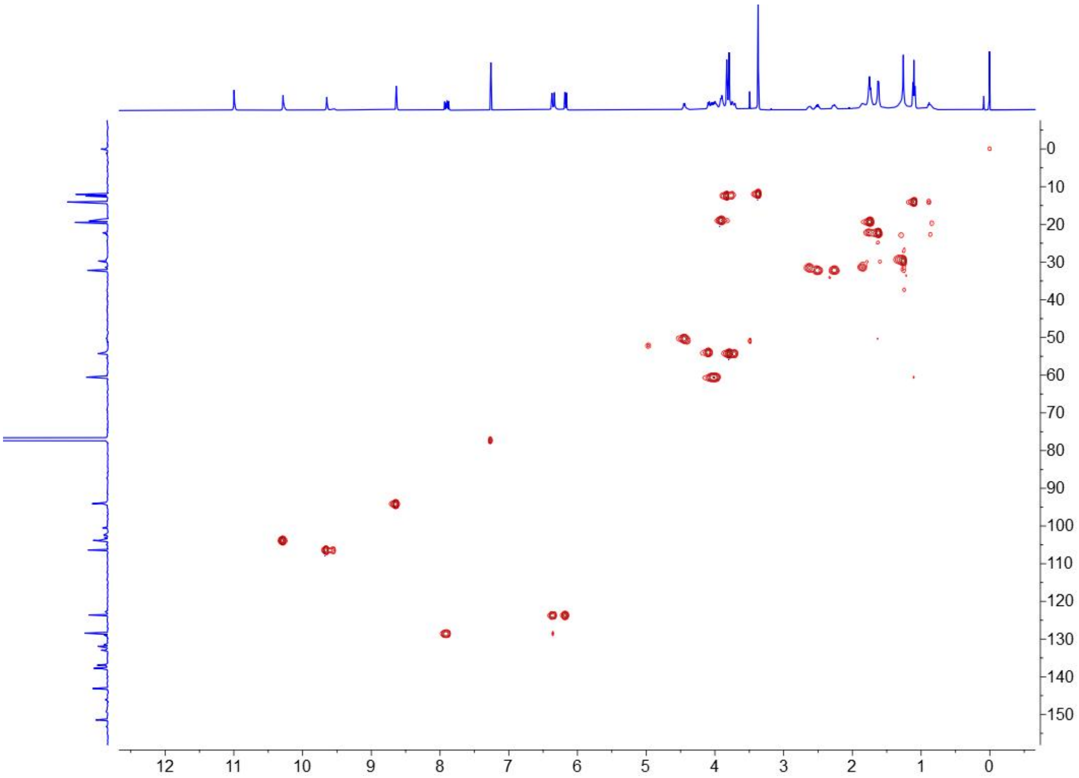

Figure S10-6. The HSQC spectrum of compound **10** (600/151 MHz, $CDCl_3$).

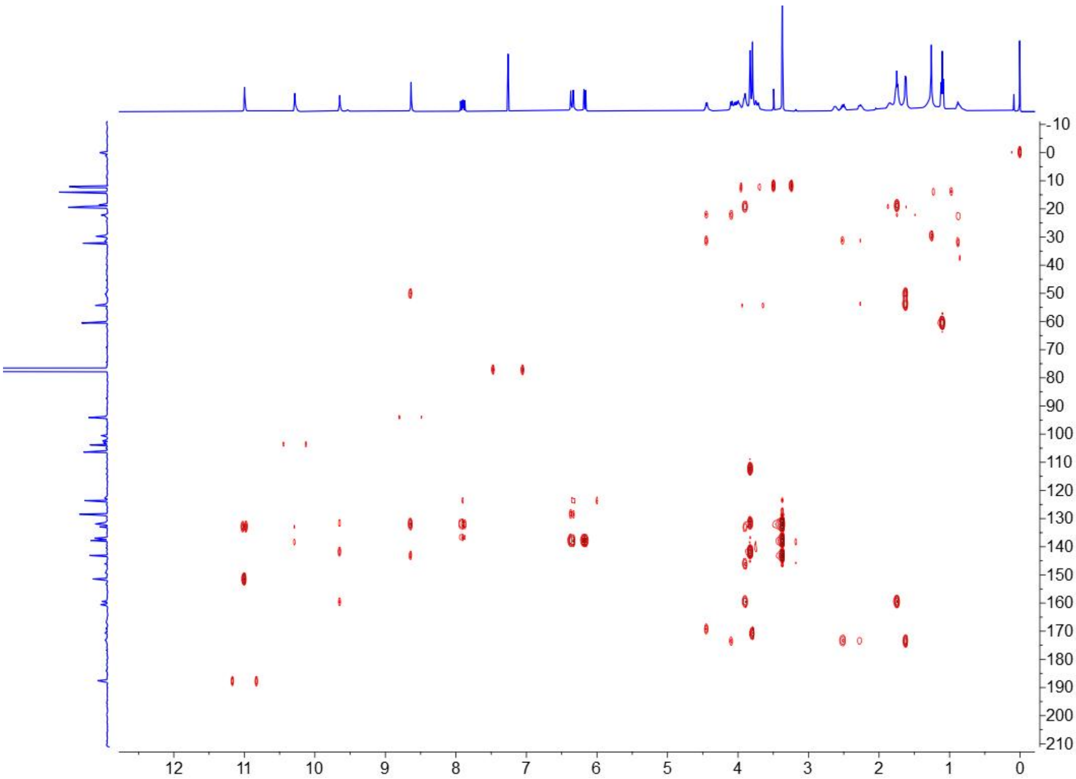

Figure S10-7. The HMBC spectrum of compound **10** (600/151 MHz, $CDCl_3$).

11. The scheme of extraction and isolation

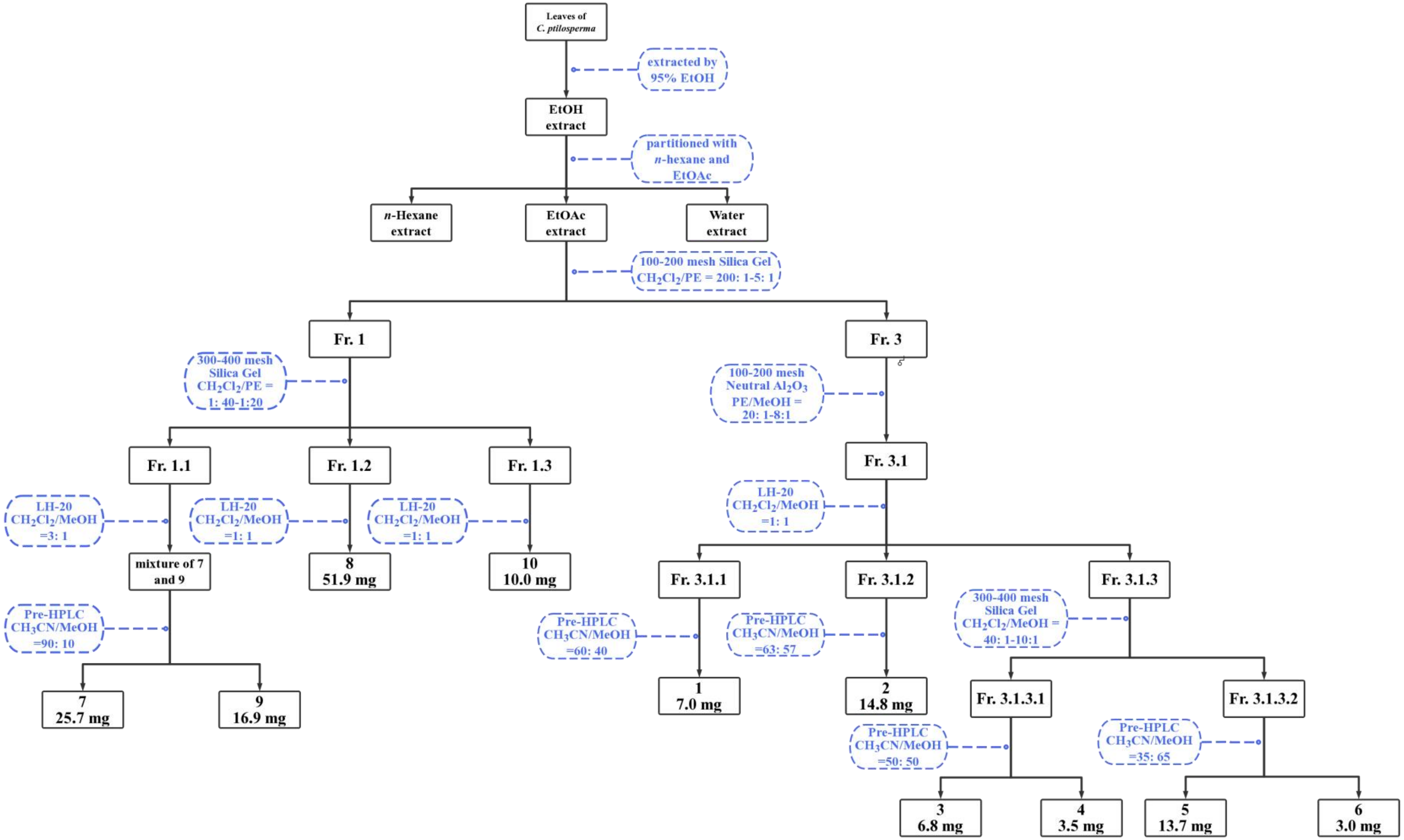

Figure S11. The scheme of extraction and isolation.
